# A data-driven architecture using natural language processing to improve phenotyping efficiency and accelerate genetic diagnoses of rare disorders

**DOI:** 10.1016/j.xhgg.2021.100035

**Published:** 2021-05-11

**Authors:** Jignesh R. Parikh, Casie A. Genetti, Asli Aykanat, Catherine A. Brownstein, Klaus Schmitz-Abe, Morgan Danowski, Andrew Quitadomo, Jill A. Madden, Calum Yacoubian, Richard Gain, Tessa Williams, Mary Meskell, Andrew Brown, Alison Frith, Shira Rockowitz, Piotr Sliz, Pankaj B. Agrawal, Thomas Defay, Paul McDonagh, John Reynders, Sebastien Lefebvre, Alan H. Beggs

**Affiliations:** 1 J Square Labs, LLC, Natick, MA 01760, USA;; 2 The Manton Center for Orphan Disease Research, Division of Genetics and Genomics, Boston Children’s Hospital, Harvard Medical School, Boston, MA 02115, USA;; 3 Alexion Pharmaceuticals, Inc., Boston, MA 02210, USA;; 4 Computational Health Informatics Program, Boston Children’s Hospital, Harvard Medical School, Boston, MA 02115, USA;; 5 Clinithink, Ltd., London N1 6DR, UK;; 6 Division of Newborn Medicine, Boston Children’s Hospital, Harvard Medical School, Boston, MA 02115, USA; 7 These authors contributed equally to this work; 8 Present address: Sema4, Stamford, CT 06902, USA.; 9 Present address: Latent Strategies, LLC, Newton, MA 02465, USA.

## Abstract

Effective genetic diagnosis requires the correlation of genetic variant data with detailed phenotypic information. However, manual encoding of clinical data into machine-readable forms is laborious and subject to observer bias. Natural language processing (NLP) of electronic health records has great potential to enhance reproducibility at scale but suffers from idiosyncrasies in physician notes and other medical records. We developed methods to optimize NLP outputs for automated diagnosis. We filtered NLP-extracted Human Phenotype Ontology (HPO) terms to more closely resemble manually extracted terms and identified filter parameters across a three-dimensional space for optimal gene prioritization. We then developed a tiered pipeline that reduces manual effort by prioritizing smaller subsets of genes to consider for genetic diagnosis. Our filtering pipeline enabled NLP-based extraction of HPO terms to serve as a sufficient replacement for manual extraction in 92% of prospectively evaluated cases. In 75% of cases, the correct causal gene was ranked higher with our applied filters than without any filters. We describe a framework that can maximize the utility of NLP-based phenotype extraction for gene prioritization and diagnosis. The framework is implemented within a cloud-based modular architecture that can be deployed across health and research institutions.

## Introduction

Over the past decade, the introduction of next-generation sequencing has revolutionized the diagnosis and discovery of rare monogenic conditions. Exome sequencing (ES) has been shown to be an effective first-tier test for the diagnosis of a variety of congenital and neurodevelopmental phenotypes.^1,2^ The technical ability to generate high-quality genomic data in a timely manner has reached a plateau, and significant progress has been made in the field of variant interpretation, particularly in the coding region of the genome.^3^ Despite these advances, the diagnostic rate of ES remains relatively low at 25%–50%.^2,4,5^ Pathogenic variants in a significant percentage of these undiagnosed cases may be hidden in poorly understood non-coding regions or in the approximately 15,000 genes that have yet to be associated with human disease.^6^ Nevertheless, it is clear that a lack of accurate and deep phenotyping to correlate with genotypic findings remains a major issue in variant interpretation, especially in high-throughput clinical diagnostic situations.^7–10^ The process of deep phenotyping, whether through clinical encounter or medical record review, is a labor- and time-intensive process requiring a high degree of expertise.^11,12^

Natural language processing (NLP) has been adopted as a scalable approach to automate the extraction of phenotypic information from electronic health records (EHRs). Standardization of outputs by encoding clinical information using the Human Phenotype Ontology (HPO) in a high-throughput manner has great potential to help shorten the diagnostic odyssey, thereby reducing costs and improving care.^13,14^ However, given idiosyncrasies and variation in the structure and content of different EHR systems and notes from health care providers, it has been a challenge to develop automated phenotyping approaches comparable or superior to manual curation to facilitate the diagnosis of genetic diseases.^11,12^

The goal of this project is to provide a replicable framework to maximize the utility of NLP-based phenotype extraction from EHRs for use with gene prioritization algorithms. Here, we compare the efficacy of a gene prioritization tool, Exomiser,^15^ in correctly identifying the disease-causing gene in previously diagnosed children using manual phenotyping by an expert curator versus automated NLP extraction from the EHR. Utilizing differences identified between the manual and NLP-extracted HPO terms, we constructed a tiered pipeline that automatically filtered NLP-extracted HPO terms and improved gene prioritization in prospectively evaluated cases.

Our approach enabled NLP-based extraction of HPO terms to be a sufficient replacement for manual extraction, providing evidence for the utility of the tiered filtering approach in a high-throughput environment. Overall, we illustrate a framework for learning from already genetically diagnosed cases to maximize the utility of NLP via filtering methods and describe a modular software architecture to implement our framework for research and clinical applications, which can be replicated across different health care systems. Implementation of this scalable automated approach has potential to significantly reduce the manual effort required to phenotype patients with complex diseases and increase the efficiency of molecular genetic diagnostic programs.

## Subjects and methods

### Study overview

**Figure 1.**
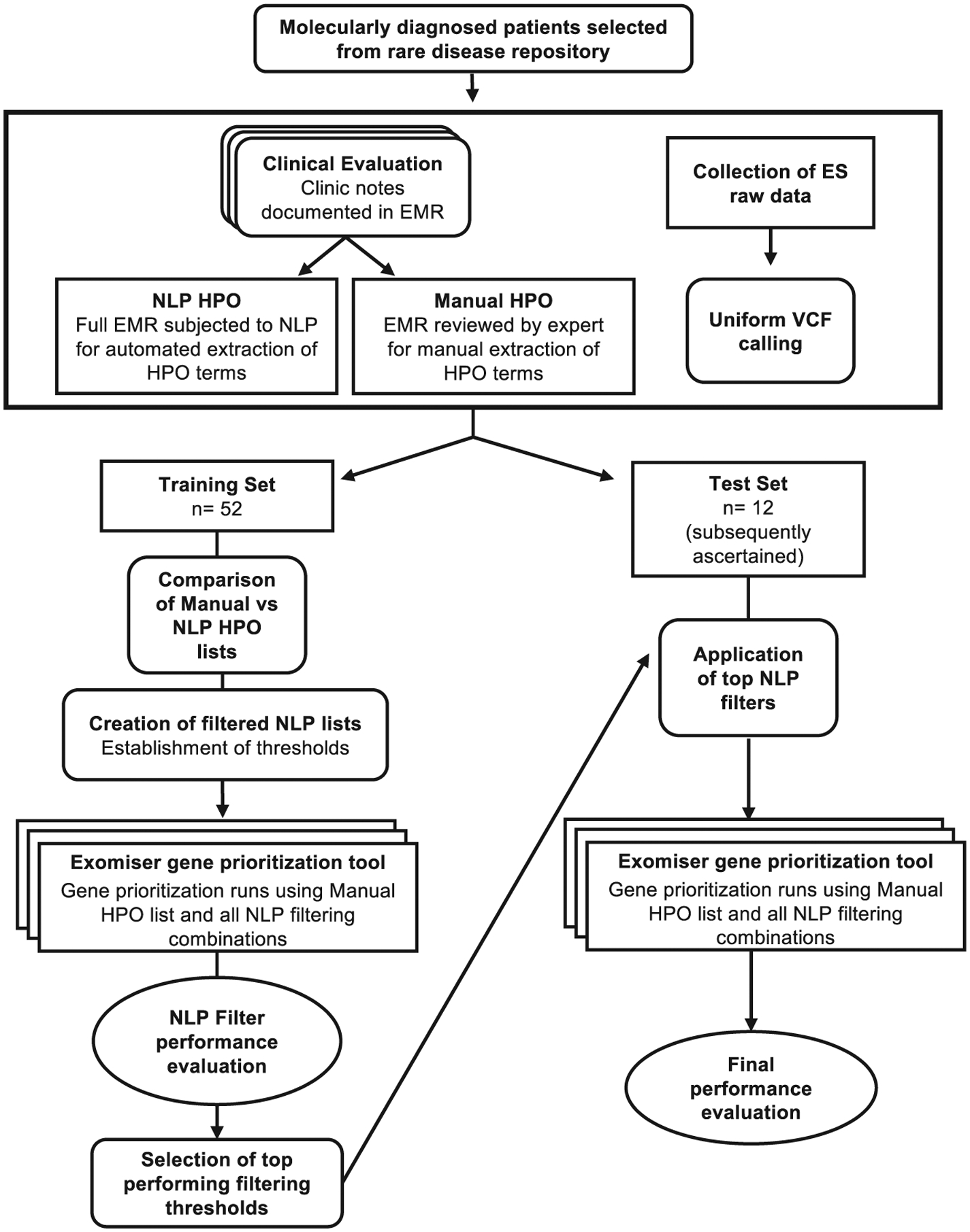
Study flow diagram Schematic of the overall study design and analysis plan. For each patient in a training set of 52 patients, we employed uniform processes to collect Human Phenotype Ontology (HPO) terms extracted by natural language processing (NLP), manually extracted HPO terms, and exome sequencing (ES) data in the form of variant call files (VCFs). Manually extracted HPO terms were compared to NLP-extracted HPO terms per patient in the training set with respect to (1) frequency of use, (2) HPO term depth within the ontology, and (3) diversity of phenotypic abnormality classes captured, confirming significant differences across all three dimensions. Next, we established thresholds per dimension that were used to create filtered lists of NLP terms per patient. Exomiser was run on each of the filtered NLP term lists (in addition to the manual and unfiltered NLP lists for comparison) per patient, and performance per filter was evaluated using metrics such as area under the receiver operating curve (AUC) and sensitivity. Top-performing NLP filters were combined into a tiered pipeline, which was finally applied to and evaluated on a subsequently ascertained set of 12 patients in the test set, whose data were collected using the same uniform processes described above.

The goal of this study is to provide a replicable framework to maximize the utility of NLP-based phenotype extraction from EHRs for use with gene prioritization algorithms and is motivated by the need to reduce the manual effort required to evaluate prioritized variants/genes. Our hypothesis is that a solution lies in filtering NLP-extracted terms to more closely resemble manually extracted terms. Figure 1 summarizes our study design and analysis plan. Subsequent sections describe the patient cohorts and data used, and the methods and results for comparing manual- versus NLP-extracted HPO lists, translating observed differences to a tiered NLP filtering approach and evaluating gene prioritization performance on a subsequently ascertained test set.

### Study subjects

All patients were ascertained through an existing rare disease gene discovery protocol of the Manton Center for Orphan Disease Research Gene Discovery Core at Boston Children’s Hospital (BCH), and all provided informed consent under the supervision of the hospital’s Institutional Review Board. Some were sequenced through the Children’s Rare Disease Cohort initiative,^16^ and data on a subset of these patients have been analyzed previously.^8,12^ The study subjects represent probands with a variety of clinical presentations, all with a genetically diagnosed rare monogenic etiology (Table S1). Patients’ ages for which most recent phenotypic data were available ranged from 0.04 to 24 years (mean = 7.69 years) (Table S2). All patients had at least one physician-authored outpatient record or inpatient consultation in the Boston Children’s Hospital EHR system and genomic data in the form of a variant call file (VCF) available from ES. Manual curations of HPO terms for all cases were carried out by two expert curators by reading patient medical records and identifying or applying HPO terms using the HPO lookup tool incorporated in PhenoTips.^17^ NLP extraction of HPO terms was performed by Clinithink’s patented Clinical Natural Language Processing (CNLP) engine, CLiX^16^ (see Supplemental subjects and methods). Clinical phenotypic data in the EHR were de-identified following extraction of HPO terms and related to de-identified genotypic data matched by study ID. All human studies described herein adhere to the principles set out in the Declaration of Helsinki, and every subject involved in this study provided informed consent in accordance with the ethical standards of the Boston Children’s Hospital Institutional Review Board.

### Phenotype data extraction

Patient records were stored in the Boston Children’s Hospital Cerner Electronic Health Record (CERNER EHR) database, which enables integrated storage of different types of medical records from health care providers, including outpatient and inpatient records, consultations, surgical notes, and imaging and procedure forms, as well as lab results.

The patients’ clinician-authored outpatient records or inpatient consultations in the EHR were used for both manual and Clinithink NLP curations. Scanned records, such as images from external health care institutions, were omitted.

Manual curations of HPO terms for all cases were carried out by two curators by reading patient medical records and identifying or applying HPO terms using the HPO lookup tool incorporated in PhenoTips.^2^ The curators were trained genetic research assistants with 2 to 3 years of experience, under the supervision of a certified and licensed Master’s degree level genetic counselor (C.A.G.), and with the oversight of a physician (P.B.A.) and a PhD molecular geneticist (A.H.B.). The curators were blinded to the genetic diagnosis of the patient. For each phenotype, the most precise term was picked depending on the definition of the HPO term. The curator was selective for terms potentially relevant to the patient’s overall clinical presentation and useful for diagnosis and omitted less-relevant terms such as a single fever or trauma.

NLP extraction of HPO terms from 462 different document types from CERNER EHR was performed by Clinithink’s patented CNLP engine, CLiX, using HPO Queryset v.11.2 (see Table S6 in Rockowitz et al.^16^).

Raw NLP-extracted terms included a number of different false positives in contrast to manual curations of medical records. The most significant source of false positives was physician’s notes regarding differential diagnoses containing unconfirmed disorders. As an example, a patient with transient infantile hypertriglyceridemia had false positives like hyperglycosemia and abnormal amino-acid metabolism generated from a differential diagnosis list in an outpatient medical record. Other false positives generated by NLP included medication-induced symptoms and signs, terms generated from patient/physician names, and the misinterpretation of common words as clinical symptoms. Based on manual review of 6 sets of medical records (data not shown), we estimated 21% of raw NLP-extracted terms to be false positives.

Data, available at initiation of this project, for a training set of 52 patients with a known causal diagnostic variant(s) were utilized to establish the filtering methodology described herein. A test set, comprised of 12 similar cases ascertained subsequently, was used to prospectively test the filtering infrastructure to ensure reproducibility and effectiveness across multiple groups (Table S2).

### NLP term features

We computed the following values per patient for a given set of NLP-extracted HPO terms: (1) mean frequency percentile, (2) mean depth, and (3) diversity.

Frequency percentile was calculated using the ranks of all HPO terms for a given patient based on term frequency; tied ranks were averaged. Depth was calculated as the distance of the shortest directed path from the root node in the HPO ontology to the respective term using an unweighted breadth-first search. Each term was assigned all unique phenotypic abnormality classes that its shortest paths passed (Table S3). We defined diversity as the number of unique phenotypic abnormality classes represented within a given set of HPO terms. We utilize the diversity and depth features as a proxy for term specificity in our analysis below (see Supplemental subjects and methods for additional details).

### Comparing distributions of NLP-extracted versus manually extracted terms

We split the NLP-extracted terms into two sets per patient in the training set: (1) those that were also identified by manual curation of EHR (“Both Manual and NLP”), and (2) those that were identified by NLP but not by manual curation of EHR (“NLP Only”).

The goal was to understand how best to filter the NLP-derived terms based on their features, with the assumption that there may be false positives among the terms that were not also identified manually. Therefore, we excluded from this analysis the group of terms that were identified manually but not by NLP. On average, 82% of all manually derived terms were also identified by NLP, indicating that the overlapping terms are a representative sample of all manually identified terms (Figure S1A). Extending the analysis to include ontologically related terms within two steps of each other revealed that only 3.5% of manually extracted terms had no closely related overlapping term in the NLP-derived set, and manual inspection did not identify any particular classes or characteristics of missed terms (Figure S1B).

Next, we computed values for the three-term set features (mean frequency percentile, mean depth, and diversity) for each of the two sets (“Both Manual and NLP” and “NLP Only”) per patient. We compared distributions of the two sets, each with 52 values for a given feature such as diversity, using a Wilcoxon’s signed-rank test, where the values were paired by patient. The distributions were considered significantly different if the p value was less than 0.01.

### Filtering NLP-derived terms

We set thresholds for frequency percentile, depth, and diversity equal to the 5^th^, 25^th^, 50^th^, 75^th^, and 95^th^ percentiles of the distributions of mean frequency percentile, mean depth, and diversity for the “Both Manual and NLP” sets of HPO terms. For a given patient and threshold per feature, the NLP-derived terms were filtered as follows:

Step 1: calculate frequency percentiles per term and remove all terms below a frequency percentile threshold
Step 2: remove all remaining terms with distances from the HPO root node below a depth threshold
Step 3: calculate mean frequency percentiles for terms grouped by phenotypic abnormality class (note that a term may contribute to multiple), sort abnormality classes by mean frequency percentile in descending order, and select terms belonging to the top N (inclusive) classes, where N is the diversity threshold.
Step 4: if the remaining number of terms is <5, then do not apply any filters.

The choice of filtering by diversity last was due to the impact of prior filtering on sorting the phenotypic abnormality classes by mean frequency percentile.

### Performance evaluation criteria

Performance was evaluated using results from the Exomiser variant prioritization tool.^15^ Exomiser output was evaluated using seven criteria: (1) the median gene score corresponding to the correctly identified variants, (2) the median rank of the genes containing the correctly identified variants, (3) the minimum number of ranked genes needed to identify all correct diagnostic pathogenic variants, (4) the area under the receiver operating characteristic curve (AUC) and the sensitivity for causal variants to be ranked within the top (5) 5 genes, (6) 10 genes, and (7) 20 genes. Exomiser outputs a variant score based on variant pathogenicity, a phenotype score based on semantic similarity, and a combined score that is a function of the variant and phenotype scores. Exomiser groups variants by gene, assigning each gene the score of its highest-scoring variant (or mean top 2 for compound heterozygotes); the gene score is used to rank the genes. The performance metrics were computed using the gene score and rank (see Supplemental subjects and methods for details).

### Ensemble algorithm

For each patient, we averaged the combined Exomiser scores per gene across all 294 combinations of NLP filtering parameters to calculate the ensemble scores. We used the mean combined Exomiser score to rank the genes. The ranks were then used to compute the expected average performance, as described above, of our NLP filtering methods as an ensemble. Note that an ensemble ranking can be determined using maximum votes or average ranking if the mean score is not a reasonable option for a different gene prioritization tool.

## Results

### Manual phenotyping results in better gene prioritization

We used seven criteria (see Subjects and methods) to compare the diagnostic impact of using manual phenotyping by an expert curator versus automated NLP extraction of phenotypes from the EHR. This comparison was done using Exomiser, a representative variant prioritization tool, on a training set of 52 diagnosed patients. All the genetic data processing parameters for Exomiser were held constant (details in Supplemental subjects and methods).

**Figure 2.**
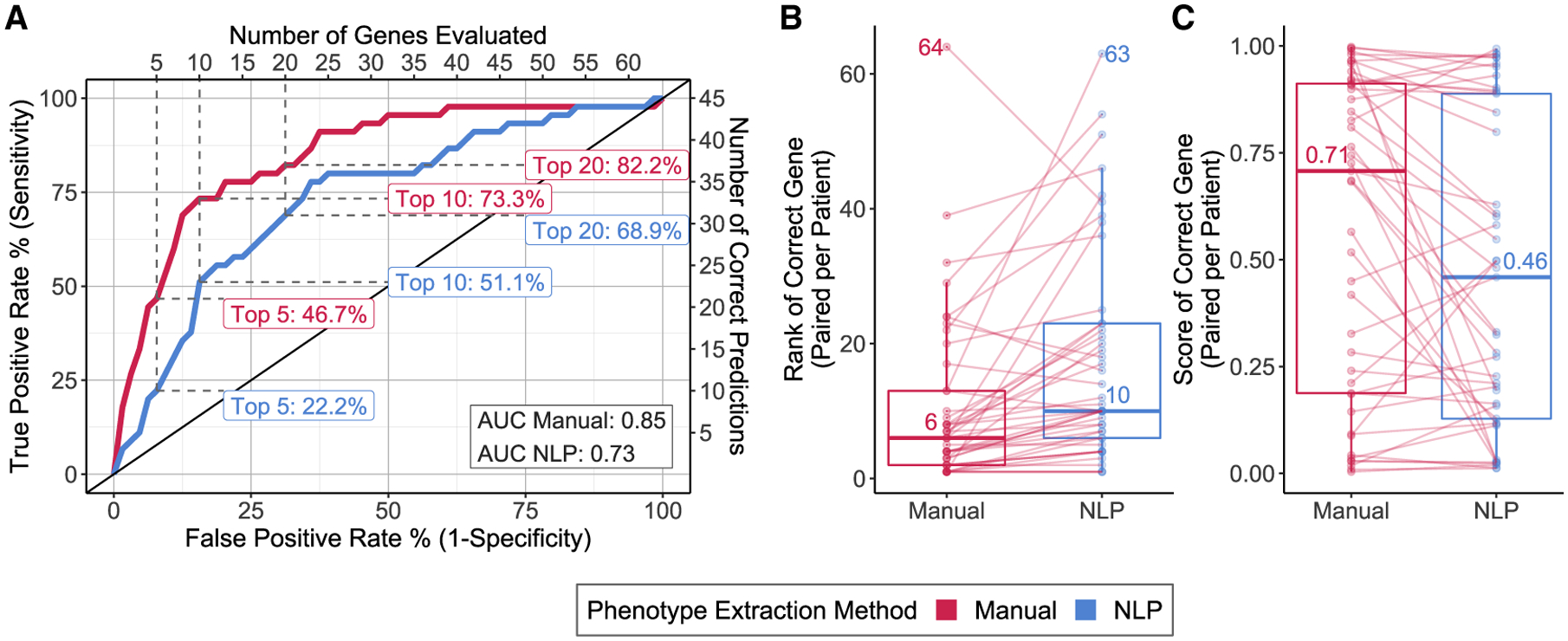
Performance of Exomiser using phenotypes extracted by manual curation versus natural language processing (NLP) among training set cases (A) Receiver operating characteristic curves with sensitivities noted for specificities corresponding to the top 5, 10, and 20 ranked genes, respectively. (B) Box and whiskers plots of distribution of the ranks of the correct genes. Each data point in a distribution corresponds to a specific patient, with lines connecting the ranks of each patient across the two phenotype extraction methods to indicate increase versus decrease in rank. The median and max (worst) ranks are also noted adjacent to the corresponding values in the distributions. (C) Box and whiskers plots of the distribution of the combined Exomiser scores for the correct gene per patient. Each data point in a distribution corresponds to a specific patient, with lines connecting the scores of each patient across the two phenotype extraction methods to indicate increase versus decrease in score. The median scores are noted adjacent to the median values in the distributions.

Exomiser reported the disease-causing variant in 45 of the 52 patients in the training set. The overall performance of Exomiser in correctly identifying the causal gene in 45 patients using manual phenotyping was better than NLP-based phenotyping, with an AUC of 0.85 versus 0.73, respectively (Figure 2A). The greatest difference in sensitivity was seen when considering only the top 5 ranked genes with manual phenotyping (46.7%) being more than twice as sensitive as the NLP-based approach (22.2%). The difference in sensitivity was reduced when considering a larger set of top 10 (73.3% manual versus 51.1% NLP) or top 20 genes (82.2% manual versus 68.9% NLP). Causal genes that were correctly identified when using manual phenotyping were ranked higher than when using NLP phenotyping, with a decrease of 4 in median rank (lower value of rank is better) and a corresponding 0.25 increase in median score; Exomiser scores range between 0 and 1 (Figures 2B and 2C.). However, the rank of the correct causal gene in 10 patients when using NLP-based phenotyping was the same or better than with manual phenotyping (Table S4).

To rule out that underlying characteristics of a patient’s genetic disorder impacted which phenotyping method led to better Exomiser performance, we compared distributions of (1) the type of genetic disorder, (2) pathogenicity status of the causal variant, and (3) the variant effect in the group of patients where manual phenotyping resulted in higher gene ranks than NLP-based phenotyping versus the group of patients where it did not. None of these characteristics were enriched in either of the two groups of patients, indicating that these factors did not influence the relative efficiencies of the manual and NLP-enabled approaches (Figure S2). Overall, gene ranking was more correlated with the phenotypic sub-score rather than the variant sub-score (Figures S3 and S4).

We next focused our efforts on post-extraction phenotypic data processing. An obvious difference was that the number of HPO terms extracted by NLP was higher (median number of terms = 340) than the corresponding manual extraction (median number of terms = 15), suggesting the potential for extraneous NLP-derived terms affecting the performance of Exomiser.

### Comparing features of NLP-extracted versus manually extracted HPO terms

We looked for differences in features of the HPO terms that were identified by NLP but not by the manual approach, hypothesizing possible enrichment of false-positive and non-specific terms. We suspected that correct terms, as verified by manual curation, are likely to be entered more often in the EHR (have a higher frequency) and that more specific terms have a higher significance in describing the phenotype of the disease. Therefore, we defined two features as proxies for specificity of a set of terms: mean depth and diversity. While depth captured the specificity of the description of a single term relative to its parents in the ontology structure, diversity captured the breadth of phenotypes by counting how many different phenotypic abnormality branches (classes) of the ontology were represented within a set of terms.

**Figure 3.**
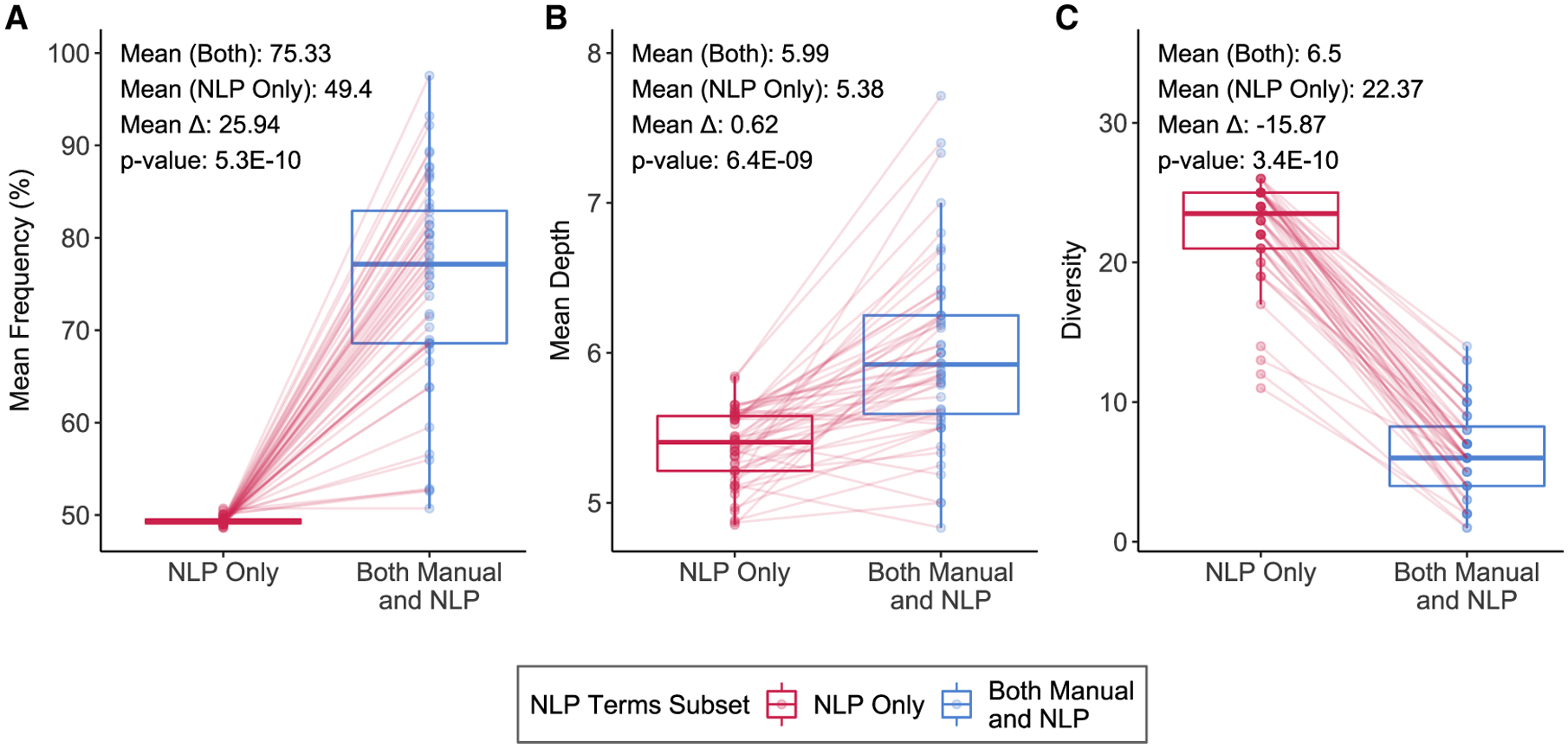
Comparing features of HPO terms identified by NLP alone versus terms identified by both manual- and NLP-based extraction Box and whiskers plots of (A) distribution of mean frequency percentiles of HPO terms, (B) distribution of mean depth of HPO terms, and (C) distribution of diversity of HPO terms. Each data point in a distribution corresponds to a specific patient in the training set, with lines connecting values of the respective summary feature per patient across the two NLP term subsets to indicate increase versus decrease in value. Mean values per distribition, the difference in means, and associated p-values, calculated using a Wilcoxon’s signed-rank test, are noted above each plot.

We compared the term frequency as a percentile, depth, and diversity per patient between (1) the set of terms that were identified using both approaches, and (2) the set of terms identified by NLP alone (Figure 3). The mean frequency percentile for terms identified by both approaches was consistently higher (grand mean 75%) than with NLP alone (grand mean 49%). In all patients, there were more HPO terms in the top half of most frequent terms (mean percentile > 50%) that were identified by both approaches. Terms per patient identified manually and by NLP were 0.62 levels deeper on average than terms identified by NLP alone for the same patient. The greatest difference between the pairs of term sets per patient was in diversity, where terms identified by both approaches represented an average of 6.5 different phenotypic abnormality classes versus 22.37 different phenotypic abnormality classes for NLP alone. There are 25 total unique phenotypic abnormality classes within HPO, suggesting that NLP-extracted terms spanned most of the breadth of the ontology, while human curation led to more targeted classes. The difference in distributions between the two sets of terms was significant, with Wilcoxon’s signed-rank test p values < 0.01 for all three features, frequency percentile (p value = 5.3E–10), mean depth (p value = 6.4E–9), and diversity (p value = 3.4E–10).

### Effect of NLP-extracted term filtering on gene prioritization performance

Given the above feature differences between the HPO term sets, we hypothesized that filtering NLP-extracted terms to more closely resemble terms that had also been identified manually may improve gene prioritization. Since filtering may adversely impact gene prioritization by removing true-positive terms as well, we varied the threshold per term set feature from tolerant to stringent (5–95 percentiles; see Subjects and methods) to filter the list of NLP-extracted terms per patient and evaluated the impact of filtering on Exomiser performance (Figure S5). We explored the 3D performance landscape for all possible combinations of seven different frequency thresholds (0%, 40%, 50%, 60%, 70%, 80%, 90%), six different minimum depth thresholds (0, 4, 5, 6, 7, 8), and seven different diversity thresholds (0, 2, 4, 6, 8, 10, 12) for a total of 294 filter parameter combinations applied to the NLP-extracted HPO terms. We ran Exomiser on the 52 patients in the training set using each of the 294 sets of filtered NLP-extracted HPO terms for a total of 15,288 Exomiser runs and measured performance using the aforementioned criteria (Figure S6; Table S5).

Top-performing filter combinations tended to have a high frequency percentile threshold between 70%–90%, a depth threshold of 6, and diversity thresholds of 6 or higher (Table 1; Table S3). These thresholds more closely resemble expected characteristics of NLP-extracted HPO terms that were also identified manually than NLP-only terms (Figure 3). In the subsequent sections, we refer to an NLP filter combination by its frequency/depth/diversity thresholds (e.g., frequency percentile threshold of 80%, depth threshold of 6 levels, and diversity threshold of 6 abnormality classes is designated as 80/6/6).

NLP filter combinations 80/6/6 and 90/6/6 appeared to be most promising based on our retrospective analysis of the training set (Table 1; Table S6). Combination 80/6/6 had the best sensitivity for causal variants to be ranked within the top 5 genes (51.1%, which was superior to manual’s 46.7%) and median rank (5, which was superior to manual’s 6), while 90/6/6 had the best sensitivity for inclusion in the top 20 genes (91.1%, which was superior to manual’s 82.2%). Finally, we found that the average NLP filter (“ensemble”) was a better choice than not applying any filter (unfiltered NLP) across all performance metrics and was superior to manual phenotyping in terms of the number of genes needed (49 versus 64 genes) to identify causal genes for all 45 patients and consequently the sensitivity for inclusion in the top 50 genes (100% versus 97.8%) (Figure S7).

### Optimizing diagnostic efficiency through a tiered approach to filtering NLP-extracted HPO terms

Our primary motivation is to minimize the number of variants for a clinician or expert to manually evaluate. We constructed a tiered approach to prioritizing genes using NLP-based phenotype extraction that incrementally increases the number of genes/variants to consider as the true diagnosis (Figure S8). Based on the previous analysis (Table 1), we ran gene prioritization with NLP terms filtered using 80/6/6 thresholds as our first tier (step 1) where we examined only the variants within the first 5 ranked genes, which reflects our ideal and most efficient outcome. If none of those variants were considered for further evaluation, the second tier (step 2) was to run gene/variant prioritization with NLP terms filtered using 90/6/6 and evaluating variants within the top 20 genes. If none of the variants in the top 20 genes were considered for further evaluation, we ran all combinations of NLP filters and ranked the genes by the average score (ensemble, step 3). Here, we would consider variants within the top 50 genes, representing the practical limit at which we assessed the case as having causal variants that were either not identifiable or represented in the data or that the NLP may have not captured relevant phenotypes. Finally, if none of the top 50 genes were considered for follow-up, the last tier would be to manually review the medical record and revert to evaluating all variants using that gold standard for HPO curation.

### Applying an NLP-extracted phenotype filtering strategy on prospective cases

As expected, optimizing our approach in this way led to improved performance in the training set. To evaluate the utility of this approach in the real world, we applied this NLP filtering method on 12 additional genetically diagnosed patients, referred to as the test set. The 12 cases were subsequently ascertained using the same criteria and data processing workflows as those in the training set (Figure 1). By following the tiered filtration protocol, Exomiser was able to detect the correct causal gene in all 12 cases. Of the 12 cases, the correct causal variant for one patient was identified in step 1, for seven additional patients in step 2, for three additional patients in step 3, and for the one remaining patient in step 4 (Table 2). Overall, NLP-based extraction of HPO terms was a sufficient replacement for manual extraction in 11 out of 12 (92%) cases. The sensitivity within the top 50 genes when using manual phenotyping was also 92% (Figure S9).

We also compared the above tiered pipeline results with results using the unfiltered NLP-extracted HPO terms. Compared to 92% sensitivity in the top 50 genes when using our tiered pipeline, unfiltered NLP phenotyping was a sufficient replacement for manual phenotyping in only 75% of prospective cases (Table 2). In 9 of the 12 cases (75%), the gene with the correct variant was ranked higher with an applied filter (including the ensemble) than without any filters (Table S7); the gene ranks were tied in the remaining three cases. In one case (MAN_0842) NLP filtering led to a 23-rank improvement, with the correct variant being ranked in the top 10 (ranked 6) genes as opposed to falling out of the top 20 genes (ranked 29) without any NLP filtering. In two other cases (MAN_1845 and MAN_0805), the correct variant would have been ranked out of the top 50 genes if the NLP terms were not filtered. These results indicate that beneficial NLP term filters can be applied to new patients to improve gene prioritization results. While out of the scope of this work, the diagnostic sensitivities achieved with NLP filtering could be further improved by optimizing Exomiser parameters or using other gene prioritization tools.

### Enrichment of manually identified phenotypes after filtering NLP-extracted terms

Our NLP filtering approach was designed to select HPO terms that more closely resemble manual terms with respect to frequency, depth, and diversity. In doing so, we enriched for phenotypes that manual curators selected to characterize each patient’s disease (Figure S10A). On average, 4% of unfiltered NLP terms were also identified manually, which coincides with the proportion of the average number of manual terms (14.6) versus NLP terms (355.4). However, after applying the filters in steps 1 and 2 of our tiered pipeline, the average percentage of manual terms in the remaining set of NLP terms increased to 17% (of an average 19.9 terms) and 23% (of an average 12.7 terms), respectively. Moreover, of the NLP terms that did not exactly match a manually identified term, the percentage of closely related terms (defined as having an undirected path length ≤ 2 in the ontology) increased from 13% in the unfiltered NLP lists to 32% and 34% on average after applying step 1 and 2 NLP filters, respectively (Figure S10B). Similarly, the average percentage of unmatched NLP terms that belonged to the phenotypic abnormality classes represented in the manual terms also increased from 57% in the unfiltered NLP lists to 80% and 85% after the step 1 and 2 filters, respectively (Figure S10C). The increased proportion of manually extracted and related terms in the filtered NLP lists indicates that our approach achieved the desired reduction of extraneous terms, a better characterization of the disease phenotype, and the consequent improvement in gene prioritization performance.

### Overview of modular software architecture

Our tiered pipeline, running one patient or many patients at a time, requires batch processing of multiple VCF-HPO file combinations, especially when running the ensemble algorithm in step 3. This approach is intended to be applicable to many different diagnostic settings and computational environments; therefore, it was imperative that we implemented a replicable and scalable framework that could batch process many VCF-HPO combinations in parallel. To achieve this, we implemented a batch-processing system that ran Exomiser within a docker container on the Amazon Web Services (AWS) cloud with all input data and results stored on AWS simple storage service (S3) and computed using their elastic compute cloud (EC2) (see Figure S11 and Supplemental subjects and methods).

## Discussion

The patient cohort employed for this study represents the most challenging types of cases encountered in a clinical environment. Subjects were enrolled into the Manton Center Gene Discovery Core after extensive clinical evaluation and diagnostic sequencing, including gene panel testing and/or ES that were deemed negative. While ES is increasingly being used as a first-tier diagnostic tool, the infrastructure and funding needed for reanalysis of ES-negative cases is lacking in most clinical and research settings. Furthermore, the expertise and time needed to manually phenotype individuals who often undergo extensive evaluations over long periods of time with complex and large medical charts can be challenging. The use of NLP to extract phenotypic information can overcome this issue. However, a drawback of this automated approach is the relatively high numbers of false-positive and non-specific repetitive terms compared with results of more laborious manual curation. In this paper, we describe the creation and implementation of an automated, reproducible filtering technique that can be applied across health care systems and computing environments to enable the utilization of NLP-extracted terms as an effective substitute for manually extracted HPO terms.

We scanned a three-dimensional feature space of NLP-derived HPO terms—each feature displaying significant variability between NLP-extracted versus manually extracted terms—for filter parameter combinations that optimized gene/variant prioritization. We incorporated the optimal parameter combinations within a tiered filter pipeline that resulted in an outcome comparable to or better than manually curated terms when applied to an independent test set. While previous work^12^ has evaluated similar features such as term frequency and proxies for term specificity such as information content, this is the first effort, to the best of our knowledge, to consider combinations of parameters. Furthermore, our approach does not rely on third-party datasets such as STRIDE,^18^ facilitating integration with different NLP extractors. However, future work that integrates more sophisticated measures of term specificity, such as information content and weighted paths, as well as ensembles of gene prioritization and NLP extraction algorithms, may improve our filtration approach. A continuing challenge with rare disease data analysis is the limited size of available patient datasets. We are encouraged by the consistency between the results in the training and test sets in terms of overall performance as well as patterns in the underlying metrics. Nevertheless, future studies ought to be expanded to larger datasets for learning filter parameters and out-of-sample testing in larger cohorts.

We considered that other institutions may choose to use different computing environments and aim to modularize their software architecture with substitutable components (Figure S11). The key modules in our architecture are (1) the NLP engine for HPO term extraction, (2) the gene prioritizer, and (3) the batch-processing engine, for which we used Clinithink’s CLiX Focus, Exomiser, and parallel processing using Ray^19^ on a single AWS EC2 instance, respectively. Multiple options are available for each of these modules and can readily replace our choices (see Supplemental information). We expect that cohorts, EHRs, and consequently optimal combinations of filter parameters will vary by applications and institutions. However, the framework of learning filter parameters from a training set of approximately 50 patients, where HPO terms are extracted manually as well as using NLP, is generally applicable.

Within the context of the Manton Center’s Gene Discovery Core, much greater effort is given to manual curation and selection of HPO terms than is normally available in a clinical diagnostic setting. Indeed, the depth and quality of phenotypic data typically available to clinical DNA diagnostic services are notoriously poor, leading to missed diagnoses. The rigorous use of appropriately filtered NLP-based phenotyping has the potential to significantly improve the efficiency of the diagnostic process by limiting the numbers of genes and variants that analysts and clinicians will need to consider before reviewing what ultimately may be determined to represent the causative genetic variant for patients with rare genetic diseases. Such an approach should have similar benefits in both a routine first-pass clinical diagnostic setting, as well as for clinical and research-based reanalysis programs where automated updating from more recently acquired clinical information may provide critical new data to enable a diagnosis.

## Supplementary Material

Table S1

Table S3

Main supplement

Table S8

Table S5

## Figures and Tables

**Table 1.** Parameter combinations for the top-performing natural language processing (NLP) filters

	Best NLP Filter		
Filtering criteria for top combinations	Frequency (%)	Depth	Diversity
AUC	90	6	6
Median rank	80	6	6
Median score	90	0	12
Genes needed	60	4	10
Sensitivity top 5	80	6	6
Sensitivity top 10	90	6	4
Sensitivity top 20	90	6	6
Median (median absolute deviation)	**90 (0)**	**6 (0)**	**6 (0)**

**Table 2.** Sensitivity in prospectively analyzed test set cases comparing NLP filters from the pipeline versus using unfiltered NLP

Pipeline step	Using pipeline NLP filters (n, cumulative %)	Using unfiltered NLP (n, cumulative %)
Step 1: top 5 genes (pipeline uses 80/6/6 filter)	1 (9.09)	1 (9.09)
Step 2: top 20 genes (pipeline uses 90/6/6 filter)	8 (66.67)	7 (58.33)
Step 3: top 50 genes (pipeline uses filter ensemble)	11 (91.67)	9 (75.00)
Step 4: all genes (pipeline uses manual phenotyping)	12 (100)	12 (100)

## Data Availability

Variant interpretations for causal variants are deposited in ClinVar. Additional data are available upon request from qualified investigators. Code is available at https://github.com/alxndgb/pheno_manuscript_Manton_ALXN.
