# Supplementary material for: A data-driven architecture using natural language processing to improve phenotyping efficiency and accelerate genetic diagnoses of rare disorders
Source: HGG Adv. 2021 May 11;2(3):100035. doi: 10.1016/j.xhgg.2021.100035 (PMC8432593; doi:10.1016/j.xhgg.2021.100035)
Supplement: Document S1. Supplemental subjects and methods, Figures S1–S12, and Tables S2, S4, S6, S7, and S9–S11 [file mmc1.pdf]

HGGA, Volume *2*

Supplemental information

A data-driven architecture using natural language processing to improve phenotyping efficiency and accelerate genetic diagnoses of rare disorders

Jignesh R. Parikh, Casie A. Genetti, Asli Aykanat, Catherine A. Brownstein, Klaus Schmitz-Abe, Morgan Danowski, Andrew Quitadomo, Jill A. Madden, Calum Yacoubian, Richard Gain, Tessa Williams, Mary Meskell, Andrew Brown, Alison Frith, Shira Rockowitz, Piotr Sliz, Pankaj B. Agrawal, Thomas Defay, Paul McDonagh, John Reynders, Sebastien Lefebvre, and Alan H. Beggs

SUPPLEMENTAL METHODS

Study population ... 3
Sequencing data ... 3
HPO description ... 3
Clinithink CliX overview ... 4
NLP term features ... 7
Gene prioritization ... 7
Computing infrastructure ... 8
Performance evaluation criteria ... 8
ROC calculation example ... 9
Issues with automated gene/variant prioritization ... 9
Effect of filtering based on each feature separately ... 10
Optimal combination of parameters ... 10
Overview of modular software architecture ... 11
Cromwell implementation challenges ... 12

SUPPLEMENTAL REFERENCES ... 13

SUPPLEMENTAL FIGURES

Figure S1 ... 15
Figure S2 ... 16
Figure S3 ... 17
Figure S4 ... 18
Figure S5 ... 19
Figure S6 ... 20
Figure S7 ... 22
Figure S8 ... 23
Figure S9 ... 24
Figure S10 ... 25
Figure S11 ... 26
Figure S12 ... 27

SUPPLEMENTAL TABLES

Table S1 ... 28
Table S2 ... 29
Table S3 ... 30
Table S4 ... 31
Table S5 ... 32
Table S6 ... 33
Table S7 ... 34
Table S8 ... 35
Table S9 ... 36
Table S10 ... 37
Table S11 ... 38

SUPPLEMENTAL METHODS

Study population

The patient cohort employed for this study represents the most challenging cases encountered in a clinical environment, where the readily diagnosable cases of genetic disease have already been solved through targeted testing, thus not requiring further investigation as described here. Typical patients referred to The Manton Center have undergone detailed phenotyping by geneticists and other clinical domain experts as well as had targeted gene or panel testing or clinical ES that was negative or inconclusive leading to reanalysis of their sequenced data[1]. Aside from these subjects having rare or atypical clinical presentations, salient or defining clinical features may be obscured by the use of very general descriptive terms such as "developmental delay", "hypotonia", etc., making detailed review of the clinical record critical to accurate and effective phenotyping. As many disease-gene relationships have only recently been identified, the reported phenotypic spectrums of these conditions are based on small numbers of cases and a number of patients in this cohort represented phenotypic expansions, making accurate and comprehensive phenotyping ever more critical to identify overlapping features with reported clinical presentations.

Sequencing data

The source of ES was paired-end short read Illumina-based sequencing derived from either a clinical diagnostic laboratory or research-based sequencing, with a date of sequencing ranging from 2012 to 2020. Raw sequencing data in the form of BAM or FASTq files were reprocessed via a single pipeline, VeXP, described previously[1]. In order to examine the impact of phenotypic information on making the diagnosis, we excluded trio or other family segregation data and analyzed ES results from probands only to avoid the confounding effects of de novo mutations in sporadic cases, identity by descent in consanguineous cases, etc.

HPO description

Human Phenotype Ontology (HPO) is formed of more than 13,000 thousand terms that describe the individual symptoms and signs observed in human diseases. HPO terms provide a standard and organized nomenclature for clinicians and researchers interested in studying phenotype driven data. These terms are determined through the use of different medical resources including Orphanet, DECIPER and OMIM. The HPO project can be accessed through http://www.human-phenotype-ontology.org.

Each term in the HPO describes a specific clinical abnormality and corresponds to a unique 7-digit HPO identifier. For example, Seizure is an HPO term with the uniquid identifier HP: 0001250. Different synonyms of the same clinical abnormalities are combined under one term. In this specific case, epileptic seizure, seizure and epilepsy would all be under the umbrella term Seizure HP: 0001250.

HPO terms are connected to each other depending on their hierarchal relationship. The first parent terms are formed of the most generalized and nonspecific clinical abnormalities while the more distal branches include more specific descriptions. This specificity is achieved through different subontologies describing the inheritance patterns, onset, localization, course and frequency of clinical manifestations. The distal branches of HPO terms inherit their parent terms all the way up to the root.

For example, Abnormality of the nervous system, HP: 0000707 is the root term that encompasses a variety of different clinical abnormalities pertaining to the nervous system. Its distal branches include Seizure HP: 0001250 which itself has numerous subclasses depending on its localization, onset and characteristics. One of its most distal branches includes Bilateral tonic-clonic seizure with focal onset, HP: 0007334, which is a very specific. Both of these terms inherit their more proximal parent terms all the way up to the most general root.

Clinthink CliX overview

Clinithink's patented Clinical Natural Language Processing (CNLP) engine, CLiX, has been developed over more than a decade of R&D. CLiX uses a combination of a statistical, ontological and linguistic approaches that have been refined and optimized specifically for clinical narrative found in physician-authored documentation. As part of the optimization process, a large training set of almost 10 billion words was utilized. CLiX supports an advanced NLP pipeline that focuses on noun phrases as the highest value sentence component and typically runs over 30 separate processes as part of the pipeline on the input noun phrase. The technology is highly scalable and can execute the entire pipeline in milliseconds on an input sentence. This equates to being able to process over 1 million documents in an hour on approximately $10 worth of AWS resources.

CLiX leverages SNOMED CT extensively as its primary ontology, enabling the computational representation of nearly 2 billion possible expressions commonly found in clinical narrative. Synonyms, acronyms and misspellings have all been extensively modelled and large dictionaries containing these variants continue to be maintained by Clinithink as part of the solution.

In the CNLP document processing workflow, CLiX undertakes heading recognition, segmentation, normalization and tokenization. Documents are first split into "headings" and then the input data is broken down into "segments" (sentences) and "tokens" (individual words or punctuation sequences). Each segment is normalized by converting the input data characters to the standard Latin character set. Tokens are analyzed for spelling errors and are corrected using an algorithm within CLiX. After spelling correction, tokens are analyzed against the metadata files (phrase-set, abbreviations, acronyms and synsets). Based on the content contained in these metadata files and the context file, tokens are replaced according to the content of the metadata files.

Once pre-processing is complete, concept matching begins, CLiX matches tokens to pre-coordinated SNOMED concepts and assembles legitimate fully-modelled post-coordinated expressions based on SNOMED compositional grammar. Input data is then reviewed by CLiX to

identify any elements that may not already be represented in the encoded data, such as quantities, date, time or value information. The information model data is stored with the SNOMED CT concepts to which the data applies. Because CLiX is based on SNOMED-CT, it can be used effectively for projects for any disease area. The core SNOMED-CT content is managed through a quality-based approach using expert personal, standard guidelines and process, and collaboration with key stakeholders and SME groups (including Clinithink). This means that there is a both a broad and deep approach to expanding the terminology so that it can be utilized for any clinical use case. The CLiX Engine is also customizable to be able to interpret how language/dialect is written locally (acronyms, synonyms, abbreviations, etc.) as well as expandable based on our SNOMED extension. This is important because innovative drugs, new tests, and new therapies are constantly being introduced to improve clinical care and outcomes.

After the CLiX Engine has encoded all clinical content found in the unstructured text to post-coordinated SNOMED, a key and unique part of our solution is a query capability to extract the phenotypes for each patient. For CLiX focus (Clinithink's deep phenotyping solution) an HPO Queryset has been created to output the relevant HPO codes and concepts for each patient based on the underlying SNOMED expressions. Clinithink's HPO Queryset is not a mapping of SNOMED to HPO, but a thorough representation of the full HPO concept using one or more SNOMED expressions, with the output being the representative HPO code and concepts for consumption by downstream systems. The HPO Queryset version 11.2 was used for this project. The development of this Queryset involved the following process:

1. Synonyms identified as "layperson" were deferred and removed from the target. Synonyms that were clinical and therefore more likely to be used in a patient's record were prioritized and none of these were deferred.
2. Concepts that were created for the sole purpose of hierarchal grouping were deferred. These were concepts that begin with "Abnormal" or "Abnormality of" and were not phrases that a clinician would use to describe a clinical feature. Each of these had children concepts/phenotypes that were better defined and attributed.
3. The remaining phenotypes and synonyms were processed against CLiX to determine the encoding coverage. The list was reviewed automatically as part of proprietary Clinithink workflow tools and manually as needed by clinical terminologists.
4. Those that weren't encoded or didn't have all the phenotypes attributes fully represented were put into a production workflow and prioritized based on relationships to other rare disease ontologies (OMIM & Orphanet) and population prevalence taken from source files.
    a. Typical encoding & Queryset representation issues included:
        - missing adjectives/attributes
        - foreign characters
        - unusual punctuation
        - missing sub-types
        - ambiguous acronyms
    b. CLiX configuration or Queryset development typically involves:
        - Adding or modifying CNLP configuration rules

- Adding to the Clinithink SNOMED extension to fully model the concept
- Adding logic to join multiple SNOMED expressions together in order to fully represent a single HPO concept

The CLiX Engine encodes negation as 'Known Absent' in the post-coordinate SNOMED Expression. However, the HPO Queryset does not currently leverage 'Known Absent' encodings.

An important part of any CLiX project is identifying the source clinical narrative and making sure that those documents are loaded with the highest quality, fidelity and integrity. Part of this process is classifying the types of narrative in the EHR or other source systems based on:

a. Clinical significance and relevance
b. Data formatting (is the document truly narrative or just structured values that may not be properly or fully interpreted due to imbedded tables from template-based document types)
c. Identifying patient instruction documents or other similar types that may be generic, informational and not specific to the patient or their encounter.

As part of this project, BCH provided a list of all the document types in their CERNER EHR system for review by the project team; CLiX does not natively ingest PDFs. This initial review was done collaboratively from members of the BCH and Clinithink teams and resulted in a list of 462 document types being flagged for importing into CLiX. (see Supplemental Table 6 in Rockowitz *et al*, 2020[3] for full list)

Those document types for the project cohort were extracted by BCH into a SQL staging table. The data was reviewed as like any standard CLiX implementation project to see if there were any pre-loading data transformations/standardizations that needed to happen to improve data quality and eliminate data loading errors. The CLiX Engine reads and interprets clinical narrative like a human does, therefore formatting of the document is key to accurate encoding.

Narrative text may often go through several integration pipelines and be transformed from several formats. This can result in artifacts (HTML, RTF, XML, HL7, etc.) being unintentionally embedded within the document itself. This can also include document headers and footers that disrupt the narrative flow of a document. These need to be removed or transformed prior to import into CLiX.

In addition, templates are commonly used within all modern EHRs, and although they can improve the clinician's user experience, they can leave characters, spacing, tables or other data flaws within the documents that any NLP product (or human) will struggle with accurately interpreting. Parts of these are identified during the initial data assessment process while the data is still in the staging database table, while others are identified using the CLiX engine itself. This may include identify new headers that were not included in the standard CLiX configuration, headers that need to be ignored because the subsequent data is not relevant to the patient, or other configuration needed to process BCH data – regardless of the use case. In other words, this configuration is not for customizing the CLiX engine for content, as much as it

is tuning the configuration based on the formatting of the documents. The templates and documents that BCH uses in their EHR system are not the same as other hospitals – even if it is the same CERNER system. For this project, the clinical documents for each of the patients in the study cohort were imported into CLiX focus and processed against the HPO Queryset v11.2.

NLP term features

We computed the following values per patient for a given set of NLP-extracted HPO terms: 1) mean frequency percentile, 2) mean depth, and 3) diversity.

Frequency percentile was calculated using the ranks of all HPO terms for a given patient based on term frequency; tied ranks were averaged. Mean frequency percentile was computed as the arithmetic mean of the frequency percentiles of all HPO terms within a given set.

Depth and phenotypic abnormality classes were determined using the HPO structure. Depth was calculated as the distance of the shortest directed path from the root node in the HPO ontology to the respective term using an unweighted breadth-first search. Mean depth was computed as the arithmetic mean of the depth of all HPO terms within a given set.

A term may have multiple shortest paths from the root node, by definition, with the same shortest distance. A term was assigned all unique phenotypic abnormality classes that its shortest paths passed through (Table S3; a list of phenotypic abnormalities and details on each HPO term can be found at http://www.informatics.jax.org/vocab/hp_ontology/HP:0000118). Terms that were at the same hierarchy level as a phenotypic abnormality class node or above were not assigned any class. We defined diversity as the number of unique phenotypic abnormality classes represented within a given set of HPO terms. We utilized the diversity and depth features as a proxy for term specificity in our analysis.

Gene prioritization

The latest version of Exomiser[4] (version 12.1.0 with 2003 versions of both the hg19 genome and phenotype data) was downloaded from https://Exomiser.github.io. Exomiser takes an input VCF file and a list of HPO-encoded phenotypes and ranks potential disease-causing genes by combining variant pathogenicity with semantic similarity between the patient's phenotypes and phenotypes associated with a gene via gene-disease associations from Orphanet[5] and OMIM.[6,7] We used the default parameters except for maxFrequency in the frequencyFilter, which was set to 1%, the phenix prioritizer was used instead of the hiPhive prioritizer, and the output formats were limited to HTML and JSON. All analysis settings are as follows:

genomeAssembly: hg19
inheritanceModes:
    { AUTOSOMAL_DOMINANT: 0.1, AUTOSOMAL_RECESSIVE_HOM_ALT: 0.1, AUTOSOMAL_RECESSIVE_COMP_HET: 2.0, X_DOMINANT: 0.1, X_RECESSIVE_HOM_ALT: 0.1, X_RECESSIVE_COMP_HET: 2.0, MITOCHONDRIAL: 0.2 }
analysisMode: PASS_ONLY

```
frequencySources:
    [ THOUSAND_GENOMES, TOPMED, UK10K, ESP_AFRICAN_AMERICAN,
    ESP_EUROPEAN_AMERICAN, ESP_ALL, EXAC_AFRICAN_INC_AFRICAN_AMERICAN,
    EXAC_AMERICAN, EXAC_SOUTH_ASIAN, EXAC_EAST_ASIAN,EXAC_FINNISH,
    EXAC_NON_FINNISH_EUROPEAN, EXAC_OTHER, GNOMAD_E_AFR, GNOMAD_E_AMR,
    GNOMAD_E_EAS, GNOMAD_E_FIN, GNOMAD_E_NFE, GNOMAD_E_OTH,
    GNOMAD_E_SAS, GNOMAD_G_AFR, GNOMAD_G_AMR, GNOMAD_G_EAS,
    GNOMAD_G_FIN, GNOMAD_G_NFE, GNOMAD_G_OTH, GNOMAD_G_SAS ]
pathogenicitySources: [POLYPHEN, MUTATION_TASTER, SIFT]
steps: [
    variantEffectFilter: {
        remove: [
            FIVE_PRIME_UTR_EXON_VARIANT, FIVE_PRIME_UTR_INTRON_VARIANT,
            THREE_PRIME_UTR_EXON_VARIANT,
            THREE_PRIME_UTR_INTRON_VARIANT,
            NON_CODING_TRANSCRIPT_EXON_VARIANT,
            UPSTREAM_GENE_VARIANT, INTERGENIC_VARIANT,
            REGULATORY_REGION_VARIANT,
            CODING_TRANSCRIPT_INTRON_VARIANT,
            NON_CODING_TRANSCRIPT_INTRON_VARIANT,
            DOWNSTREAM_GENE_VARIANT
        ]
    },
    frequencyFilter: {maxFrequency: 1.0},
    pathogenicityFilter: {keepNonPathogenic: true},
    inheritanceFilter: {},
    omimPrioritiser: {},
    phenixPrioritiser: {}
]
```

Computing infrastructure

R (version 3.5.2) and Python (version 3.8) were used for all data processing, analysis, and visualization. The ontologyIndex (version 2.5) R package was used for parsing ontology files in OBO format and the igraph (version 1.2.4.1) R package was used for computing shortest paths. The ray[8] (version 0.9) python package was used for parallel processing on AWS EC2. Cromwell[9] server (version 50) was installed on AWS EC2 and configured to deploy tasks using AWS Batch.

Performance evaluation criteria

Exomiser was able to identify the correct causal variant in 45 out of 52 patients in the training set. We computed the median of the gene score corresponding to the correct causal variant, henceforth referred to as the causal gene, across the aforementioned 45 patients as the first performance evaluation criterion. We also noted the rank of the correct causal gene per patient. We computed the mean rank and the lowest rank across all 45 patients as two additional performance evaluation criteria. Next, we computed the AUC as a balance between

the number of causal genes identified correctly and the rank of those genes. We computed the receiver operating curve (ROC) by calculating the sensitivity or true positive rate (TPR) as the percent of causal genes (out of 45) correctly identified for varying gene rank thresholds; the false positive rate (FPR) was calculated as the rank threshold divided by the lowest possible rank (see below for example calculation). The AUC under the TPR vs FPR ROC curve was calculated using the trapezoidal rule. The AUC depends on the lowest possible rank amongst runs to be compared and can vary for the same run if a different set of runs are compared such that the lowest possible rank changes. Therefore, we only used the AUC as a relative measure between pipeline runs. In order to account for this, we also used the sensitivity at specific gene rank thresholds of 5, 10, and 20, considering them as practical limits on the number of genes to evaluate per patient, as additional performance evaluation criteria.

ROC calculation example

The receiver operating characteristic (ROC) curve plots the true positive rate (TPR) at varying thresholds of a false positive rate (FPR). In this work, we computed the TPR as the fraction of patients where the correct causal gene (1 per patient) was predicted and the FPR as the fraction of total genes needed to be evaluated to make all possible correct predictions*, i.e.* an incremental gene rank threshold divided by the lowest possible rank across all patients. The following table shows the example TPR and FPR calculations:

# Genes Evaluated	FPR Formula	FPR	# Correct Predictions	TPR Formula	TPR
1	1 / 64	1.56%	8	8 / 45	17.78%
2	2 / 64	3.13%	12	12 / 45	26.67%
3	3 / 64	4.69%	15	15 / 45	33.33%
4	4 / 64	6.25%	20	20 / 45	44.44%
…					
63	63 / 64	98.44%	44	44 / 45	97.78%
64 (worst ranking gene)	64 / 64	100.00%	45 (total number of patients)	45 / 45	100.00%

Issues with automated gene/variant prioritization

Exomiser was unable to identify the correct causal gene in 7 out of the 52 patients irrespective of the phenotype extraction method. There were several reasons why gene prioritization was insufficient in these cases. In one of the cases, the causal gene would have been identified but the causative variant did not pass the maximum minor allele frequency threshold of 0.1% in one of the reference populations, the European ancestry (EA) cohort from the NHLBI GO Exome Sequencing Project (ESP)[10] by a minute margin (0.109%). In previous versions of the Exomiser phenotype data (v1909; we used v2003, which was the latest available at the time of this work), the variant would have been captured because it passed a ClinVar-based[11] whitelist. Exomiser authors envision a bespoke white list that would augment or supplant their default whitelist. In this work, we did not alter most genomic data processing parameters and default data files in order to limit the variability from non-phenotypic data sources. While out of scope for this study, we recommend further exploration of genomic parameters to focus gene prioritization

of the appropriate set of variants given the characteristic of the patient population to be studied.

Effect of filtering based on each feature separately

We applied frequency percentile, depth, and diversity filters one at a time to understand the effect of each filter on Exomiser performance (Figure S5). Filtering of NLP terms by frequency percentile improved overall performance relative to Exomiser results using unfiltered NLP terms, with AUCs increasing with increased stringency (higher threshold). Filtering by depth also had a positive correlation with stringency on performance for AUC and sensitivity within the top 5 ranking genes. Varying the diversity threshold had the largest impact on overall performance with the AUC and median rank worsening the most with increased stringency (smaller diversity threshold). Filtering of NLP terms by frequency percentile improved overall performance relative to Exomiser results using NLP terms without any filtering (dashed blue line in Figure S5) with AUCs increasing with increased stringency (higher threshold). The positive correlation between frequency percentile threshold and performance was also true for the median score. Sensitivity when considering the top 5 and top 20 ranking genes also improved with frequency filtering, while the sensitivity within the top 10 ranking genes and median rank stayed about the same or worsened slightly than before filtering. Filtering by frequency percentile also led to fewer genes needed to correctly identify the causal gene in all 45 possible patients. Filtering by depth also had a positive correlation with stringency on performance for AUC and sensitivity within the top 5 ranking genes. However, unlike frequency filtering there was an improvement in median rank and sensitivity within the top 10 genes, but a lack of improvement in median score. The number of genes needed was also lower than without filtering in all cases except the most stringent depth threshold of 8 levels deep. Diversity had the largest change in overall performance with the AUC and median rank worsening the most with increased stringency (smaller diversity threshold). The diversity threshold when applied independently of depth or frequency percentile likely led to the removal of important (high frequency) and specific (high depth) HPO terms, which did not belong to the phenotypic abnormality classes that were merely on average more frequently represented in the EHR. Given the complementary behavior in performance metrics between the three filter criteria, we next explored all possible combinations of frequency, depth, and diversity thresholds.

Optimal combination of parameters

In order to develop an approach to maximize the utility of NLP and determine best practices for filtering NLP outputs to bring gene prioritization performance as close to that following expert curation as possible, we explored the performance landscape for all possible combinations of NLP filters. We applied all combinations of 7 different frequency filters (0%, 40%, 50%, 60%, 70%, 80%, 90%), 6 different minimum depth filters (0, 4, 5, 6, 7, 8), and 7 different diversity filters (0, 2, 4, 6, 8, 10, 12) for a total of 294 (7 x 6 x 7) sets of filter parameter combinations on the NLP-extracted HPO terms for the 52 patients with known diagnosis. We ran Exomiser on the 52 patients in the training set using each of the 294 sets of filtered NLP-extracted HPO terms for a total of 15,288 Exomiser runs, and measured performance using 7 different performance criteria described above (Figure S6, Table S5). As the filters become more stringent there are

more patients where the filter combination left too few HPO terms (Table S8). However, 70% of filter combinations (208 out of 293; note 1 is the unfiltered NLP list) reduced the number of HPO terms to more than 5 terms in over 80% of patients (42 out of 52 patients).

Next, we explored the specific best NLP filter option per patient. The promise of knowing the best NLP filter combination for a given patient is that 43/45 (95.6%) of the patients would not need time consuming manual term extraction if that optimal filter combination were known *a priori* (Table S9). In order to decipher the relationship between optimal filter combinations and patients, we clustered the specific best NLP filter option per patient (Figure S12) to identify patterns of patients and filter parameters. We did not observe clear clusters of patients or identify major patterns in filter parameter combinations except for an anticorrelation between frequency percentile and depth filter thresholds where two distinct clusters benefited from either one of the two filters but not both (Table S10).

With sufficiently large sample sizes we may eventually be able to decipher which NLP filters would work best for which patients. However, until then we may still be able to provide a benefit with overall best performing NLP filters (Table S6).

To assess if these 3 NLP filter options would provide a benefit, we compared the rank of the correct gene after NLP filtering to their ranks when using manually extracted and the unfiltered NLP phenotype lists (Table S11). We split the patients into two groups based on the preferred phenotype extraction method if filtering were not an option (Table S4). Ties in rank were broken based on the following preference (Combination Filters [80/6/6 or 90/6/6] > Ensemble > Unfiltered NLP > Manual). 64.4% of patients (29 out of 45) would have at least as good a rank with an NLP filter applied as manual phenotyping. The majority (54.29%) of the patients (19 out of 35) that had a better rank with manual phenotyping than unfiltered NLP-based phenotyping would now have at least as good a rank with an NLP filter. Most of the patients that benefit from an NLP filter see the benefit with one of the specific NLP combination filters over the ensemble. Note that there were zero patients that benefitted from the unfiltered NLP over the filtered options. Combinations 80/6/6 and 90/6/6 would be the preferred choice of NLP filters for about the same number of patients (11 vs 13 respectively) with the correct gene for 4 patients being ranked the same with either filter (Table S11).

Overview of modular software architecture

Our tiered pipeline, run on one patient or many patients at a time, requires batch processing of multiple VCF-HPO file combinations, especially when running the ensemble algorithm in step 3. In one of our most extreme recent analyses, we needed to run 294 filter combinations on 110 patients. A single variant prioritization run, including data management and cleanup, requires approximately 7 minutes. The 32,340 (294 x 110) runs would have taken 157 days to complete if run serially. We intend this approach to be applicable to many different hospitals and computational environments, therefore, it was imperative that we implemented a replicable and scalable framework that could batch process many VCF-HPO combinations in parallel. We implemented a batch processing system in the cloud using Amazon Web Services (AWS) with all input data and results stored on AWS simple storage service (S3) and compute using their

elastic compute cloud (EC2) (Figure S11). The gene/variant prioritizer (Exomiser in our case) was containerized using docker[12] and stored in Docker Hub[13]. Containerization, i.e. encapsulating the repetitively accessed application, enables portability to almost any scalable computing infrastructure. In the simplest deployment, we ran batches of 65 VCF-HPO combinations that called the containerized gene prioritizer on a single memory-optimized EC2 instance that had 512 Gb of memory. The 32,340 runs completed in approximately 2 days and cost approximately $2 per patient and under $250 overall. We also implemented the parallel processing pipeline using Cromwell[9], developed by the Broad Institute, and AWS Batch, but replaced it due to error handling issues (see below). The runtime and costs could have been further reduced by using AWS Batch with spot instances.

We were mindful that other institutions may choose to use different computing environments and aim to modularize the software architecture with substitutable components (Figure S11). The key modules in our architecture are 1) the NLP engine for HPO term extraction, 2) the gene prioritizer, and 3) the batch processing engine, for which we used Clinithink's CLiX Focus, Exomiser, and parallel processing using Ray[8] on a single AWS EC2 instance respectively. NLP engine options outside of Clinthink include CLAMP[14], ClinPhen[15], and cTakes[16]. Exomiser may be substituted with MOON from Diploid[17,18], Extasy[19], Phen-Gen[20], and MutationDistiller[21] and others. As described above, we initially used Cromwell as our batch processing engine, but later replaced it with a custom script running on a single AWS EC2 instance. We intend to further increase efficiency and decrease costs by deploying our parallel processing pipeline on AWS Batch in the future. Other popular options for orchestrating parallel tasks and managing workflows are Luigi[22] and Apache Airflow[23]. Most of the options described above can be implemented on cloud platforms other than AWS as well as on premises.

Cromwell implementation challenges

We initially implemented the parallel processing pipeline using Cromwell[16], developed by the Broad Institute, and AWS Batch. One challenge with parallel processing pipelines that utilize data stored on AWS S3 is handling errors introduced by concurrent file access. These errors can be handled within custom scripts, as we did when implementing the aforementioned single instance-based pipeline. In Cromwell, we set the maxRetries runtime attribute to 3, which reduced the number of pipeline crashes due to S3 read errors. We found the principal advantages of Cromwell to be the reduction in programming required for orchestrating parallel jobs and the availability of an application programming interface (API) for submitting and monitoring jobs. While we did not optimize for cost savings, we found the overall compute cost and runtime to be higher when using Cromwell and that the handling of errors for our largest job was too onerous, and thus used a single on-demand instance for our batch processing pipeline.

SUPPLEMENTAL REFERENCES

1. Schmitz-Abe, K., Li, Q., Rosen, S.M., Nori, N., Madden, J.A.J.A., Genetti, C.A.C.A., Wojcik, M.H.M.H., Ponnaluri, S., Gubbels, C.S.C.S., Picker, J.D.J.D., et al. (2019). Unique bioinformatic approach and comprehensive reanalysis improve diagnostic yield of clinical exomes. Eur. J. Hum. Genet. *27*, 1398–1405.
2. Girdea, M., Dumitriu, S., Fiume, M., Bowdin, S., Boycott, K.M., Chénier, S., Chitayat, D., Faghfoury, H., Meyn, M.S., Ray, P.N., et al. (2013). PhenoTips: Patient phenotyping software for clinical and research use. Hum. Mutat. *34*, 1057–1065.
3. Rockowitz, S., LeCompte, N., Carmack, M., Quitadamo, A., Wang, L., Park, M., Knight, D., Sexton, E., Smith, L., Sheidley, B., et al. (2020). Children's rare disease cohorts: an integrative research and clinical genomics initiative. Npj Genomic Med. *5*, 1–12.
4. Smedley, D., Jacobsen, J.O.B., Jäger, M., Köhler, S., Holtgrewe, M., Schubach, M., Siragusa, E., Zemojtel, T., Buske, O.J., Washington, N.L., et al. (2015). Next-generation diagnostics and disease-gene discovery with the Exomiser. Nat. Protoc. *10*, 2004–2015.
5. Rath, A., Olry, A., Dhombres, F., Brandt, M.M., Urbero, B., and Ayme, S. (2012). Representation of rare diseases in health information systems: The orphanet approach to serve a wide range of end users. Hum. Mutat. *33*, 803–808.
6. Zemojtel, T., Köhler, S., Mackenroth, L., Jäger, M., Hecht, J., Krawitz, P., Graul-Neumann, L., Doelken, S., Ehmke, N., Spielmann, M., et al. (2014). Effective diagnosis of genetic disease by computational phenotype analysis of the disease-associated genome. Sci. Transl. Med. *6*, 252ra123-252ra123.
7. Amberger, J., Bocchini, C., and Hamosh, A. (2011). A new face and new challenges for Online Mendelian Inheritance in Man (OMIM®). Hum. Mutat. *32*, 564–567.
8. Ray – Fast and Simple Distributed Computing. https://ray.io/
9. Home - Cromwell. https://cromwell.readthedocs.io/en/stable/
10. Tennessen, J.A., Bigham, A.W., O'Connor, T.D., Fu, W., Kenny, E.E., Gravel, S., McGee, S., Do, R., Liu, X., Jun, G., et al. (2012). Evolution and functional impact of rare coding variation from deep sequencing of human exomes. Science (80-.). *336*, 64–69.
11. Harrison, S.M., Riggs, E.R., Maglott, D.R., Lee, J.M., Azzariti, D.R., Niehaus, A., Ramos, E.M., Martin, C.L., Landrum, M.J., and Rehm, H.L. (2016). Using ClinVar as a resource to support variant interpretation. Curr. Protoc. Hum. Genet. *2016*, 8.16.1-8.16.23.
12. Empowering App Development for Developers | Docker. https://www.docker.com/
13. Docker Hub. https://hub.docker.com/
14. Soysal, E., Wang, J., Jiang, M., Wu, Y., Pakhomov, S., Liu, H., and Xu, H. (2018). CLAMP - a toolkit for efficiently building customized clinical natural language processing pipelines. J. Am. Med. Informatics Assoc. *25*, 331–336.
15. Deisseroth, C.A., Birgmeier, J., Bodle, E.E., Kohler, J.N., Matalon, D.R., Nazarenko, Y., Genetti, C.A., Brownstein, C.A., Schmitz-Abe, K., Schoch, K., et al. (2019). ClinPhen extracts and prioritizes patient phenotypes directly from medical records to expedite genetic disease diagnosis. Genet. Med. *21*, 1585–1593.
16. Savova, G.K., Masanz, J.J., Ogren, P. V., Zheng, J., Sohn, S., Kipper-Schuler, K.C., and Chute, C.G. (2010). Mayo clinical Text Analysis and Knowledge Extraction System (cTAKES):

Architecture, component evaluation and applications. J. Am. Med. Informatics Assoc. *17*, 507–513.
17. Diploid - Diagnosing Rare Diseases. http://www.diploid.com/moon
18. Clark, M.M., Hildreth, A., Batalov, S., Ding, Y., Chowdhury, S., Watkins, K., Ellsworth, K., Camp, B., Kint, C.I., Yacoubian, C., et al. (2019). Diagnosis of genetic diseases in seriously ill children by rapid whole-genome sequencing and automated phenotyping and interpretation. Sci. Transl. Med. *11*,
19. Sifrim, A., Popovic, D., Tranchevent, L.C., Ardeshirdavani, A., Sakai, R., Konings, P., Vermeesch, J.R., Aerts, J., De Moor, B., and Moreau, Y. (2013). EXtasy: Variant prioritization by genomic data fusion. Nat. Methods *10*, 1083–1086.
20. Javed, A., Agrawal, S., and Ng, P.C. (2014). Phen-gen: Combining phenotype and genotype to analyze rare disorders. Nat. Methods *11*, 935–937.
21. Hombach, D., Schuelke, M., Knierim, E., Ehmke, N., Schwarz, J.M., Fischer-Zirnsak, B., and Seelow, D. (2019). MutationDistiller: User-Driven Identification of Pathogenic DNA Variants. Nucleic Acids Res. *47*, W114–W120.
22. Luigi. https://luigi.readthedocs.io/en/stable/
23. Apache Airflow. https://airflow.apache.org/
24. Best, D.J., and Roberts, D.E. (1975). Algorithm AS 89: The Upper Tail Probabilities of Spearman’s Rho. Appl. Stat. *24*, 377.

SUPPLEMENTAL FIGURES

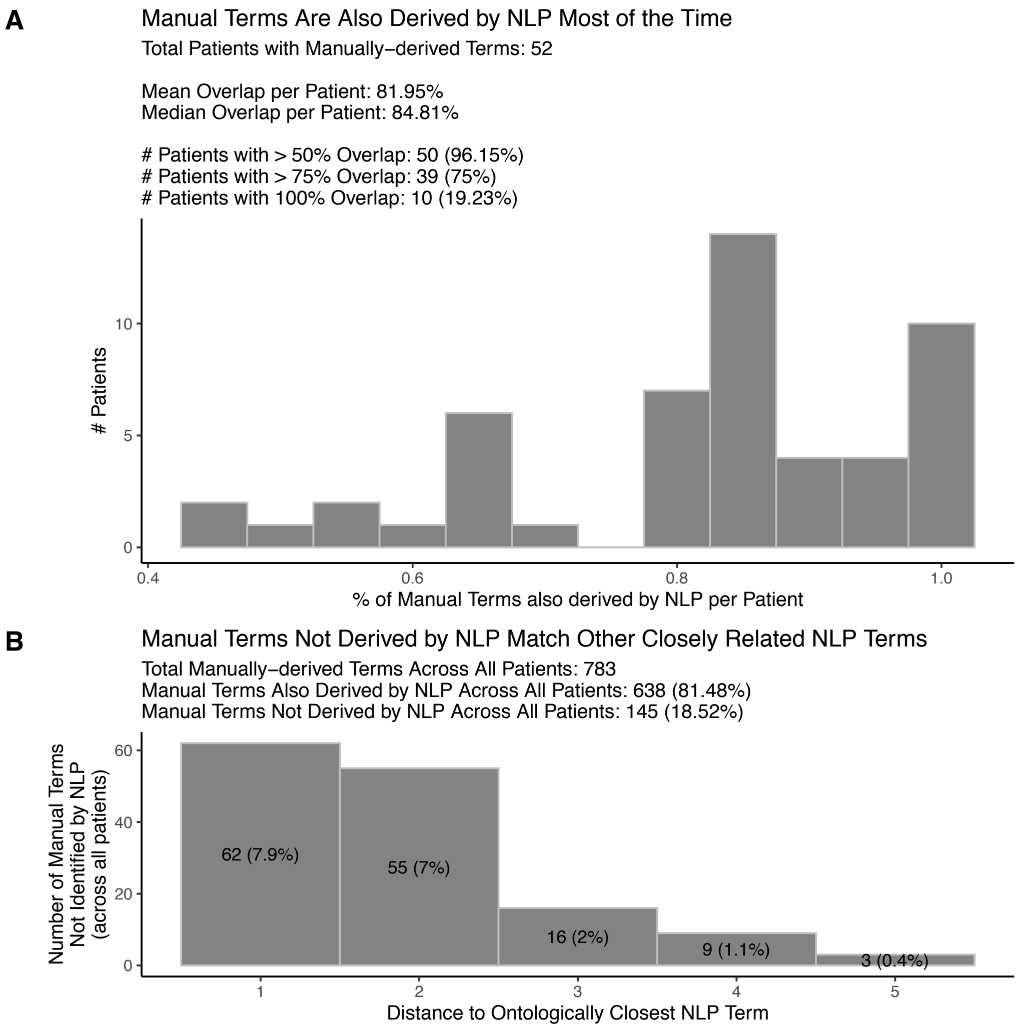

Figure S1. Overlap between manually-extracted terms and NLP-extracted terms per patient
(A) We computed the intersection between the set of terms extracted using manual curation with those terms extracted using NLP per patient as a percentage of the number of manually-extracted terms. On average, 81.95% of manually-extracted terms were also identified by NLP. The median percentage of overlapping terms was higher at 85%. 51/52 (96%) of the patients had at least half the manually-extracted terms identified via NLP.
(B) We searched for related NLP terms to those manual terms not identified by NLP. We calculated the undirected distance between the nearest NLP term in the HPO directed acyclic graph (DAG) to each of the 146 manual terms not also identified by NLP (18.5% of all terms). Only 28 manual terms (3.5% of all terms) did not have a close ontologically related (within 2 steps) term in the NLP-derived list.

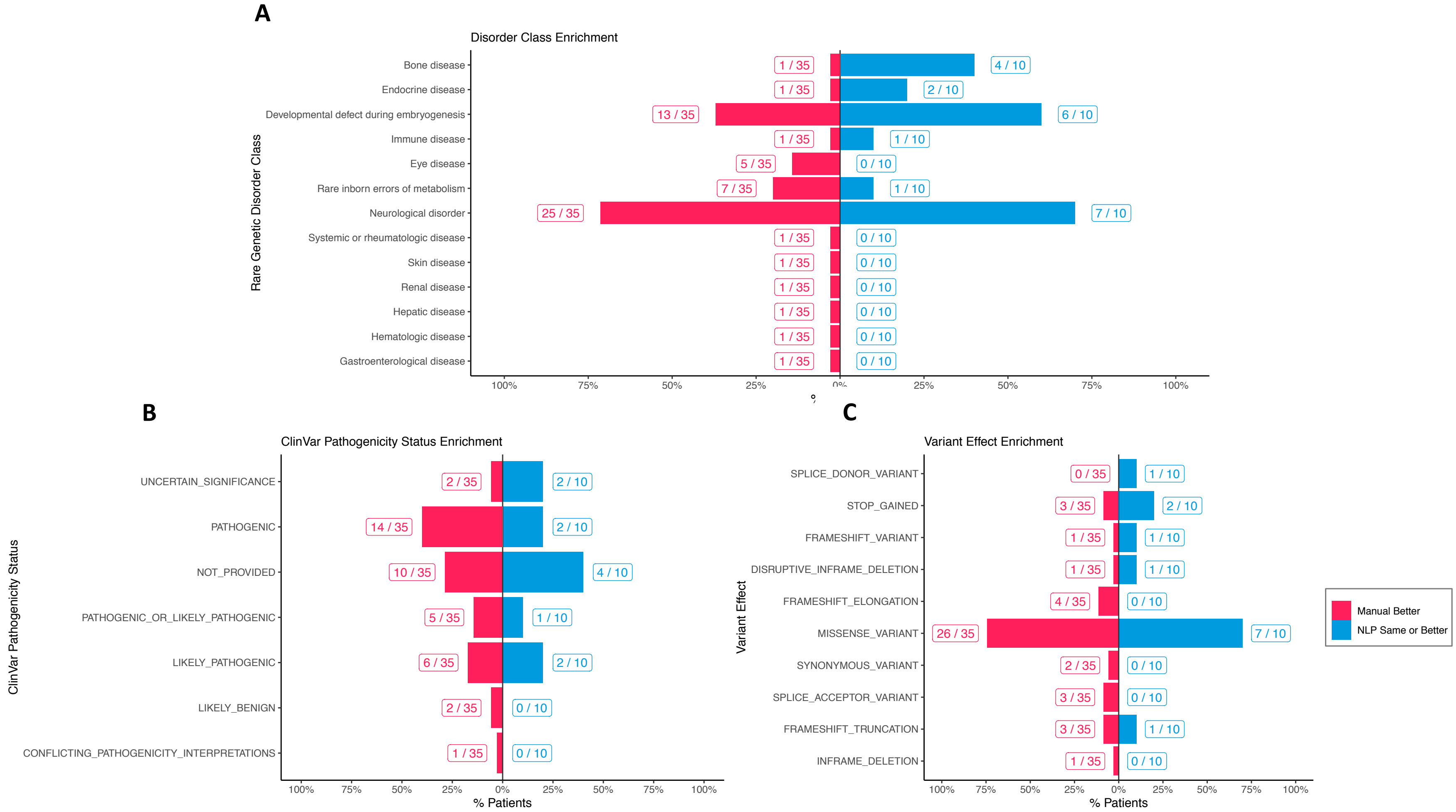

Figure S2. Comparing enrichment of disease and variant characteristics in patients grouped based on whether or not manually-extracted HPO terms led to better gene ranks in Exomiser

We compared (A) the Orphanet disorder class for the diagnosed disease, (B) the ClinVar pathogenicity status, and (C) the variant effect in the set of patients where manual phenotyping ranked better with the same attributes in the set of patients where NLP-based phenotyping ranked the same or better as the manual approach. These differences were not statistically significant. Statistical significance was determined if the p-value computed using Fisher's Exact Test with Benjamini-Hochberg multiple hypothesis correction was less than 0.05.

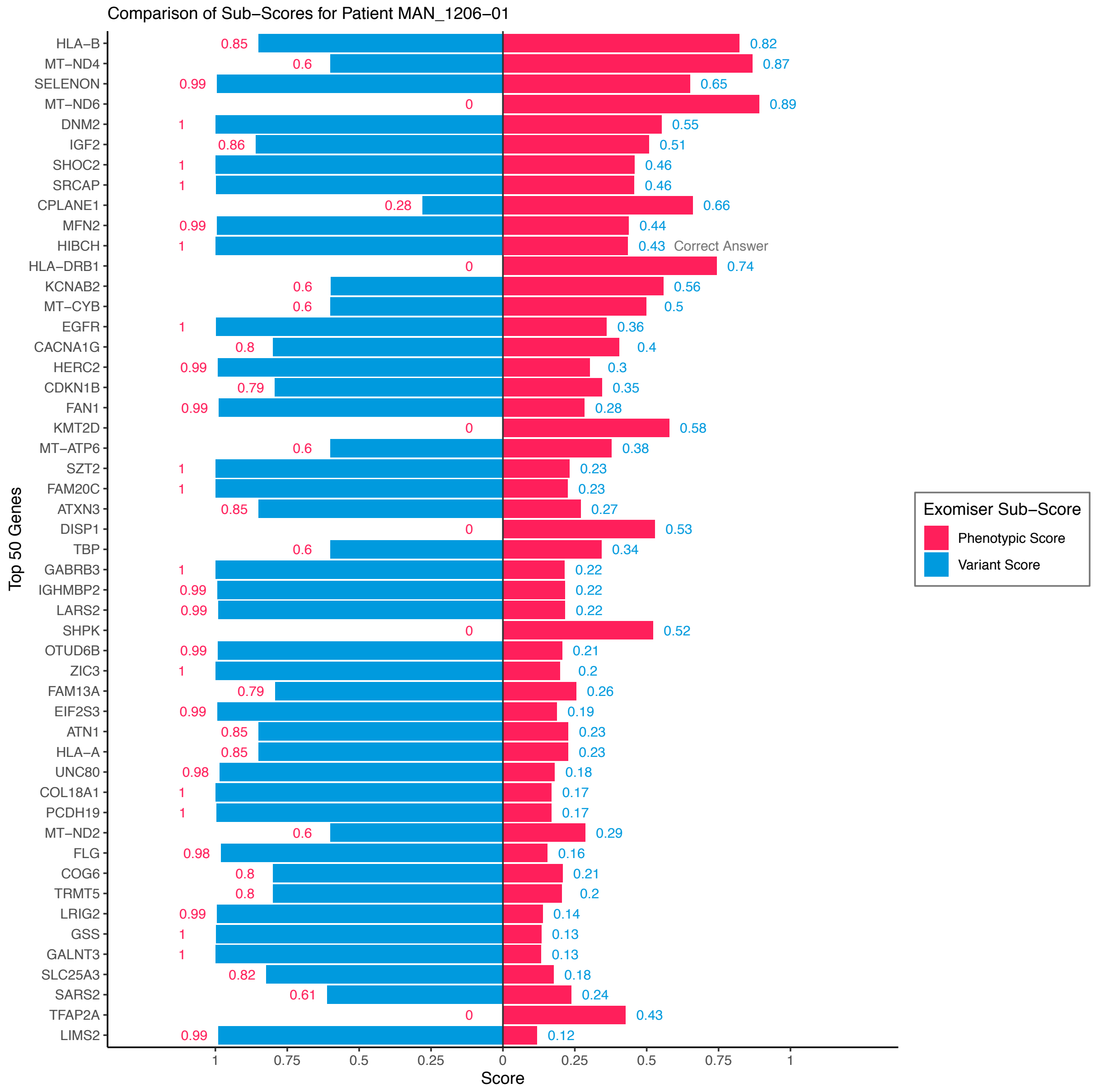

Figure S3. Exomiser phenotypic and variant sub-scores for the top 50 genes for a representative patient with known diagnosis

We consider top 50 to be a practical limit of genes that a clinician or researcher may manually evaluate. In the top 50 genes, the variant score was almost consistently high (> 0.8) and appears closer to a binary measure than a continuous one. On the other hand, the phenotypic score correlated with the gene rank. Therefore, we hypothesized that improved phenotyping may improve the desired gene rank. The correct causal gene for this patient was ranked 11. Simply prioritizing by the phenotypic score, however, would have dropped the ranking for the causal gene down to 17. In 4 of the 6 genes that had a higher phenotypic score, the variant score was 0 further supporting the idea that the variant score may serve as a binary admission criterion.

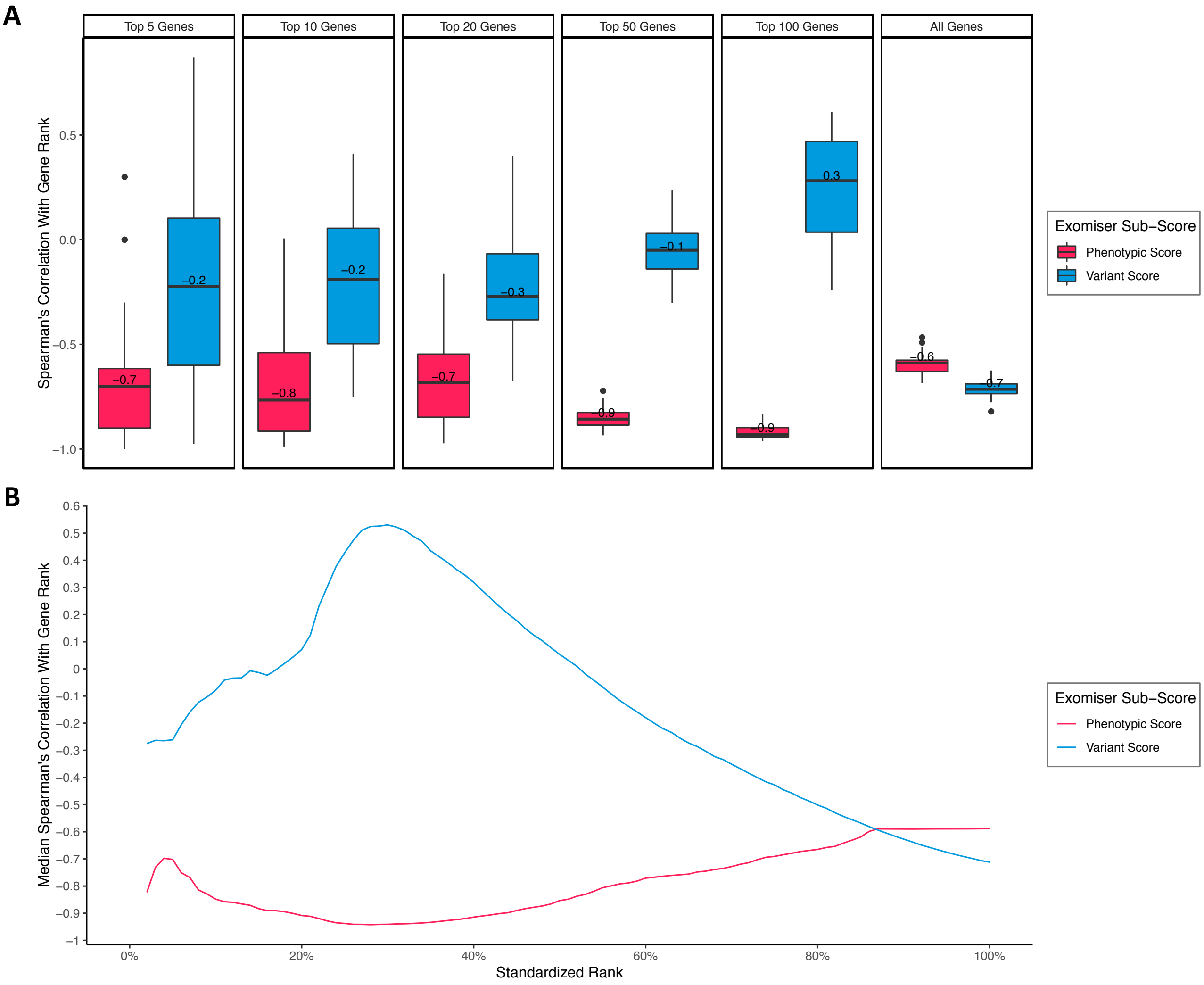

Figure S4. Distributions of correlations between gene ranks and sub-scores across patients in the training set

(A) To assess whether the observed correlation between phenotypic sub-score and gene rank was unique to the aforementioned example patient and in the top 50 genes, we computed the Spearman's correlation between each of the phenotypic and variant sub-scores with gene rank within the top 5, 10, 20, 50, 100, and entire gene lists for all 52 patients. Note that a correlation of -1 is the perfect desired correlation between a score (higher value is better) and gene rank (lower value is better). The distribution of variant sub-score correlations with gene rank in all set of top N genes (except for the entire gene list) crossed 0 (no correlation) and was consistently worse than the correlation between phenotypic sub-score and gene rank.

(B) The median correlation across 52 patients was computed between each of the phenotypic and variant sub-scores with gene rank for all possible gene rank cutoffs, standardized between 0 and 100%. The median correlation between variant score and gene rank is not better than the correlation between phenotypic score and gene rank until the tail end (bottom 15%) of the ranked genes.

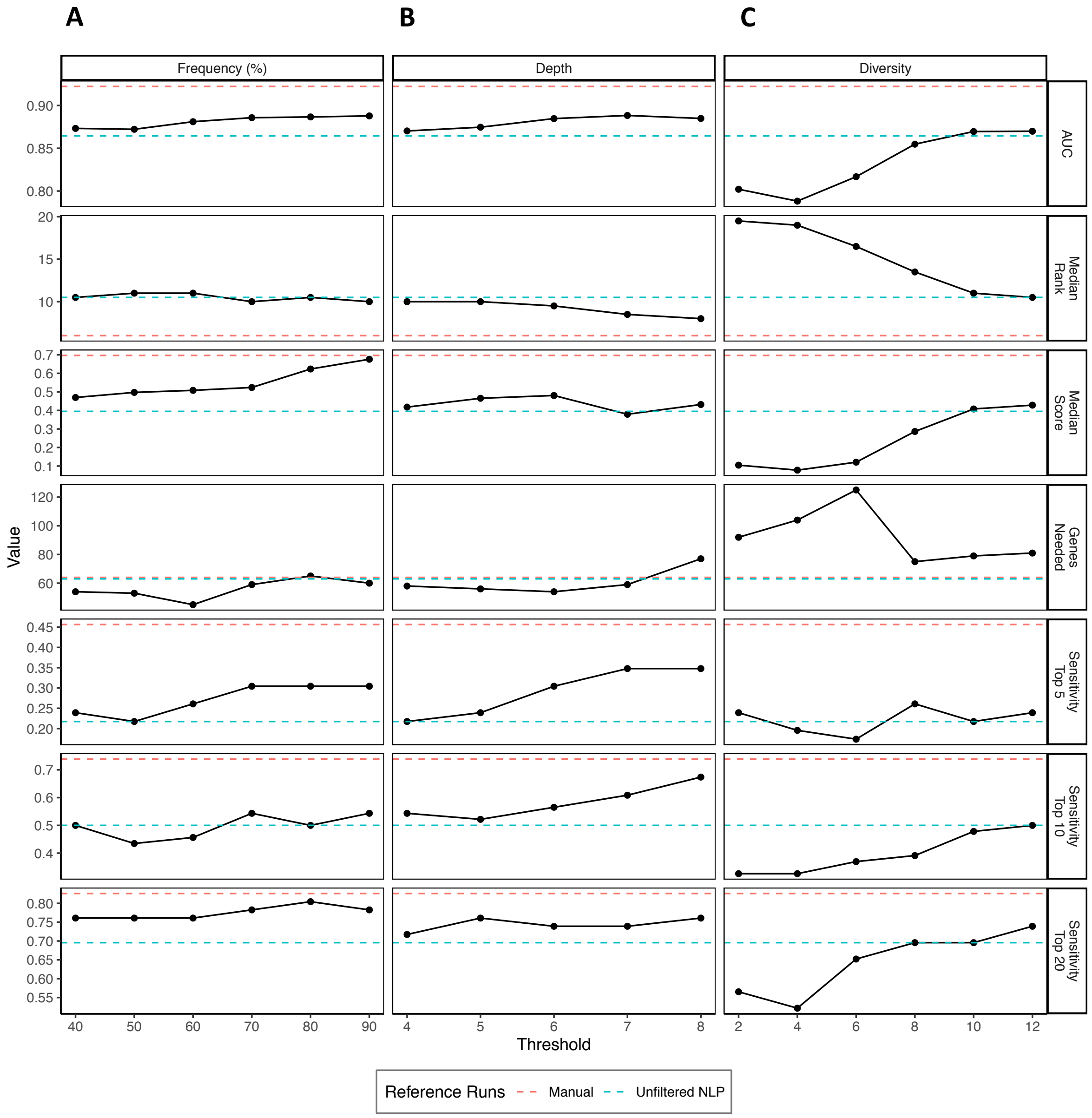

Figure S5. Exomiser performance on the training set after filtering NLP-extracted HPO terms per patient based on each term feature independently

(A) Performance after filtering for most frequently occurring HPO terms per patient as determined by frequency percentile thresholds between 40% and 90%.

(B) Performance after filtering for HPO terms per patient that are at least as deep as the specified depth threshold between 4 and 8.

(C) Performance after filtering for HPO terms per patient that belonging to the top n represented phenotypic abnormality classes where n is the diversity threshold ranging between 2 and 12, and phenotypic abnormality classes are ranked by mean frequency. Each row of plots corresponds to a different measure of Exomiser performance, *e.g.* the top row describes AUC as a function of varying the frequency, depth, and diversity thresholds respectively. The dashed red and blue lines in each plot indicate the Exomiser performance measure when using the manually-extracted and unfiltered NLP-extracted HPO terms respectively.

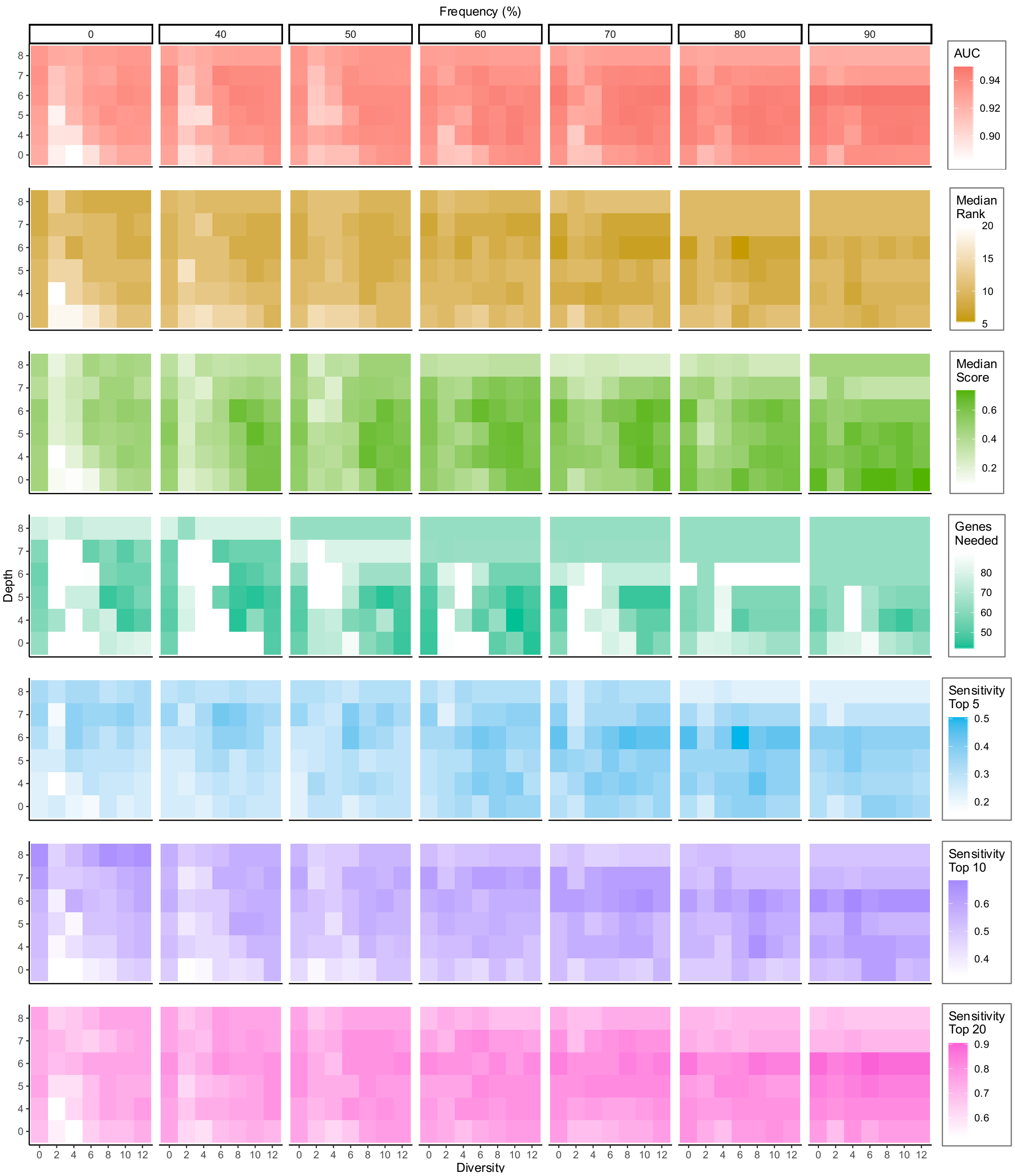

Figure S6. Exomiser performance on the training set after filtering NLP-extracted HPO terms per patient based on all possible combinations of term feature thresholds

There are 49 heatmap plots corresponding to 7 performance metrics (rows, colors) times 7 different frequency percentile thresholds (columns). Each heatmap plots the respective performance metric (color) for a given depth (y-axis) and diversity (x-axis) threshold. Therefore,

each row describes Exomiser performance as defined by a specific metric (*e.g.* AUC in the top row) for all possible 294 filter parameters determined by the combination of frequency percentile, depth, and diversity thresholds. Color scales are from minimum to maximum for each performance metric except for "Genes Needed", which was truncated to the interval 41 (actual minimum) to 90 (not the maximum) due to a few very high outliers.

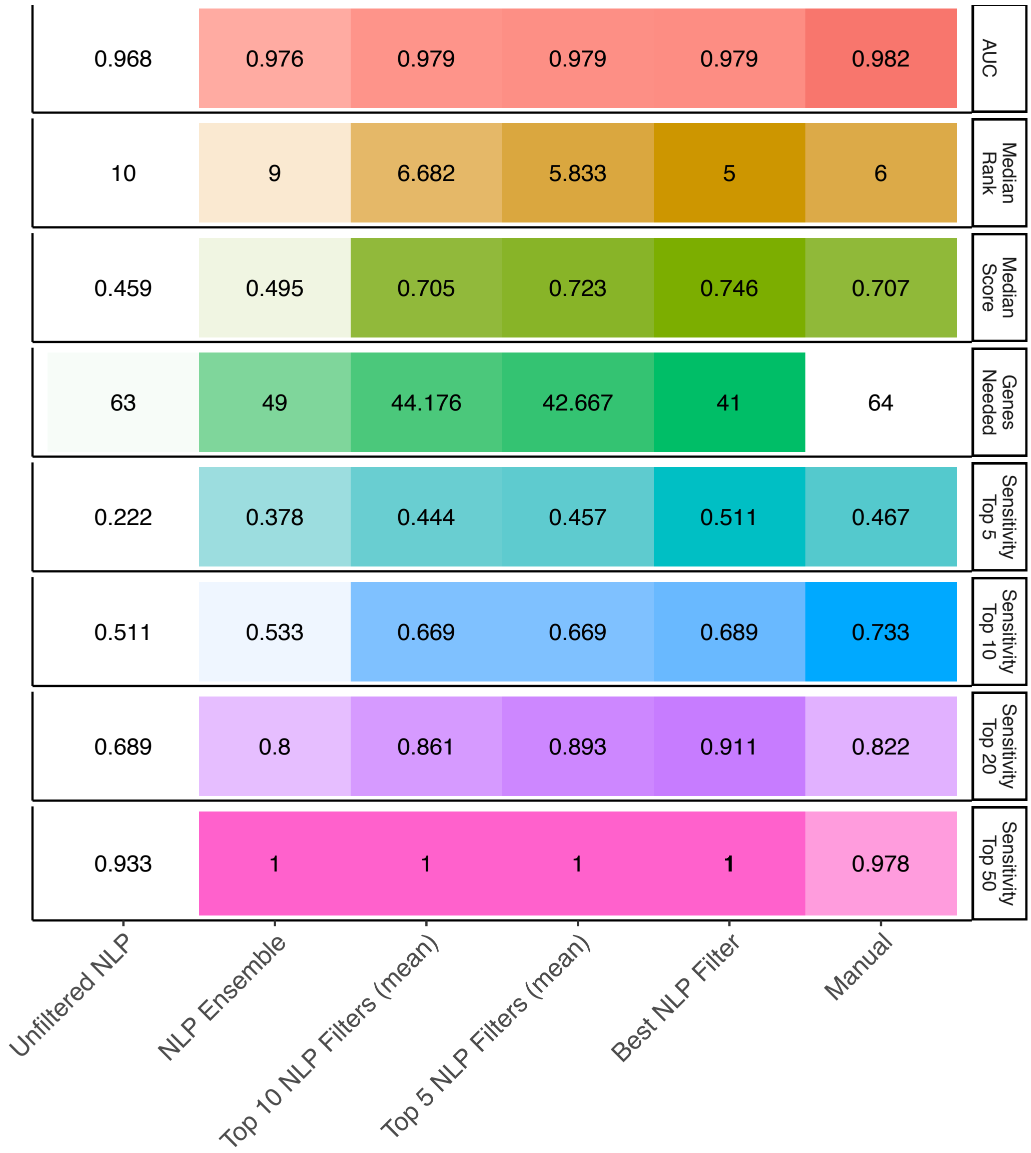

Figure S7. Exomiser performance on the training set for the best performing filter combinations compared to performance using manual and unfiltered NLP-based phenotype extraction

Each row (and color) corresponds to a different performance metric. The color intensity from light to dark represents better relative performance. The columns are bookended by Exomiser performance when using the unfiltered NLP-extracted and manual-extracted terms. The "NLP Ensemble" is based on the average score per gene across all 294 possible NLP filter parameter combinations. The "Best NLP Filter" represents a specific combination of filter parameters, which may vary per performance metric. The values in the "Top 5" and "Top 10" NLP Filters columns are the average of the top 5 and top 10 Exomiser performances per metric. Note that the number of parameter combinations corresponding to the top 5 and top 10 performances may be more that 5 and 10 respectively in the case of tied performance values. Similar to "Best NLP Filter", the sets of filter parameter combinations corresponding to the "Top 5" and "Top 10" performances may vary per metric.

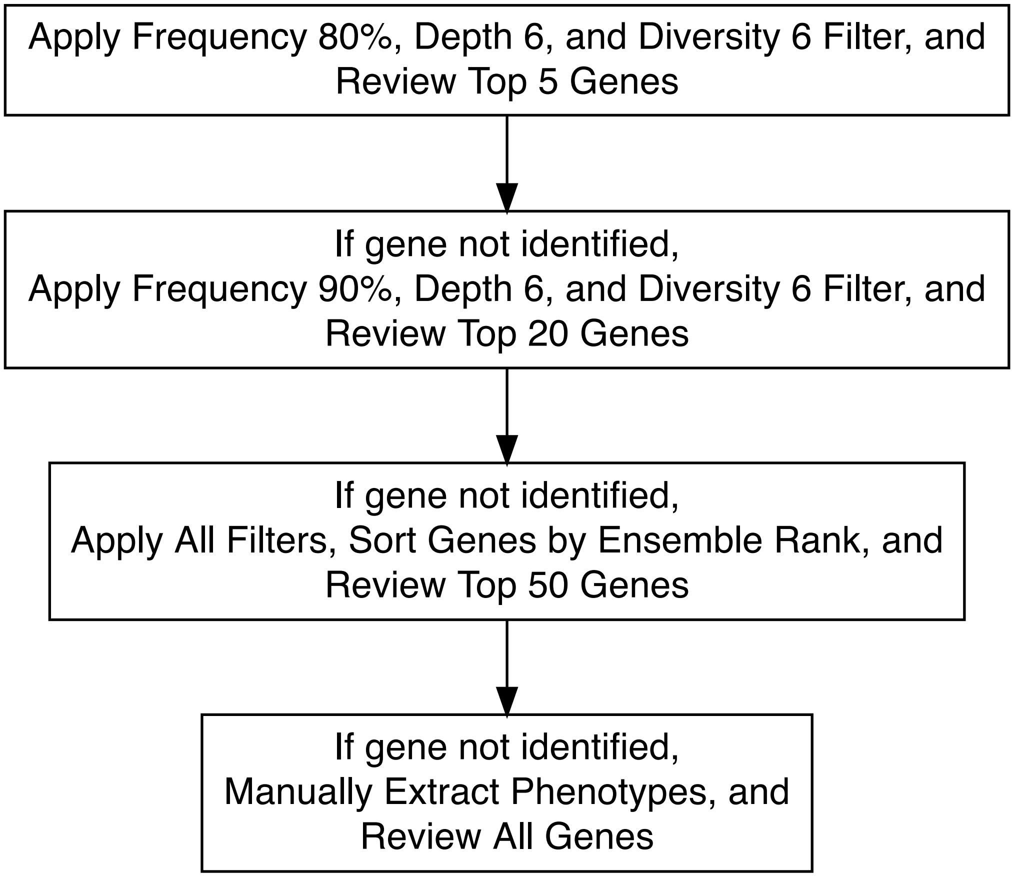

Figure S8. Flow chart describing the tiered approach to filtering NLP-extracted phenotypes and running gene prioritization
Based on our analysis of the effect of filtering NLP-extracted terms on improved gene prioritization, we constructed a 4-step tiered approach that incrementally increases the number of genes to consider.

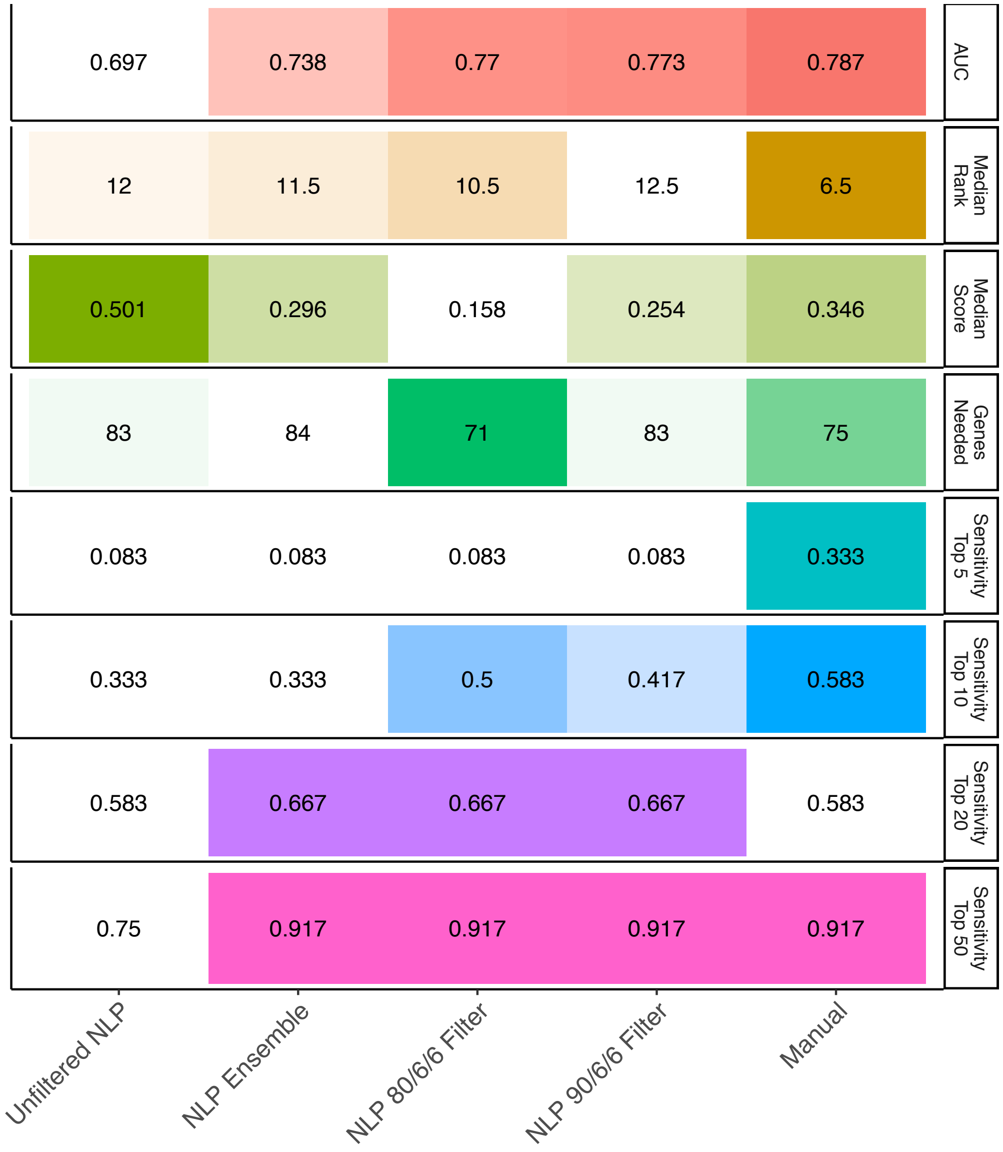

Figure S9. Exomiser performance on the test set for the filter combinations used in the tiered approach to phenotype extraction

Each row (and color) corresponds to a different performance metric. The color intensity from light to dark represents better relative performance. The algorithms (columns) are sorted by their overall performance (AUC) rather than the tier. “Unfiltered NLP” is included as a reference.

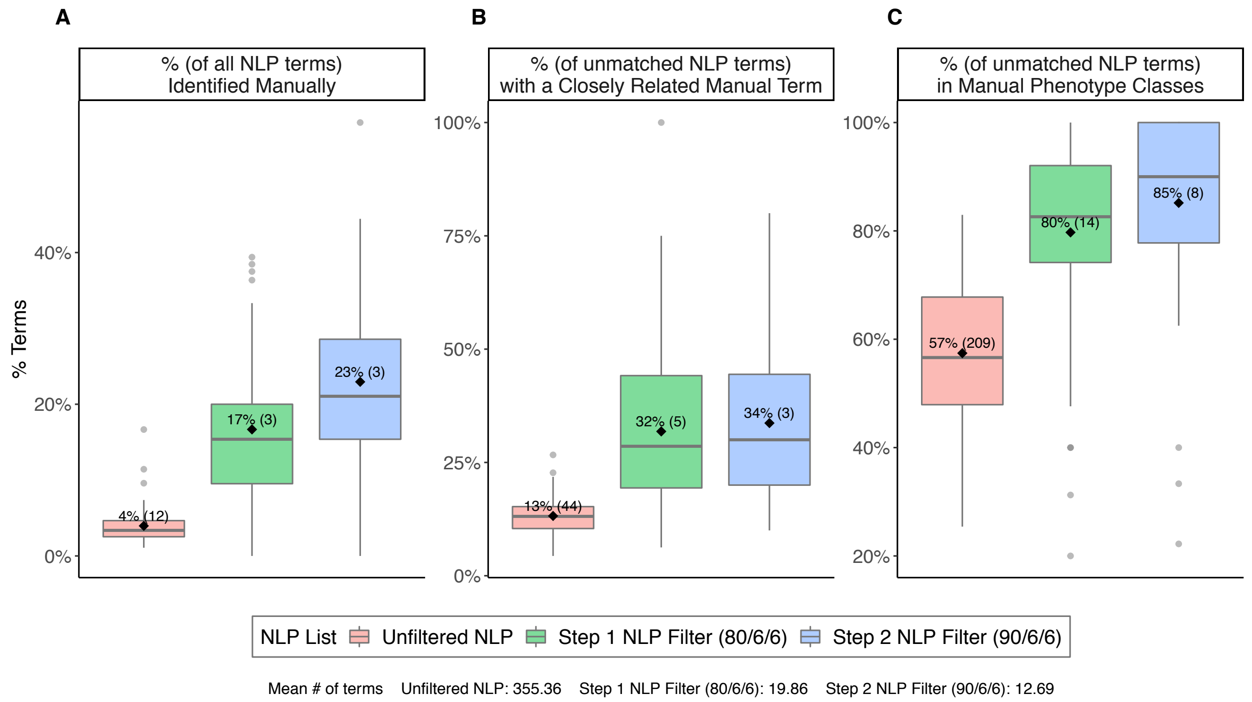

Figure S10. Box and whisker plots of distributions of percentage of manual and related terms before versus after NLP filtering
For each of the 64 patients in this study, we computed the (A) percentage of terms in the lists of unfiltered NLP terms, NLP terms remaining after the 80/6/6 filter, and NLP terms remaining after the 90/6/6 that were also identified manually. For the NLP terms that were not also identified manually, we computed for each patient the (B) percentage of those unmatched terms in the aforementioned lists that were closely related to a manually identified term (undirected ontological distance ≤ 2), and (C) the percentage of those unmatched terms that belonged to one of the phenotypic abnormality classes represented amongst the manually extracted terms. Mean percentages with the mean number of terms in parentheses are noted for each distribution. The mean number of terms per NLP list are noted below the figure legend for reference.

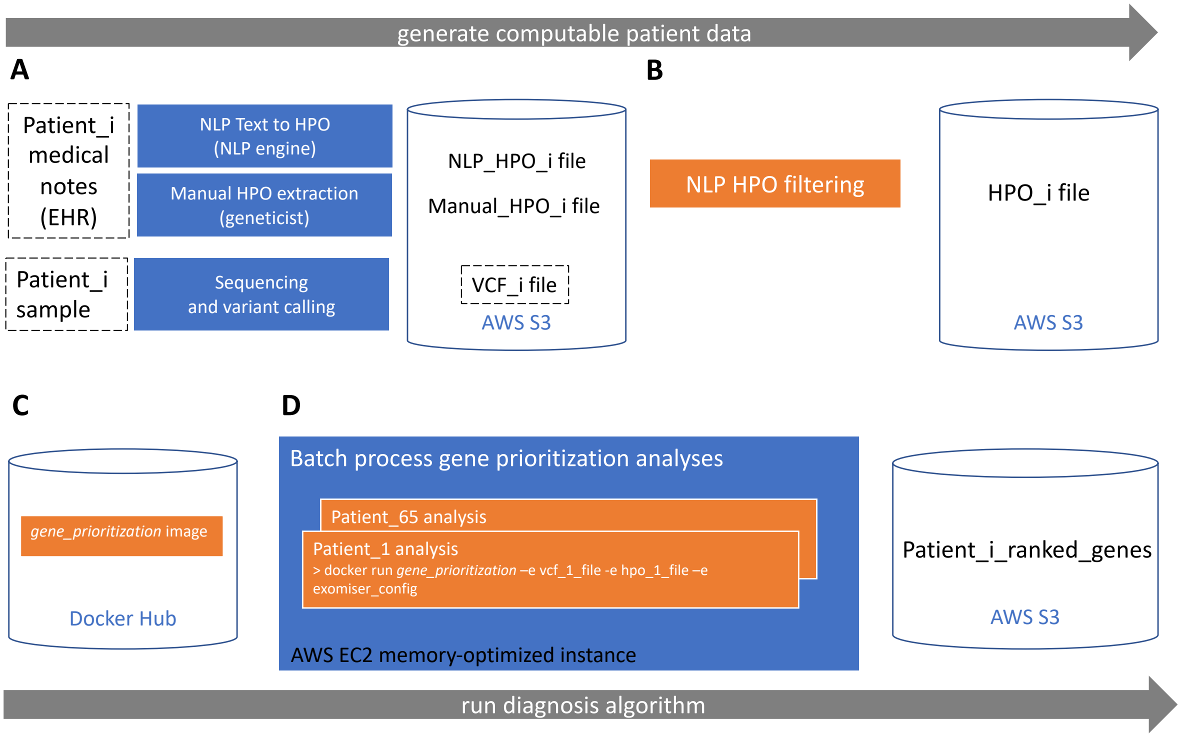

Figure S11. Modular architecture for implementing our phenotypic data-driven rare disease diagnosis pipeline

(A) For each patient i, the variant prioritization pipeline requires a VCF file describing the called variants and a list of HPO terms, extracted using NLP or by a manual expert curator. The files are stored within AWS S3. We used Clinithink's CLiX focus as our choice of NLP engine, which serves as a module with other options such as CLAMP.

(B) Custom post-processing scripts are then applied to the NLP-extracted HPO terms. We described 3 different filters to apply: frequency, depth, and diversity. Previous studies have shown the benefit of applying other filters such as removing common terms based on a reference set of EHRs[15], which could be applied at this step. The resulting post-processed HPO terms are stored in S3.

(C) The core variant prioritization tool is saved as docker[12] image in Docker Hub[13]. The containerized application takes as input a VCF file, a set of HPO terms, and configuration options and outputs a list of prioritized genes. We used Exomiser as our choice of gene prioritization tool, which could be replaced with other options such as MOON[17,18].

(D) We employ a single high memory AWS EC2 memory-optimized instance (r5n.16xlarge) to run batches of 65 patient-HPO set combinations at a time, with each run requiring 4GB memory (minimum 260GB memory required). The results are also stored in AWS S3, allowing the entire process to be run on the cloud.

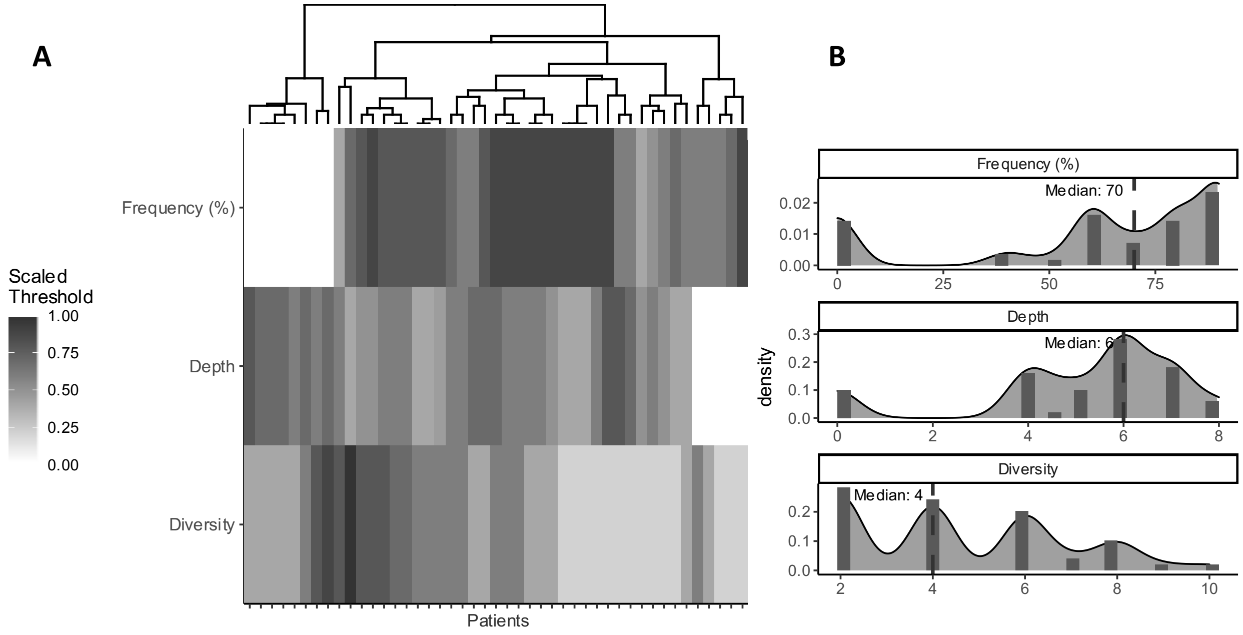

Figure S12. Patterns in parameter thresholds for optimal NLP filter combinations per patient in the training set

(A) Heatmap of the optimal filter combinations (x-axis) per patient (y-axis). Patients are hierarchically clustered based on the scaled values of the optimal filter combination thresholds. Optimal filter combinations are determined by gene rank first and then by the Exomiser score to break ties. If multiple combinations were still tied, we assigned the median value of the threshold per parameter to that patient as the optimal combination. The frequency threshold is scaled by a factor of 100. The depth and diversity thresholds are scaled by a factor of 10. All of the best diversity filter thresholds per patient were between 2-10 clades; none used the top 12 clades. Therefore, the resulting values in the heatmap have values between 0 and 1. There were 2 clear clusters of patients: a cluster of 7 patients with an absent frequency filter and a cluster of 5 patients with an absent depth threshold. Other than the aforementioned 2 clusters, there were no discerning patterns in the optimal thresholds or distinct clusters of patients.

(B) Distributions (probability densities) with median threshold value listed for each of the three parameters comprising the optimal filter combinations per patient. The median values for the frequency percentile, depth, and diversity thresholds in the optimal filter combinations were 70, 6, and 6 respectively.

SUPPLEMENTAL TABLES

Table S1. Genotypes of the probands in the training and test sets.

Supplementary_subject_genotypes.xlsx
Genotypes of the 53 probands in the training set and 12 probands in the test set. Available online at the Journal website.

Table S2. Patient demographics.

	N	% female (n)	Mean age (years)	Median year sequenced (range)	Mean no. of manual HPO terms (range)	Mean no. of NLP HPO terms (range)
Training Set	52	57% (30)	8.13 (0.05-24)	2014 (2012-2018)	15.1 (4-32)	379.8 (70-735)
Test Set	12	25% (3)	6.2 (0.04-16.25)	2017 (2014-2020)	12.6 (7-25)	249.5 (18-561)

Table S3. Depth and phenotypic abnormality classes per HPO term.

Table_S3_HPO_depth_abnormality_classes.xlsx
The depth and phenotypic abnormality classes per HPO term calculated using the shortest directed path (unweighted breadth-first search) from the root node of the HPO directed acyclic graph. Available online at the Journal website.

Table S4. Breakdown of patients in the training set by relative Exomiser rankings of the correct gene using manual- versus NLP-based phenotype extraction.

Exomiser Correct Gene Rank Comparison	Number of Patients (%)
Manual Ranked Better	35 (77.8%)
NLP Ranked Better	5 (11.1%)
Manual and NLP Ranked the Same	5 (11.1%)
Total Patients	45

Table S5. Performance of all 294 combination filters.

Table_S5_all_combinations_performance_landscape.xlsx
All 7 performance metrics (AUC, Median Rank, Median Score, Genes Needed, Sensitivity in the top 5 genes, Sensitivity in the top 10 genes, and Sensitivity in the top 20 genes for each of the 294 NLP combination filters and the manually phenotyping for reference. NLP combination filters (column "run_name") are described by the frequency percentile threshold, depth, and diversity with prefixes "fp", "d", and "c" respectively. The thresholds are also explicitly listed. Available online at the Journal website.

Table S6. Parameter combinations for the top performing NLP filters applied to the training set.*

Filtering Criteria for Top Combinations	Best NLP Filter			Top 5 NLP Filters Median (MAD)			Top 10 NLP Filters Median (MAD)		
	Frequency (%)	Depth	Diversity	Frequency (%)	Depth	Diversity	Frequency (%)	Depth	Diversity
AUC	90	6	6	90 (0)	6 (0)	8 (2.97)	90 (0)	6 (0)	8 (2.97)
Median Rank	80	6	6	70 (0)	6 (0)	7 (5.93)	70 (7.41)	6 (0.74)	8 (4.45)
Median Score	90	0	12	90 (0)	0 (0)	8 (2.97)	70 (22.24)	2 (2.97)	10 (2.97)
Genes Needed	60	4	10	55 (7.41)	4.5 (0.74)	10 (0)	60 (14.83)	5 (0)	10 (2.97)
Sensitivity Top 5	80	6	6	80 (0)	6 (0)	8 (2.97)	70 (14.83)	6 (0)	7 (1.48)
Sensitivity Top 10	90	6	4	80 (14.83)	6 (0)	8 (2.97)	80 (14.83)	6 (0)	8 (2.97)
Sensitivity Top 20	90	6	6	90 (0)	6 (0)	8 (2.97)	90 (0)	6 (0)	8 (5.93)
Overall Median (MAD)	90 (0)	6 (0)	6 (0)	80 (14.83)	6 (0)	8 (2.97)	70 (14.83)	6 (0)	8 (2.97)

*Note that Top 5/10 may have more than 5 or 10 filter combinations if they are tied. Therefore, the grand median (and analogous for MAD) for the top 5/10 may be different than the median of medians.

Table S7. Rank of the correct gene per patient in the prospectively evaluated test set using different phenotype extraction and filters.

Patient	NLP	NLP Ensemble	NLP 80/6/6 Filter	NLP 90/6/6 Filter	Manual	Best NLP Filter Rank	Change in Rank Post Filtering
MAN_1787	11	11	9	11	7	9	-2
MAN_1845	59	38	37	30	25	30	-29
MAN_1795	22	24	22	22	21	22	0
MAN_1802	2	1	2	2	2	1	-1
MAN_0943	12	6	7	14	6	6	-6
MAN_1011	83	84	71	83	75	71	-12
MAN_0469	7	7	7	6	6	6	-1
MAN_0805	57	49	42	26	34	26	-31
MAN_0842	29	20	13	6	33	6	-23
MAN_0678	12	12	9	18	2	9	-3
MAN_0520	10	11	12	10	5	10	0
MAN_1886	7	7	7	7	5	7	0

Table S8. The number of patients where each NLP combination filter was applicable.

Table_S8_num_patients_filtered_histogram.xlsx
The number of patients that each NLP combination filter was applied to. If a combination filter led to fewer than 5 HPO terms remaining for a given patient, then the combination filter was not applied to that patient. Especially stringent combination filters (e.g. limited to 90% frequency percentile, at least 8 levels deep, and top 2 abnormality classes) result in very few patients with filtered terms (column “num_patients_filtered”). The combination filter (column “run”) is described by the frequency percentile threshold, depth, and diversity with prefixes “fp”, “d”, and “c” respectively. Available online at the Journal website.

Table S9. Optimal phenotype extraction option based on gene rank for patients in the training set.

Optimal Phenotype Extraction Option	Number of Patients (%)
Best NLP Filter	33 (73.3%)
Best NLP Filter, or NLP Ensemble	3 (6.7%)
Best NLP Filter, or Manual	3 (6.7%)
Any	3 (6.7%)
Manual	2 (4.4%)
Best NLP Filter, or Unfiltered NLP	1 (2.2%)
Total	45

Table S10. Spearman's correlations between parameter thresholds for optimal NLP filter combinations per patient in the training set.*

Spearman Correlation (P-value)	Frequency (%) Threshold	Depth Threshold
Depth Threshold	-0.29 (0.052)	
Diversity Threshold	-0.08 (0.614)	0.11 (0.475)

*The two clusters of patients with absent frequency percentile and depth thresholds results in the strongest correlation between any two parameters. However, no pairs of parameters had statistically significant correlations (p-value < 0.05). The p-value for Spearman's correlation is computed using algorithm AS 89[24].

Table S11. Cross-tabulation of training set patients based on HPO-term extraction methods resulting in higher gene ranks before and after filtering.*

	Number of Patients (%) Optimal NLP Filter					
Optimal Pre-Filtering Term Extraction Method	[80 or 90]/6/6	80/6/6	90/6/6	Ensemble	Manual	Total
Manual	1 (2.86%)	10 (28.57%)	8 (22.86%)	0 (0%)	16 (45.71%)	35 (77.78%)
Unfiltered NLP	3 (30%)	1 (10%)	5 (50%)	1 (10%)	0 (0%)	10 (22.22%)
Total	4 (8.89%)	11 (24.44%)	13 (28.89%)	1 (2.22%)	16 (35.56%)	45

*Table rows divide patients based on whether manual- or NLP-extracted terms without filtering resulted in higher gene ranks (Table S4). Table columns divide patients based on which NLP filter from the tiered pipeline resulted in the highest gene ranks. Manual extraction is included in the post-filtering categories for reference. Ties in rank were resolved based on feasibility (Combination Filters [80/6/6 or 90/6/6] > Ensemble > Unfiltered NLP > Manual).
