# Supplementary material for: A data-driven architecture using natural language processing to improve phenotyping efficiency and accelerate genetic diagnoses of rare disorders
Source: HGG Adv. 2021 May 11;2(3):100035. doi: 10.1016/j.xhgg.2021.100035 (PMC8432593; doi:10.1016/j.xhgg.2021.100035)
Supplement: Document S2. Article plus supplemental information [file mmc6.pdf]

HGG
Advances

ARTICLE

A data-driven architecture using natural language processing to improve phenotyping efficiency and accelerate genetic diagnoses of rare disorders

Jignesh R. Parikh,[1,7] Casie A. Genetti,[2,7] Asli Aykanat,[2] Catherine A. Brownstein,[2] Klaus Schmitz-Abe,[2] Morgan Danowski,[2] Andrew Quitadomo,[2,4] Jill A. Madden,[2] Calum Yacoubian,[5] Richard Gain,[5] Tessa Williams,[5] Mary Meskell,[5] Andrew Brown,[5] Alison Frith,[5] Shira Rockowitz,[2,4] Piotr Sliz,[2,4] Pankaj B. Agrawal,[2,6] Thomas Defay,[3] Paul McDonagh,[3,8] John Reynders,[3,9] Sebastien Lefebvre,[3,*] and Alan H. Beggs[2,*]

Summary

Effective genetic diagnosis requires the correlation of genetic variant data with detailed phenotypic information. However, manual encoding of clinical data into machine-readable forms is laborious and subject to observer bias. Natural language processing (NLP) of electronic health records has great potential to enhance reproducibility at scale but suffers from idiosyncrasies in physician notes and other medical records. We developed methods to optimize NLP outputs for automated diagnosis. We filtered NLP-extracted Human Phenotype Ontology (HPO) terms to more closely resemble manually extracted terms and identified filter parameters across a three-dimensional space for optimal gene prioritization. We then developed a tiered pipeline that reduces manual effort by prioritizing smaller subsets of genes to consider for genetic diagnosis. Our filtering pipeline enabled NLP-based extraction of HPO terms to serve as a sufficient replacement for manual extraction in 92% of prospectively evaluated cases. In 75% of cases, the correct causal gene was ranked higher with our applied filters than without any filters. We describe a framework that can maximize the utility of NLP-based phenotype extraction for gene prioritization and diagnosis. The framework is implemented within a cloud-based modular architecture that can be deployed across health and research institutions.

Introduction

Over the past decade, the introduction of next-generation sequencing has revolutionized the diagnosis and discovery of rare monogenic conditions. Exome sequencing (ES) has been shown to be an effective first-tier test for the diagnosis of a variety of congenital and neurodevelopmental phenotypes.[1,2] The technical ability to generate high-quality genomic data in a timely manner has reached a plateau, and significant progress has been made in the field of variant interpretation, particularly in the coding region of the genome.[3] Despite these advances, the diagnostic rate of ES remains relatively low at 25%–50%.[2,4,5] Pathogenic variants in a significant percentage of these undiagnosed cases may be hidden in poorly understood non-coding regions or in the approximately 15,000 genes that have yet to be associated with human disease.[6] Nevertheless, it is clear that a lack of accurate and deep phenotyping to correlate with genotypic findings remains a major issue in variant interpretation, especially in high-throughput clinical diagnostic situations.[7–10] The process of deep phenotyping, whether through clinical encounter or medical record review, is a labor- and time-intensive process requiring a high degree of expertise.[11,12]

Natural language processing (NLP) has been adopted as a scalable approach to automate the extraction of phenotypic information from electronic health records (EHRs). Standardization of outputs by encoding clinical information using the Human Phenotype Ontology (HPO) in a high-throughput manner has great potential to help shorten the diagnostic odyssey, thereby reducing costs and improving care.[13,14] However, given idiosyncrasies and variation in the structure and content of different EHR systems and notes from health care providers, it has been a challenge to develop automated phenotyping approaches comparable or superior to manual curation to facilitate the diagnosis of genetic diseases.[11,12]

The goal of this project is to provide a replicable framework to maximize the utility of NLP-based phenotype extraction from EHRs for use with gene prioritization algorithms. Here, we compare the efficacy of a gene prioritization tool, Exomiser,[15] in correctly identifying the disease-causing gene in previously diagnosed children using manual phenotyping by an expert curator versus

[1]J Square Labs, LLC, Natick, MA 01760, USA; [2]The Manton Center for Orphan Disease Research, Division of Genetics and Genomics, Boston Children's Hospital, Harvard Medical School, Boston, MA 02115, USA; [3]Alexion Pharmaceuticals, Inc., Boston, MA 02210, USA; [4]Computational Health Informatics Program, Boston Children's Hospital, Harvard Medical School, Boston, MA 02115, USA; [5]Clinithink, Ltd., London N1 6DR, UK; [6]Division of Newborn Medicine, Boston Children's Hospital, Harvard Medical School, Boston, MA 02115, USA
[7]These authors contributed equally to this work
[8]Present address: Sema4, Stamford, CT 06902, USA.
[9]Present address: Latent Strategies, LLC, Newton, MA 02465, USA.
*Correspondence: beggs@enders.tch.harvard.edu (A.H.B.), sebastien.lefebvre@alexion.com (S.L.)
https://doi.org/10.1016/j.xhgg.2021.100035.
© 2021 The Author(s). This is an open access article under the CC BY license (http://creativecommons.org/licenses/by/4.0/).

automated NLP extraction from the EHR. Utilizing differences identified between the manual and NLP-extracted HPO terms, we constructed a tiered pipeline that automatically filtered NLP-extracted HPO terms and improved gene prioritization in prospectively evaluated cases.

Our approach enabled NLP-based extraction of HPO terms to be a sufficient replacement for manual extraction, providing evidence for the utility of the tiered filtering approach in a high-throughput environment. Overall, we illustrate a framework for learning from already genetically diagnosed cases to maximize the utility of NLP via filtering methods and describe a modular software architecture to implement our framework for research and clinical applications, which can be replicated across different health care systems. Implementation of this scalable automated approach has potential to significantly reduce the manual effort required to phenotype patients with complex diseases and increase the efficiency of molecular genetic diagnostic programs.

Subjects and methods

Study overview

The goal of this study is to provide a replicable framework to maximize the utility of NLP-based phenotype extraction from EHRs for use with gene prioritization algorithms and is motivated by the need to reduce the manual effort required to evaluate prioritized variants/genes. Our hypothesis is that a solution lies in filtering NLP-extracted terms to more closely resemble manually extracted terms. Figure 1 summarizes our study design and analysis plan. Subsequent sections describe the patient cohorts and data used, and the methods and results for comparing manual- versus NLP-extracted HPO lists, translating observed differences to a tiered NLP filtering approach and evaluating gene prioritization performance on a subsequently ascertained test set.

Study subjects

All patients were ascertained through an existing rare disease gene discovery protocol of the Manton Center for Orphan Disease Research Gene Discovery Core at Boston Children's Hospital (BCH), and all provided informed consent under the supervision of the hospital's Institutional Review Board. Some were sequenced through the Children's Rare Disease Cohort initiative,[16] and data on a subset of these patients have been analyzed previously.[8,12] The study subjects represent probands with a variety of clinical presentations, all with a genetically diagnosed rare monogenic etiology (Table S1). Patients' ages for which most recent phenotypic data were available ranged from 0.04 to 24 years (mean = 7.69 years) (Table S2). All patients had at least one physician-authored outpatient record or inpatient consultation in the Boston Children's Hospital EHR system and genomic data in the form of a variant call file (VCF) available from ES. Manual curations of HPO terms for all cases were carried out by two expert curators by reading patient medical records and identifying or applying HPO terms using the HPO lookup tool incorporated in PhenoTips.[17] NLP extraction of HPO terms was performed by Clinithink's patented Clinical Natural Language Processing (CNLP) engine, CLiX[16] (see Supplemental subjects and methods). Clinical phenotypic data in the EHR were de-identified following extraction of HPO terms and related to de-identified genotypic data matched by study ID. All human studies described herein adhere to the principles set out in the Declaration of Helsinki, and every subject involved in this study provided informed consent in accordance with the ethical standards of the Boston Children's Hospital Institutional Review Board.

Phenotype data extraction

Patient records were stored in the Boston Children's Hospital Cerner Electronic Health Record (CERNER EHR) database, which enables integrated storage of different types of medical records from health care providers, including outpatient and inpatient records, consultations, surgical notes, and imaging and procedure forms, as well as lab results.

The patients' clinician-authored outpatient records or inpatient consultations in the EHR were used for both manual and Clinithink NLP curations. Scanned records, such as images from external health care institutions, were omitted.

Manual curations of HPO terms for all cases were carried out by two curators by reading patient medical records and identifying or applying HPO terms using the HPO lookup tool incorporated in PhenoTips.[2] The curators were trained genetic research assistants with 2 to 3 years of experience, under the supervision of a certified and licensed Master's degree level genetic counselor (C.A.G.), and with the oversight of a physician (P.B.A.) and a PhD molecular geneticist (A.H.B.). The curators were blinded to the genetic diagnosis of the patient. For each phenotype, the most precise term was picked depending on the definition of the HPO term. The curator was selective for terms potentially relevant to the patient's overall clinical presentation and useful for diagnosis and omitted less-relevant terms such as a single fever or trauma.

NLP extraction of HPO terms from 462 different document types from CERNER EHR was performed by Clinithink's patented CNLP engine, CLiX, using HPO Queryset v.11.2 (see Table S6 in Rockowitz et al.[16]).

Raw NLP-extracted terms included a number of different false positives in contrast to manual curations of medical records. The most significant source of false positives was physician's notes regarding differential diagnoses containing unconfirmed disorders. As an example, a patient with transient infantile hypertriglyceridemia had false positives like hyperglycosemia and abnormal amino-acid metabolism generated from a differential diagnosis list in an outpatient medical record. Other false positives generated by NLP included medication-induced symptoms and signs, terms generated from patient/physician names, and the misinterpretation of common words as clinical symptoms. Based on manual review of 6 sets of medical records (data not shown), we estimated 21% of raw NLP-extracted terms to be false positives.

Data, available at initiation of this project, for a training set of 52 patients with a known causal diagnostic variant(s) were utilized to establish the filtering methodology described herein. A test set, comprised of 12 similar cases ascertained subsequently, was used to prospectively test the filtering infrastructure to ensure reproducibility and effectiveness across multiple groups (Table S2).

NLP term features

We computed the following values per patient for a given set of NLP-extracted HPO terms: (1) mean frequency percentile, (2) mean depth, and (3) diversity.

Frequency percentile was calculated using the ranks of all HPO terms for a given patient based on term frequency; tied ranks

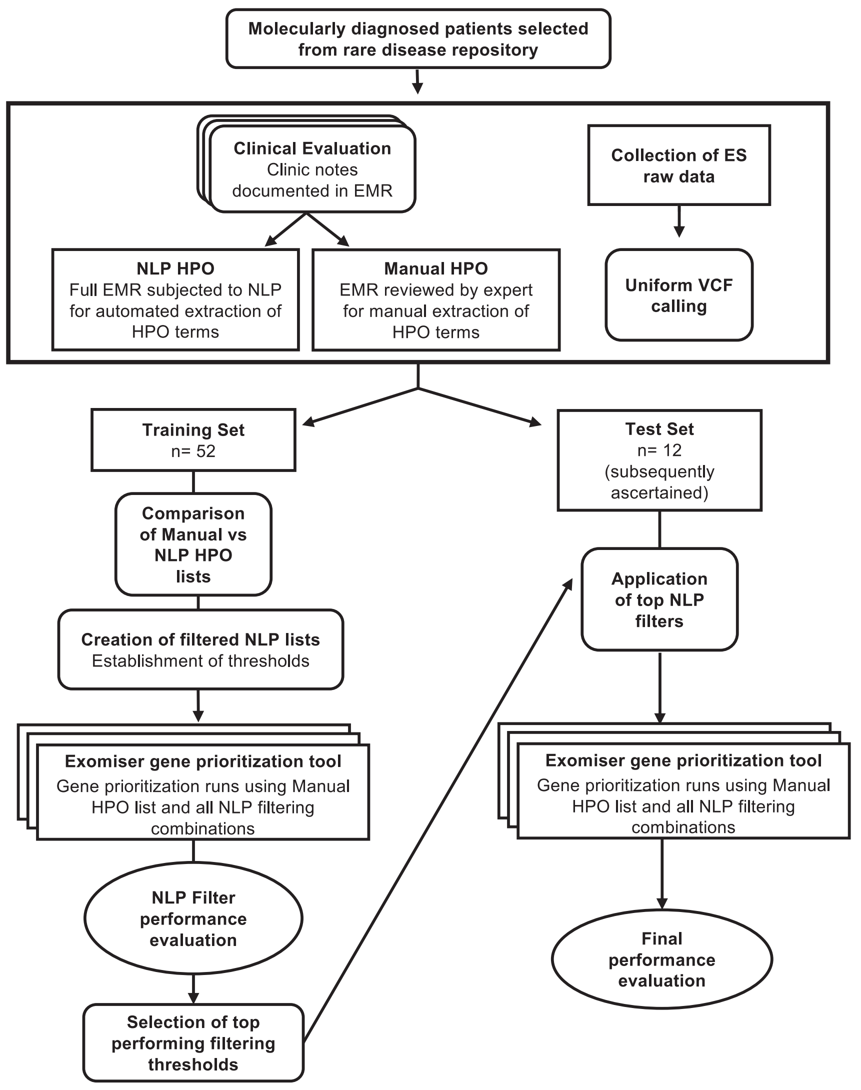

Figure 1. Study flow diagram
Schematic of the overall study design and analysis plan. For each patient in a training set of 52 patients, we employed uniform processes to collect Human Phenotype Ontology (HPO) terms extracted by natural language processing (NLP), manually extracted HPO terms, and exome sequencing (ES) data in the form of variant call files (VCFs). Manually extracted HPO terms were compared to NLP-extracted HPO terms per patient in the training set with respect to (1) frequency of use, (2) HPO term depth within the ontology, and (3) diversity of phenotypic abnormality classes captured, confirming significant differences across all three dimensions. Next, we established thresholds per dimension that were used to create filtered lists of NLP terms per patient. Exomiser was run on each of the filtered NLP term lists (in addition to the manual and unfiltered NLP lists for comparison) per patient, and performance per filter was evaluated using metrics such as area under the receiver operating curve (AUC) and sensitivity. Top-performing NLP filters were combined into a tiered pipeline, which was finally applied to and evaluated on a subsequently ascertained set of 12 patients in the test set, whose data were collected using the same uniform processes described above.

were averaged. Depth was calculated as the distance of the shortest directed path from the root node in the HPO ontology to the respective term using an unweighted breadth-first search. Each term was assigned all unique phenotypic abnormality classes that its shortest paths passed (Table S3). We defined diversity as the number of unique phenotypic abnormality classes represented within a given set of HPO terms. We utilize the diversity and depth features as a proxy for term specificity in our analysis below (see Supplemental subjects and methods for additional details).

Comparing distributions of NLP-extracted versus manually extracted terms

We split the NLP-extracted terms into two sets per patient in the training set: (1) those that were also identified by manual curation of EHR ("Both Manual and NLP"), and (2) those that were identified by NLP but not by manual curation of EHR ("NLP Only").

The goal was to understand how best to filter the NLP-derived terms based on their features, with the assumption that there may be false positives among the terms that were not also identified manually. Therefore, we excluded from this analysis the group of terms that were identified manually but not by NLP. On average, 82% of all manually derived terms were also identified by NLP, indicating that the overlapping terms are a representative sample of all manually identified terms (Figure S1A). Extending the analysis to include ontologically related terms within two steps of each other revealed that only 3.5% of manually extracted terms had no closely related overlapping term in the NLP-derived set, and manual inspection did not identify any particular classes or characteristics of missed terms (Figure S1B).

Next, we computed values for the three-term set features (mean frequency percentile, mean depth, and diversity) for each of the two sets ("Both Manual and NLP" and "NLP Only") per patient. We compared distributions of the two sets, each with 52 values for a given feature such as diversity, using a Wilcoxon's signed-rank test, where the values were paired by patient. The distributions were considered significantly different if the p value was less than 0.01.

Filtering NLP-derived terms

We set thresholds for frequency percentile, depth, and diversity equal to the 5th, 25th, 50th, 75th, and 95th percentiles of the distributions of mean frequency percentile, mean depth, and diversity for the "Both Manual and NLP" sets of HPO terms. For a given patient and threshold per feature, the NLP-derived terms were filtered as follows:

Step 1: calculate frequency percentiles per term and remove all terms below a frequency percentile threshold

Step 2: remove all remaining terms with distances from the HPO root node below a depth threshold

Step 3: calculate mean frequency percentiles for terms grouped by phenotypic abnormality class (note that a term may contribute to multiple), sort abnormality classes by mean frequency percentile in descending order, and select terms belonging to the top N (inclusive) classes, where N is the diversity threshold.

Step 4: if the remaining number of terms is <5, then do not apply any filters.

The choice of filtering by diversity last was due to the impact of prior filtering on sorting the phenotypic abnormality classes by mean frequency percentile.

Performance evaluation criteria

Performance was evaluated using results from the Exomiser variant prioritization tool.[15] Exomiser output was evaluated using seven criteria: (1) the median gene score corresponding to the correctly identified variants, (2) the median rank of the genes containing the correctly identified variants, (3) the minimum number of ranked genes needed to identify all correct diagnostic pathogenic variants, (4) the area under the receiver operating characteristic curve (AUC) and the sensitivity for causal variants to be ranked within the top (5) 5 genes, (6) 10 genes, and (7) 20 genes. Exomiser outputs a variant score based on variant pathogenicity, a phenotype score based on semantic similarity, and a combined score that is a function of the variant and phenotype scores. Exomiser groups variants by gene, assigning each gene the score of its highest-scoring variant (or mean top 2 for compound heterozygotes); the gene score is used to rank the genes. The performance metrics were computed using the gene score and rank (see Supplemental subjects and methods for details).

Ensemble algorithm

For each patient, we averaged the combined Exomiser scores per gene across all 294 combinations of NLP filtering parameters to calculate the ensemble scores. We used the mean combined Exomiser score to rank the genes. The ranks were then used to compute the expected average performance, as described above, of our NLP filtering methods as an ensemble. Note that an ensemble ranking can be determined using maximum votes or average ranking if the mean score is not a reasonable option for a different gene prioritization tool.

Results

Manual phenotyping results in better gene prioritization

We used seven criteria (see Subjects and methods) to compare the diagnostic impact of using manual phenotyping by an expert curator versus automated NLP extraction of phenotypes from the EHR. This comparison was done using Exomiser, a representative variant prioritization tool, on a training set of 52 diagnosed patients. All the genetic data processing parameters for Exomiser were held constant (details in Supplemental subjects and methods).

Exomiser reported the disease-causing variant in 45 of the 52 patients in the training set. The overall performance of Exomiser in correctly identifying the causal gene in 45 patients using manual phenotyping was better than NLP-based phenotyping, with an AUC of 0.85 versus 0.73, respectively (Figure 2A). The greatest difference in sensitivity was seen when considering only the top 5 ranked genes with manual phenotyping (46.7%) being more than twice as sensitive as the NLP-based approach (22.2%). The difference in sensitivity was reduced when considering a larger set of top 10 (73.3% manual versus 51.1% NLP) or top 20 genes (82.2% manual versus 68.9% NLP). Causal genes that were correctly identified when using manual phenotyping were ranked higher than when using NLP phenotyping, with a decrease of 4 in median rank (lower value of rank is better) and a corresponding 0.25 increase in median score; Exomiser scores range between 0 and 1 (Figures 2B and 2C.). However, the rank of the correct causal gene in 10 patients when using NLP-based phenotyping was the same or better than with manual phenotyping (Table S4).

To rule out that underlying characteristics of a patient's genetic disorder impacted which phenotyping method led to better Exomiser performance, we compared distributions of (1) the type of genetic disorder, (2) pathogenicity status of the causal variant, and (3) the variant effect in the group of patients where manual phenotyping resulted in higher gene ranks than NLP-based phenotyping versus the group of patients where it did not. None of these characteristics were enriched in either of the two groups of patients, indicating that these factors did not influence the relative efficiencies of the manual and NLP-enabled approaches (Figure S2). Overall, gene ranking was more correlated with the phenotypic sub-score rather than the variant sub-score (Figures S3 and S4).

We next focused our efforts on post-extraction phenotypic data processing. An obvious difference was that the number of HPO terms extracted by NLP was higher (median number of terms = 340) than the corresponding manual extraction (median number of terms = 15), suggesting the potential for extraneous NLP-derived terms affecting the performance of Exomiser.

Comparing features of NLP-extracted versus manually extracted HPO terms

We looked for differences in features of the HPO terms that were identified by NLP but not by the manual approach, hypothesizing possible enrichment of false-positive and non-specific terms. We suspected that correct terms, as verified by manual curation, are likely to be entered more often in the EHR (have a higher frequency) and that more specific terms have a higher significance in describing the phenotype of the disease. Therefore, we defined two features as proxies for specificity of a set of terms: mean depth and diversity. While depth captured the specificity of the description of a single term relative to its parents in the ontology structure, diversity captured the breadth of phenotypes by counting how many

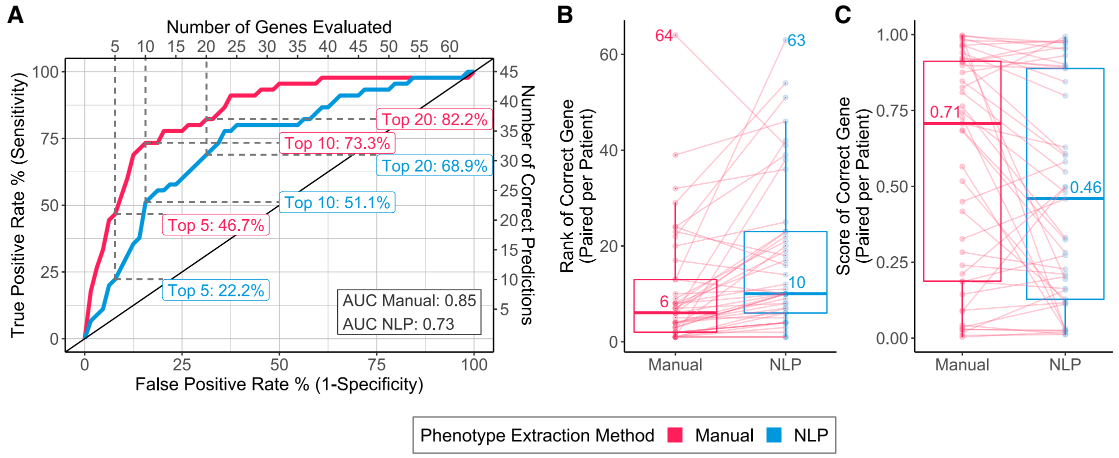

Figure 2. Performance of Exomiser using phenotypes extracted by manual curation versus natural language processing (NLP) among training set cases

(A) Receiver operating characteristic curves with sensitivities noted for specificities corresponding to the top 5, 10, and 20 ranked genes, respectively.

(B) Box and whiskers plots of distribution of the ranks of the correct genes. Each data point in a distribution corresponds to a specific patient, with lines connecting the ranks of each patient across the two phenotype extraction methods to indicate increase versus decrease in rank. The median and max (worst) ranks are also noted adjacent to the corresponding values in the distributions.

(C) Box and whiskers plots of the distribution of the combined Exomiser scores for the correct gene per patient. Each data point in a distribution corresponds to a specific patient, with lines connecting the scores of each patient across the two phenotype extraction methods to indicate increase versus decrease in score. The median scores are noted adjacent to the median values in the distributions.

different phenotypic abnormality branches (classes) of the ontology were represented within a set of terms.

We compared the term frequency as a percentile, depth, and diversity per patient between (1) the set of terms that were identified using both approaches, and (2) the set of terms identified by NLP alone (Figure 3). The mean frequency percentile for terms identified by both approaches was consistently higher (grand mean 75%) than with NLP alone (grand mean 49%). In all patients, there were more HPO terms in the top half of most frequent terms (mean percentile $> 50\%$) that were identified by both approaches. Terms per patient identified manually and by NLP were 0.62 levels deeper on average than terms identified by NLP alone for the same patient. The greatest difference between the pairs of term sets per patient was in diversity, where terms identified by both approaches represented an average of 6.5 different phenotypic abnormality classes versus 22.37 different phenotypic abnormality classes for NLP alone. There are 25 total unique phenotypic abnormality classes within HPO, suggesting that NLP-extracted terms spanned most of the breadth of the ontology, while human curation led to more targeted classes. The difference in distributions between the two sets of terms was significant, with Wilcoxon's signed-rank test p values < 0.01 for all three features, frequency percentile (p value $=$ 5.3E$-$10), mean depth (p value $=$ 6.4E$-$9), and diversity (p value $=$ 3.4E$-$10).

Effect of NLP-extracted term filtering on gene prioritization performance

Given the above feature differences between the HPO term sets, we hypothesized that filtering NLP-extracted terms to more closely resemble terms that had also been identified manually may improve gene prioritization. Since filtering may adversely impact gene prioritization by removing true-positive terms as well, we varied the threshold per term set feature from tolerant to stringent (5–95 percentiles; see Subjects and methods) to filter the list of NLP-extracted terms per patient and evaluated the impact of filtering on Exomiser performance (Figure S5). We explored the 3D performance landscape for all possible combinations of seven different frequency thresholds (0%, 40%, 50%, 60%, 70%, 80%, 90%), six different minimum depth thresholds (0, 4, 5, 6, 7, 8), and seven different diversity thresholds (0, 2, 4, 6, 8, 10, 12) for a total of 294 filter parameter combinations applied to the NLP-extracted HPO terms. We ran Exomiser on the 52 patients in the training set using each of the 294 sets of filtered NLP-extracted HPO terms for a total of 15,288 Exomiser runs and measured performance using the aforementioned criteria (Figure S6; Table S5).

Top-performing filter combinations tended to have a high frequency percentile threshold between 70%–90%, a depth threshold of 6, and diversity thresholds of 6 or higher (Table 1; Table S3). These thresholds more closely resemble expected characteristics of NLP-extracted HPO terms that were also identified manually than NLP-only terms (Figure 3). In the subsequent sections, we refer to an NLP filter combination by its frequency/depth/diversity thresholds (e.g., frequency percentile threshold of 80%, depth threshold of 6 levels, and diversity threshold of 6 abnormality classes is designated as 80/6/6).

NLP filter combinations 80/6/6 and 90/6/6 appeared to be most promising based on our retrospective analysis of

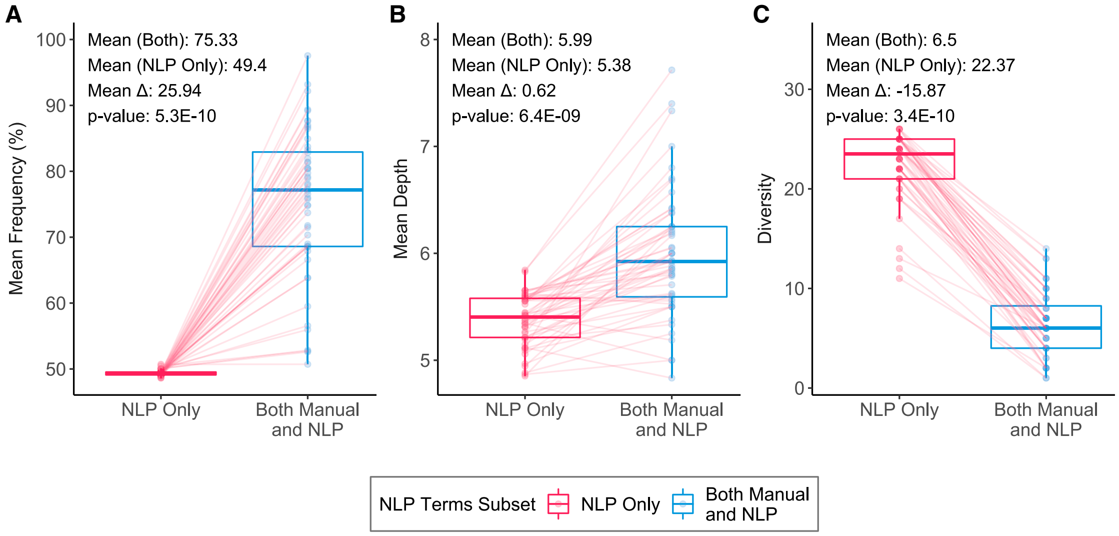

Figure 3. Comparing features of HPO terms identified by NLP alone versus terms identified by both manual- and NLP-based extraction
Box and whiskers plots of (A) distribution of mean frequency percentiles of HPO terms, (B) distribution of mean depth of HPO terms, and (C) distribution of diversity of HPO terms. Each data point in a distribution corresponds to a specific patient in the training set, with lines connecting values of the respective summary feature per patient across the two NLP term subsets to indicate increase versus decrease in value. Mean values per distribition, the difference in means, and associated p-values, calculated using a Wilcoxon's signed-rank test, are noted above each plot.

the training set (Table 1; Table S6). Combination 80/6/6 had the best sensitivity for causal variants to be ranked within the top 5 genes (51.1%, which was superior to manual's 46.7%) and median rank (5, which was superior to manual's 6), while 90/6/6 had the best sensitivity for inclusion in the top 20 genes (91.1%, which was superior to manual's 82.2%). Finally, we found that the average NLP filter ("ensemble") was a better choice than not applying any filter (unfiltered NLP) across all performance metrics and was superior to manual phenotyping in terms of the number of genes needed (49 versus 64 genes) to identify causal genes for all 45 patients and consequently the sensitivity for inclusion in the top 50 genes (100% versus 97.8%) (Figure S7).

Optimizing diagnostic efficiency through a tiered approach to filtering NLP-extracted HPO terms

Our primary motivation is to minimize the number of variants for a clinician or expert to manually evaluate. We constructed a tiered approach to prioritizing genes using NLP-based phenotype extraction that incrementally increases the number of genes/variants to consider as the true diagnosis (Figure S8). Based on the previous analysis (Table 1), we ran gene prioritization with NLP terms filtered using 80/6/6 thresholds as our first tier (step 1) where we examined only the variants within the first 5 ranked genes, which reflects our ideal and most efficient outcome. If none of those variants were considered for further evaluation, the second tier (step 2) was to run gene/variant prioritization with NLP terms filtered using 90/6/6 and evaluating variants within the top 20 genes. If none of the variants in the top 20 genes were considered for further evaluation, we ran all combinations of NLP filters and ranked the genes by the average score (ensemble, step 3). Here, we would consider variants within the top 50 genes, representing the practical limit at which we assessed the case as having causal variants that were either not identifiable or represented in the data or that the NLP may have not captured relevant phenotypes. Finally, if none of the top 50 genes were considered for follow-up, the last tier would be to manually review the medical record and revert to evaluating all variants using that gold standard for HPO curation.

Applying an NLP-extracted phenotype filtering strategy on prospective cases

As expected, optimizing our approach in this way led to improved performance in the training set. To evaluate the utility of this approach in the real world, we applied this NLP filtering method on 12 additional genetically diagnosed patients, referred to as the test set. The 12 cases were subsequently ascertained using the same criteria and data processing workflows as those in the training set (Figure 1). By following the tiered filtration protocol, Exomiser was able to detect the correct causal gene in all 12 cases. Of the 12 cases, the correct causal variant for one patient was identified in step 1, for seven additional patients in step 2, for three additional patients in step 3, and for the one remaining patient in step 4 (Table 2). Overall, NLP-based extraction of HPO terms was a sufficient replacement for manual extraction in 11 out of 12 (92%) cases. The sensitivity within the top 50 genes when using manual phenotyping was also 92% (Figure S9).

We also compared the above tiered pipeline results with results using the unfiltered NLP-extracted HPO terms.

Table 1. Parameter combinations for the top-performing natural language processing (NLP) filters

	Best NLP Filter		
Filtering criteria for top combinations	Frequency (%)	Depth	Diversity
AUC	90	6	6
Median rank	80	6	6
Median score	90	0	12
Genes needed	60	4	10
Sensitivity top 5	80	6	6
Sensitivity top 10	90	6	4
Sensitivity top 20	90	6	6
Median (median absolute deviation)	**90 (0)**	**6 (0)**	**6 (0)**

Compared to 92% sensitivity in the top 50 genes when using our tiered pipeline, unfiltered NLP phenotyping was a sufficient replacement for manual phenotyping in only 75% of prospective cases (Table 2). In 9 of the 12 cases (75%), the gene with the correct variant was ranked higher with an applied filter (including the ensemble) than without any filters (Table S7); the gene ranks were tied in the remaining three cases. In one case (MAN_0842) NLP filtering led to a 23-rank improvement, with the correct variant being ranked in the top 10 (ranked 6) genes as opposed to falling out of the top 20 genes (ranked 29) without any NLP filtering. In two other cases (MAN_1845 and MAN_0805), the correct variant would have been ranked out of the top 50 genes if the NLP terms were not filtered. These results indicate that beneficial NLP term filters can be applied to new patients to improve gene prioritization results. While out of the scope of this work, the diagnostic sensitivities achieved with NLP filtering could be further improved by optimizing Exomiser parameters or using other gene prioritization tools.

Enrichment of manually identified phenotypes after filtering NLP-extracted terms

Our NLP filtering approach was designed to select HPO terms that more closely resemble manual terms with respect to frequency, depth, and diversity. In doing so, we enriched for phenotypes that manual curators selected to characterize each patient's disease (Figure S10A). On average, 4% of unfiltered NLP terms were also identified manually, which coincides with the proportion of the average number of manual terms (14.6) versus NLP terms (355.4). However, after applying the filters in steps 1 and 2 of our tiered pipeline, the average percentage of manual terms in the remaining set of NLP terms increased to 17% (of an average 19.9 terms) and 23% (of an average 12.7 terms), respectively. Moreover, of the NLP terms that did not exactly match a manually identified term, the percentage of closely related terms (defined as having an undirected path length ≤ 2 in the ontology) increased from 13% in the unfiltered NLP lists to 32% and 34% on average after applying step 1 and 2 NLP filters, respectively (Figure S10B). Similarly, the average percentage of unmatched NLP terms that belonged to the phenotypic abnormality classes represented in the manual terms also increased from 57% in the unfiltered NLP lists to 80% and 85% after the step 1 and 2 filters, respectively (Figure S10C). The increased proportion of manually extracted and related terms in the filtered NLP lists indicates that our approach achieved the desired reduction of extraneous terms, a better characterization of the disease phenotype, and the consequent improvement in gene prioritization performance.

Overview of modular software architecture

Our tiered pipeline, running one patient or many patients at a time, requires batch processing of multiple VCF-HPO file combinations, especially when running the ensemble algorithm in step 3. This approach is intended to be applicable to many different diagnostic settings and computational environments; therefore, it was imperative that we implemented a replicable and scalable framework that could batch process many VCF-HPO combinations in parallel. To achieve this, we implemented a batch-processing system that ran Exomiser within a docker container on the Amazon Web Services (AWS) cloud with all input data and results stored on AWS simple storage service (S3) and computed using their elastic compute cloud (EC2) (see Figure S11 and Supplemental subjects and methods).

Discussion

The patient cohort employed for this study represents the most challenging types of cases encountered in a clinical environment. Subjects were enrolled into the Manton Center Gene Discovery Core after extensive clinical evaluation and diagnostic sequencing, including gene panel testing and/or ES that were deemed negative. While ES is increasingly being used as a first-tier diagnostic tool, the infrastructure and funding needed for reanalysis of ES-negative cases is lacking in most clinical and research

Table 2. Sensitivity in prospectively analyzed test set cases comparing NLP filters from the pipeline versus using unfiltered NLP

Pipeline step	Using pipeline NLP filters (n, cumulative %)	Using unfiltered NLP (n, cumulative %)
Step 1: top 5 genes (pipeline uses 80/6/6 filter)	1 (9.09)	1 (9.09)
Step 2: top 20 genes (pipeline uses 90/6/6 filter)	8 (66.67)	7 (58.33)
Step 3: top 50 genes (pipeline uses filter ensemble)	11 (91.67)	9 (75.00)
Step 4: all genes (pipeline uses manual phenotyping)	12 (100)	12 (100)

settings. Furthermore, the expertise and time needed to manually phenotype individuals who often undergo extensive evaluations over long periods of time with complex and large medical charts can be challenging. The use of NLP to extract phenotypic information can overcome this issue. However, a drawback of this automated approach is the relatively high numbers of false-positive and non-specific repetitive terms compared with results of more laborious manual curation. In this paper, we describe the creation and implementation of an automated, reproducible filtering technique that can be applied across health care systems and computing environments to enable the utilization of NLP-extracted terms as an effective substitute for manually extracted HPO terms.

We scanned a three-dimensional feature space of NLP-derived HPO terms—each feature displaying significant variability between NLP-extracted versus manually extracted terms—for filter parameter combinations that optimized gene/variant prioritization. We incorporated the optimal parameter combinations within a tiered filter pipeline that resulted in an outcome comparable to or better than manually curated terms when applied to an independent test set. While previous work[12] has evaluated similar features such as term frequency and proxies for term specificity such as information content, this is the first effort, to the best of our knowledge, to consider combinations of parameters. Furthermore, our approach does not rely on third-party datasets such as STRIDE,[18] facilitating integration with different NLP extractors. However, future work that integrates more sophisticated measures of term specificity, such as information content and weighted paths, as well as ensembles of gene prioritization and NLP extraction algorithms, may improve our filtration approach. A continuing challenge with rare disease data analysis is the limited size of available patient datasets. We are encouraged by the consistency between the results in the training and test sets in terms of overall performance as well as patterns in the underlying metrics. Nevertheless, future studies ought to be expanded to larger datasets for learning filter parameters and out-of-sample testing in larger cohorts.

We considered that other institutions may choose to use different computing environments and aim to modularize their software architecture with substitutable components (Figure S11). The key modules in our architecture are (1) the NLP engine for HPO term extraction, (2) the gene prioritizer, and (3) the batch-processing engine, for which we used Clinithink's CLiX Focus, Exomiser, and parallel processing using Ray[19] on a single AWS EC2 instance, respectively. Multiple options are available for each of these modules and can readily replace our choices (see Supplemental information). We expect that cohorts, EHRs, and consequently optimal combinations of filter parameters will vary by applications and institutions. However, the framework of learning filter parameters from a training set of approximately 50 patients, where HPO terms are extracted manually as well as using NLP, is generally applicable.

Within the context of the Manton Center's Gene Discovery Core, much greater effort is given to manual curation and selection of HPO terms than is normally available in a clinical diagnostic setting. Indeed, the depth and quality of phenotypic data typically available to clinical DNA diagnostic services are notoriously poor, leading to missed diagnoses. The rigorous use of appropriately filtered NLP-based phenotyping has the potential to significantly improve the efficiency of the diagnostic process by limiting the numbers of genes and variants that analysts and clinicians will need to consider before reviewing what ultimately may be determined to represent the causative genetic variant for patients with rare genetic diseases. Such an approach should have similar benefits in both a routine first-pass clinical diagnostic setting, as well as for clinical and research-based reanalysis programs where automated updating from more recently acquired clinical information may provide critical new data to enable a diagnosis.

Data and code availability

Variant interpretations for causal variants are deposited in ClinVar. Additional data are available upon request from qualified investigators. Code is available at https://github.com/alxndgb/pheno_manuscript_Manton_ALXN.

Supplemental information

Supplemental information can be found online at https://doi.org/10.1016/j.xhgg.2021.100035.

Acknowledgments

Alexion would like to acknowledge the AWS Digital innovation team and Slalom, who helped to refine our approach to address

the diagnostic odyssey for all children with rare diseases, which led us to this research project. The authors thank Mohamad Daniar and James Gregoric from the Clinical Research Informatics Team at BCH who helped integrate clinical notes from the CERNER EHR. Support for this study was provided by Alexion Pharmaceuticals, Inc. The mission and activities of The Manton Center for Orphan Disease Research at Boston Children's Hospital are supported and enabled by a visionary gift from the Manton Foundation. Sanger sequencing to confirm causal variants was performed by the Boston Children's Hospital IDDRC Molecular Genetics Core Facility funded by U54HD090255 from the US National Institutes of Health.

Declaration of interests

J.R.P. is the owner and founder of J Square Labs LLC. J.R.P, T.D., P.M., J.R., and S.L. are current or former employees or consultants of Alexion Pharmaceuticals, Inc., and C.Y., R.G., T.W., M.M., A.B., and A.F. are current or former employees of Clinithink Ltd. J.R.P. has consulted for and received compensation from GNS Healthcare and TCB Analytics. J.R. is the owner and founder of Latent Strategies, LLC. A.H.B. has received funding from the NIH, MDA (USA), AFM Telethon, Alexion Pharmaceuticals, Inc., Audentes Therapeutics Inc., Dynacure SAS, and Pfizer Inc. He has consulted and received compensation or honoraria from Asklepios BioPharmaceutical, Inc., Audentes Therapeutics, Biogen, F. Hoffman-La Roche AG, GLG, Inc., Guidepoint Global, and Kate Therapeutics and holds equity in Ballard Biologics and Kate Therapeutics. P.B.A. is on the Clinical Advisory Board of Illumina Inc. and GeneDx. C.A.B. has consulted for, and received compensation or honoraria from, Q State Biosciences. All other authors declare no competing interests.

Received: January 29, 2021
Accepted: May 6, 2021

Web resources

Docker, https://www.docker.com/
Exomiser, http://exomiser.github.io/Exomiser/
Human Phenotype Ontology, https://hpo.jax.org/app/
Manton_ALXN code, https://github.com/alxndgb/pheno_manuscript_Manton_ALXN
OMIM, https://omim.org/
OMIM Gene Map, https://omim.org/statistics/geneMap
Ray, https://ray.io/

References

1. Srivastava, S., Love-Nichols, J.A., Dies, K.A., Ledbetter, D.H., Martin, C.L., Chung, W.K., Firth, H.V., Frazier, T., Hansen, R.L., Prock, L., et al.; NDD Exome Scoping Review Work Group (2019). Meta-analysis and multidisciplinary consensus statement: exome sequencing is a first-tier clinical diagnostic test for individuals with neurodevelopmental disorders. Genet. Med. *21*, 2413–2421.
2. Retterer, K., Juusola, J., Cho, M.T., Vitazka, P., Millan, F., Gibellini, F., Vertino-Bell, A., Smaoui, N., Neidich, J., Monaghan, K.G., et al. (2016). Clinical application of whole-exome sequencing across clinical indications. Genet. Med. *18*, 696–704.
3. Posey, J.E., O'Donnell-Luria, A.H., Chong, J.X., Harel, T., Jhangiani, S.N., Coban Akdemir, Z.H., Buyske, S., Pehlivan, D., Carvalho, C.M.B., Baxter, S., et al.; Centers for Mendelian Genomics (2019). Insights into genetics, human biology and disease gleaned from family based genomic studies. Genet. Med. *21*, 798–812.
4. Dragojlovic, N., Elliott, A.M., Adam, S., van Karnebeek, C., Lehman, A., Mwenifumbo, J.C., Nelson, T.N., du Souich, C., Friedman, J.M., and Lynd, L.D. (2018). The cost and diagnostic yield of exome sequencing for children with suspected genetic disorders: a benchmarking study. Genet. Med. *20*, 1013–1021.
5. Trujillano, D., Bertoli-Avella, A.M., Kumar Kandaswamy, K., Weiss, M.E., Köster, J., Marais, A., Paknia, O., Schröder, R., Garcia-Aznar, J.M., Werber, M., et al. (2017). Clinical exome sequencing: results from 2819 samples reflecting 1000 families. Eur. J. Hum. Genet. *25*, 176–182.
6. Amberger, J.S., Bocchini, C.A., Schiettecatte, F., Scott, A.F., and Hamosh, A. (2015). OMIM.org: Online Mendelian Inheritance in Man (OMIM®), an online catalog of human genes and genetic disorders. Nucleic Acids Res. *43*, D789–D798.
7. Liu, P., Meng, L., Normand, E.A., Xia, F., Song, X., Ghazi, A., Rosenfeld, J., Magoulas, P.L., Braxton, A., Ward, P., et al. (2019). Reanalysis of Clinical Exome Sequencing Data. N. Engl. J. Med. *380*, 2478–2480.
8. Schmitz-Abe, K., Li, Q., Rosen, S.M., Nori, N., Madden, J.A., Genetti, C.A., Wojcik, M.H., Ponnaluri, S., Gubbels, C.S., Picker, J.D., et al. (2019). Unique bioinformatic approach and comprehensive reanalysis improve diagnostic yield of clinical exomes. Eur. J. Hum. Genet. *27*, 1398–1405.
9. Shashi, V., Schoch, K., Spillmann, R., Cope, H., Tan, Q.K.G., Walley, N., Pena, L., McConkie-Rosell, A., Jiang, Y.H., Stong, N., et al.; Undiagnosed Diseases Network (2019). A comprehensive iterative approach is highly effective in diagnosing individuals who are exome negative. Genet. Med. *21*, 161–172.
10. Wenger, A.M., Guturu, H., Bernstein, J.A., and Bejerano, G. (2017). Systematic reanalysis of clinical exome data yields additional diagnoses: implications for providers. Genet. Med. *19*, 209–214.
11. Son, J.H., Xie, G., Yuan, C., Ena, L., Li, Z., Goldstein, A., Huang, L., Wang, L., Shen, F., Liu, H., et al. (2018). Deep Phenotyping on Electronic Health Records Facilitates Genetic Diagnosis by Clinical Exomes. Am. J. Hum. Genet. *103*, 58–73.
12. Deisseroth, C.A., Birgmeier, J., Bodle, E.E., Kohler, J.N., Matalon, D.R., Nazarenko, Y., Genetti, C.A., Brownstein, C.A., Schmitz-Abe, K., Schoch, K., et al.; Undiagnosed Diseases Network (2019). ClinPhen extracts and prioritizes patient phenotypes directly from medical records to expedite genetic disease diagnosis. Genet. Med. *21*, 1585–1593.
13. Köhler, S., Vasilevsky, N.A., Engelstad, M., Foster, E., McMurry, J., Aymé, S., Baynam, G., Bello, S.M., Boerkoel, C.F., Boycott, K.M., et al. (2017). The human phenotype ontology in 2017. Nucleic Acids Res. *45* (D1), D865–D876.
14. Clark, M.M., Hildreth, A., Batalov, S., Ding, Y., Chowdhury, S., Watkins, K., Ellsworth, K., Camp, B., Kint, C.I., Yacoubian, C., et al. (2019). Diagnosis of genetic diseases in seriously ill children by rapid whole-genome sequencing and automated phenotyping and interpretation. Sci. Transl. Med. *11*, eaat6177.
15. Robinson, P.N., Köhler, S., Oellrich, A., Wang, K., Mungall, C.J., Lewis, S.E., Washington, N., Bauer, S., Seelow, D., Krawitz, P., et al.; Sanger Mouse Genetics Project (2014). Improved exome prioritization of disease genes through cross-species phenotype comparison. Genome Res. *24*, 340–348.

16. Rockowitz, S., LeCompte, N., Carmack, M., Quitadamo, A., Wang, L., Park, M., Knight, D., Sexton, E., Smith, L., Sheidley, B., et al. (2020). Children's rare disease cohorts: an integrative research and clinical genomics initiative. NPJ Genom. Med. *5*, 29.

17. Girdea, M., Dumitriu, S., Fiume, M., Bowdin, S., Boycott, K.M., Chénier, S., Chitayat, D., Faghfoury, H., Meyn, M.S., Ray, P.N., et al. (2013). PhenoTips: patient phenotyping software for clinical and research use. Hum. Mutat. *34*, 1057–1065.

18. Lowe, H.J., Ferris, T.A., Hernandez, P.M., and Weber, S.C. (2009). STRIDE–An integrated standards-based translational research informatics platform. AMIA Annu. Symp. Proc. *2009*, 391–395.

19. Moritz, P., Nishihara, R., Wang, S., Tumanov, A., Liaw, R., Liang, E., Elibol, M., Yang, Z., Paul, W., Jordan, M.I., et al. (2017). Ray: A Distributed Framework for Emerging AI Applications. In Proceedings of the 13th USENIX Symposium on Operating Systems Design and Implementation, OSDI 2018 (USENIX Association), pp. 561–577.

HGGA, Volume *2*

Supplemental information

A data-driven architecture using natural language processing to improve phenotyping efficiency and accelerate genetic diagnoses of rare disorders

Jignesh R. Parikh, Casie A. Genetti, Asli Aykanat, Catherine A. Brownstein, Klaus Schmitz-Abe, Morgan Danowski, Andrew Quitadomo, Jill A. Madden, Calum Yacoubian, Richard Gain, Tessa Williams, Mary Meskell, Andrew Brown, Alison Frith, Shira Rockowitz, Piotr Sliz, Pankaj B. Agrawal, Thomas Defay, Paul McDonagh, John Reynders, Sebastien Lefebvre, and Alan H. Beggs

SUPPLEMENTAL METHODS

Study population........ 3
Sequencing data........ 3
HPO description........ 3
Clinithink CliX overview........ 4
NLP term features........ 7
Gene prioritization........ 7
Computing infrastructure........ 8
Performance evaluation criteria........ 8
ROC calculation example........ 9
Issues with automated gene/variant prioritization........ 9
Effect of filtering based on each feature separately........ 10
Optimal combination of parameters........ 10
Overview of modular software architecture........ 11
Cromwell implementation challenges........ 12

SUPPLEMENTAL REFERENCES........ 13

SUPPLEMENTAL FIGURES

Figure S1........ 15
Figure S2........ 16
Figure S3........ 17
Figure S4........ 18
Figure S5........ 19
Figure S6........ 20
Figure S7........ 22
Figure S8........ 23
Figure S9........ 24
Figure S10........ 25
Figure S11........ 26
Figure S12........ 27

SUPPLEMENTAL TABLES

Table S1........ 28
Table S2........ 29
Table S3........ 30
Table S4........ 31
Table S5........ 32
Table S6........ 33
Table S7........ 34
Table S8........ 35
Table S9........ 36
Table S10........ 37
Table S11........ 38

SUPPLEMENTAL METHODS

Study population

The patient cohort employed for this study represents the most challenging cases encountered in a clinical environment, where the readily diagnosable cases of genetic disease have already been solved through targeted testing, thus not requiring further investigation as described here. Typical patients referred to The Manton Center have undergone detailed phenotyping by geneticists and other clinical domain experts as well as had targeted gene or panel testing or clinical ES that was negative or inconclusive leading to reanalysis of their sequenced data[1]. Aside from these subjects having rare or atypical clinical presentations, salient or defining clinical features may be obscured by the use of very general descriptive terms such as "developmental delay", "hypotonia", etc., making detailed review of the clinical record critical to accurate and effective phenotyping. As many disease-gene relationships have only recently been identified, the reported phenotypic spectrums of these conditions are based on small numbers of cases and a number of patients in this cohort represented phenotypic expansions, making accurate and comprehensive phenotyping ever more critical to identify overlapping features with reported clinical presentations.

Sequencing data

The source of ES was paired-end short read Illumina-based sequencing derived from either a clinical diagnostic laboratory or research-based sequencing, with a date of sequencing ranging from 2012 to 2020. Raw sequencing data in the form of BAM or FASTq files were reprocessed via a single pipeline, VeXP, described previously[1]. In order to examine the impact of phenotypic information on making the diagnosis, we excluded trio or other family segregation data and analyzed ES results from probands only to avoid the confounding effects of de novo mutations in sporadic cases, identity by descent in consanguineous cases, etc.

HPO description

Human Phenotype Ontology (HPO) is formed of more than 13,000 thousand terms that describe the individual symptoms and signs observed in human diseases. HPO terms provide a standard and organized nomenclature for clinicians and researchers interested in studying phenotype driven data. These terms are determined through the use of different medical resources including Orphanet, DECIPER and OMIM. The HPO project can be accessed through http://www.human-phenotype-ontology.org.

Each term in the HPO describes a specific clinical abnormality and corresponds to a unique 7-digit HPO identifier. For example, Seizure is an HPO term with the uniquid identifier HP: 0001250. Different synonyms of the same clinical abnormalities are combined under one term. In this specific case, epileptic seizure, seizure and epilepsy would all be under the umbrella term Seizure HP: 0001250.

HPO terms are connected to each other depending on their hierarchal relationship. The first parent terms are formed of the most generalized and nonspecific clinical abnormalities while the more distal branches include more specific descriptions. This specificity is achieved through different subontologies describing the inheritance patterns, onset, localization, course and frequency of clinical manifestations. The distal branches of HPO terms inherit their parent terms all the way up to the root.

For example, Abnormality of the nervous system, HP: 0000707 is the root term that encompasses a variety of different clinical abnormalities pertaining to the nervous system. Its distal branches include Seizure HP: 0001250 which itself has numerous subclasses depending on its localization, onset and characteristics. One of its most distal branches includes Bilateral tonic-clonic seizure with focal onset, HP: 0007334, which is a very specific. Both of these terms inherit their more proximal parent terms all the way up to the most general root.

Clinthink CliX overview

Clinithink's patented Clinical Natural Language Processing (CNLP) engine, CLiX, has been developed over more than a decade of R&D. CLiX uses a combination of a statistical, ontological and linguistic approaches that have been refined and optimized specifically for clinical narrative found in physician-authored documentation. As part of the optimization process, a large training set of almost 10 billion words was utilized. CLiX supports an advanced NLP pipeline that focuses on noun phrases as the highest value sentence component and typically runs over 30 separate processes as part of the pipeline on the input noun phrase. The technology is highly scalable and can execute the entire pipeline in milliseconds on an input sentence. This equates to being able to process over 1 million documents in an hour on approximately $10 worth of AWS resources.

CLiX leverages SNOMED CT extensively as its primary ontology, enabling the computational representation of nearly 2 billion possible expressions commonly found in clinical narrative. Synonyms, acronyms and misspellings have all been extensively modelled and large dictionaries containing these variants continue to be maintained by Clinithink as part of the solution.

In the CNLP document processing workflow, CLiX undertakes heading recognition, segmentation, normalization and tokenization. Documents are first split into "headings" and then the input data is broken down into "segments" (sentences) and "tokens" (individual words or punctuation sequences). Each segment is normalized by converting the input data characters to the standard Latin character set. Tokens are analyzed for spelling errors and are corrected using an algorithm within CLiX. After spelling correction, tokens are analyzed against the metadata files (phrase-set, abbreviations, acronyms and synsets). Based on the content contained in these metadata files and the context file, tokens are replaced according to the content of the metadata files.

Once pre-processing is complete, concept matching begins, CLiX matches tokens to pre-coordinated SNOMED concepts and assembles legitimate fully-modelled post-coordinated expressions based on SNOMED compositional grammar. Input data is then reviewed by CLiX to

identify any elements that may not already be represented in the encoded data, such as quantities, date, time or value information. The information model data is stored with the SNOMED CT concepts to which the data applies. Because CLiX is based on SNOMED-CT, it can be used effectively for projects for any disease area. The core SNOMED-CT content is managed through a quality-based approach using expert personal, standard guidelines and process, and collaboration with key stakeholders and SME groups (including Clinithink). This means that there is a both a broad and deep approach to expanding the terminology so that it can be utilized for any clinical use case. The CLiX Engine is also customizable to be able to interpret how language/dialect is written locally (acronyms, synonyms, abbreviations, etc.) as well as expandable based on our SNOMED extension. This is important because innovative drugs, new tests, and new therapies are constantly being introduced to improve clinical care and outcomes.

After the CLiX Engine has encoded all clinical content found in the unstructured text to post-coordinated SNOMED, a key and unique part of our solution is a query capability to extract the phenotypes for each patient. For CLiX focus (Clinithink's deep phenotyping solution) an HPO Queryset has been created to output the relevant HPO codes and concepts for each patient based on the underlying SNOMED expressions. Clinithink's HPO Queryset is not a mapping of SNOMED to HPO, but a thorough representation of the full HPO concept using one or more SNOMED expressions, with the output being the representative HPO code and concepts for consumption by downstream systems. The HPO Queryset version 11.2 was used for this project. The development of this Queryset involved the following process:

1. Synonyms identified as "layperson" were deferred and removed from the target. Synonyms that were clinical and therefore more likely to be used in a patient's record were prioritized and none of these were deferred.
2. Concepts that were created for the sole purpose of hierarchal grouping were deferred. These were concepts that begin with "Abnormal" or "Abnormality of" and were not phrases that a clinician would use to describe a clinical feature. Each of these had children concepts/phenotypes that were better defined and attributed.
3. The remaining phenotypes and synonyms were processed against CLiX to determine the encoding coverage. The list was reviewed automatically as part of proprietary Clinithink workflow tools and manually as needed by clinical terminologists.
4. Those that weren't encoded or didn't have all the phenotypes attributes fully represented were put into a production workflow and prioritized based on relationships to other rare disease ontologies (OMIM & Orphanet) and population prevalence taken from source files.
    a. Typical encoding & Queryset representation issues included:
        - missing adjectives/attributes
        - foreign characters
        - unusual punctuation
        - missing sub-types
        - ambiguous acronyms
    b. CLiX configuration or Queryset development typically involves:
        - Adding or modifying CNLP configuration rules

- Adding to the Clinithink SNOMED extension to fully model the concept
- Adding logic to join multiple SNOMED expressions together in order to fully represent a single HPO concept

The CLiX Engine encodes negation as 'Known Absent' in the post-coordinate SNOMED Expression. However, the HPO Queryset does not currently leverage 'Known Absent' encodings.

An important part of any CLiX project is identifying the source clinical narrative and making sure that those documents are loaded with the highest quality, fidelity and integrity. Part of this process is classifying the types of narrative in the EHR or other source systems based on:

a. Clinical significance and relevance
b. Data formatting (is the document truly narrative or just structured values that may not be properly or fully interpreted due to imbedded tables from template-based document types)
c. Identifying patient instruction documents or other similar types that may be generic, informational and not specific to the patient or their encounter.

As part of this project, BCH provided a list of all the document types in their CERNER EHR system for review by the project team; CLiX does not natively ingest PDFs. This initial review was done collaboratively from members of the BCH and Clinithink teams and resulted in a list of 462 document types being flagged for importing into CLiX. (see Supplemental Table 6 in Rockowitz *et al*, 2020[3] for full list)

Those document types for the project cohort were extracted by BCH into a SQL staging table. The data was reviewed as like any standard CLiX implementation project to see if there were any pre-loading data transformations/standardizations that needed to happen to improve data quality and eliminate data loading errors. The CLiX Engine reads and interprets clinical narrative like a human does, therefore formatting of the document is key to accurate encoding.

Narrative text may often go through several integration pipelines and be transformed from several formats. This can result in artifacts (HTML, RTF, XML, HL7, etc.) being unintentionally embedded within the document itself. This can also include document headers and footers that disrupt the narrative flow of a document. These need to be removed or transformed prior to import into CLiX.

In addition, templates are commonly used within all modern EHRs, and although they can improve the clinician's user experience, they can leave characters, spacing, tables or other data flaws within the documents that any NLP product (or human) will struggle with accurately interpreting. Parts of these are identified during the initial data assessment process while the data is still in the staging database table, while others are identified using the CLiX engine itself. This may include identify new headers that were not included in the standard CLiX configuration, headers that need to be ignored because the subsequent data is not relevant to the patient, or other configuration needed to process BCH data – regardless of the use case. In other words, this configuration is not for customizing the CLiX engine for content, as much as it

is tuning the configuration based on the formatting of the documents. The templates and documents that BCH uses in their EHR system are not the same as other hospitals – even if it is the same CERNER system. For this project, the clinical documents for each of the patients in the study cohort were imported into CLiX focus and processed against the HPO Queryset v11.2.

NLP term features

We computed the following values per patient for a given set of NLP-extracted HPO terms: 1) mean frequency percentile, 2) mean depth, and 3) diversity.

Frequency percentile was calculated using the ranks of all HPO terms for a given patient based on term frequency; tied ranks were averaged. Mean frequency percentile was computed as the arithmetic mean of the frequency percentiles of all HPO terms within a given set.

Depth and phenotypic abnormality classes were determined using the HPO structure. Depth was calculated as the distance of the shortest directed path from the root node in the HPO ontology to the respective term using an unweighted breadth-first search. Mean depth was computed as the arithmetic mean of the depth of all HPO terms within a given set.

A term may have multiple shortest paths from the root node, by definition, with the same shortest distance. A term was assigned all unique phenotypic abnormality classes that its shortest paths passed through (Table S3; a list of phenotypic abnormalities and details on each HPO term can be found at http://www.informatics.jax.org/vocab/hp_ontology/HP:0000118). Terms that were at the same hierarchy level as a phenotypic abnormality class node or above were not assigned any class. We defined diversity as the number of unique phenotypic abnormality classes represented within a given set of HPO terms. We utilized the diversity and depth features as a proxy for term specificity in our analysis.

Gene prioritization

The latest version of Exomiser[4] (version 12.1.0 with 2003 versions of both the hg19 genome and phenotype data) was downloaded from https://Exomiser.github.io. Exomiser takes an input VCF file and a list of HPO-encoded phenotypes and ranks potential disease-causing genes by combining variant pathogenicity with semantic similarity between the patient's phenotypes and phenotypes associated with a gene via gene-disease associations from Orphanet[5] and OMIM.[6,7] We used the default parameters except for maxFrequency in the frequencyFilter, which was set to 1%, the phenix prioritizer was used instead of the hiPhive prioritizer, and the output formats were limited to HTML and JSON. All analysis settings are as follows:

genomeAssembly: hg19
inheritanceModes:
{ AUTOSOMAL_DOMINANT: 0.1, AUTOSOMAL_RECESSIVE_HOM_ALT: 0.1, AUTOSOMAL_RECESSIVE_COMP_HET: 2.0, X_DOMINANT: 0.1, X_RECESSIVE_HOM_ALT: 0.1, X_RECESSIVE_COMP_HET: 2.0, MITOCHONDRIAL: 0.2 }
analysisMode: PASS_ONLY

frequencySources:
[THOUSAND_GENOMES, TOPMED, UK10K, ESP_AFRICAN_AMERICAN,
ESP_EUROPEAN_AMERICAN, ESP_ALL, EXAC_AFRICAN_INC_AFRICAN_AMERICAN,
EXAC_AMERICAN, EXAC_SOUTH_ASIAN, EXAC_EAST_ASIAN,EXAC_FINNISH,
EXAC_NON_FINNISH_EUROPEAN, EXAC_OTHER, GNOMAD_E_AFR, GNOMAD_E_AMR,
GNOMAD_E_EAS, GNOMAD_E_FIN, GNOMAD_E_NFE, GNOMAD_E_OTH,
GNOMAD_E_SAS, GNOMAD_G_AFR, GNOMAD_G_AMR, GNOMAD_G_EAS,
GNOMAD_G_FIN, GNOMAD_G_NFE, GNOMAD_G_OTH, GNOMAD_G_SAS]
pathogenicitySources: [POLYPHEN, MUTATION_TASTER, SIFT]
steps: [
variantEffectFilter: {
remove: [
FIVE_PRIME_UTR_EXON_VARIANT, FIVE_PRIME_UTR_INTRON_VARIANT,
THREE_PRIME_UTR_EXON_VARIANT,
THREE_PRIME_UTR_INTRON_VARIANT,
NON_CODING_TRANSCRIPT_EXON_VARIANT,
UPSTREAM_GENE_VARIANT, INTERGENIC_VARIANT,
REGULATORY_REGION_VARIANT,
CODING_TRANSCRIPT_INTRON_VARIANT,
NON_CODING_TRANSCRIPT_INTRON_VARIANT,
DOWNSTREAM_GENE_VARIANT
]
},
frequencyFilter: {maxFrequency: 1.0},
pathogenicityFilter: {keepNonPathogenic: true},
inheritanceFilter: {},
omimPrioritiser: {},
phenixPrioritiser: {}
]

Computing infrastructure

R (version 3.5.2) and Python (version 3.8) were used for all data processing, analysis, and visualization. The ontologyIndex (version 2.5) R package was used for parsing ontology files in OBO format and the igraph (version 1.2.4.1) R package was used for computing shortest paths. The ray[8] (version 0.9) python package was used for parallel processing on AWS EC2. Cromwell[9] server (version 50) was installed on AWS EC2 and configured to deploy tasks using AWS Batch.

Performance evaluation criteria

Exomiser was able to identify the correct causal variant in 45 out of 52 patients in the training set. We computed the median of the gene score corresponding to the correct causal variant, henceforth referred to as the causal gene, across the aforementioned 45 patients as the first performance evaluation criterion. We also noted the rank of the correct causal gene per patient. We computed the mean rank and the lowest rank across all 45 patients as two additional performance evaluation criteria. Next, we computed the AUC as a balance between

the number of causal genes identified correctly and the rank of those genes. We computed the receiver operating curve (ROC) by calculating the sensitivity or true positive rate (TPR) as the percent of causal genes (out of 45) correctly identified for varying gene rank thresholds; the false positive rate (FPR) was calculated as the rank threshold divided by the lowest possible rank (see below for example calculation). The AUC under the TPR vs FPR ROC curve was calculated using the trapezoidal rule. The AUC depends on the lowest possible rank amongst runs to be compared and can vary for the same run if a different set of runs are compared such that the lowest possible rank changes. Therefore, we only used the AUC as a relative measure between pipeline runs. In order to account for this, we also used the sensitivity at specific gene rank thresholds of 5, 10, and 20, considering them as practical limits on the number of genes to evaluate per patient, as additional performance evaluation criteria.

ROC calculation example

The receiver operating characteristic (ROC) curve plots the true positive rate (TPR) at varying thresholds of a false positive rate (FPR). In this work, we computed the TPR as the fraction of patients where the correct causal gene (1 per patient) was predicted and the FPR as the fraction of total genes needed to be evaluated to make all possible correct predictions*, i.e.* an incremental gene rank threshold divided by the lowest possible rank across all patients. The following table shows the example TPR and FPR calculations:

# Genes Evaluated	FPR Formula	FPR	# Correct Predictions	TPR Formula	TPR
1	1 / 64	1.56%	8	8 / 45	17.78%
2	2 / 64	3.13%	12	12 / 45	26.67%
3	3 / 64	4.69%	15	15 / 45	33.33%
4	4 / 64	6.25%	20	20 / 45	44.44%
…					
63	63 / 64	98.44%	44	44 / 45	97.78%
64 (worst ranking gene)	64 / 64	100.00%	45 (total number of patients)	45 / 45	100.00%

Issues with automated gene/variant prioritization

Exomiser was unable to identify the correct causal gene in 7 out of the 52 patients irrespective of the phenotype extraction method. There were several reasons why gene prioritization was insufficient in these cases. In one of the cases, the causal gene would have been identified but the causative variant did not pass the maximum minor allele frequency threshold of 0.1% in one of the reference populations, the European ancestry (EA) cohort from the NHLBI GO Exome Sequencing Project (ESP)[10] by a minute margin (0.109%). In previous versions of the Exomiser phenotype data (v1909; we used v2003, which was the latest available at the time of this work), the variant would have been captured because it passed a ClinVar-based[11] whitelist. Exomiser authors envision a bespoke white list that would augment or supplant their default whitelist. In this work, we did not alter most genomic data processing parameters and default data files in order to limit the variability from non-phenotypic data sources. While out of scope for this study, we recommend further exploration of genomic parameters to focus gene prioritization

of the appropriate set of variants given the characteristic of the patient population to be studied.

Effect of filtering based on each feature separately

We applied frequency percentile, depth, and diversity filters one at a time to understand the effect of each filter on Exomiser performance (Figure S5). Filtering of NLP terms by frequency percentile improved overall performance relative to Exomiser results using unfiltered NLP terms, with AUCs increasing with increased stringency (higher threshold). Filtering by depth also had a positive correlation with stringency on performance for AUC and sensitivity within the top 5 ranking genes. Varying the diversity threshold had the largest impact on overall performance with the AUC and median rank worsening the most with increased stringency (smaller diversity threshold). Filtering of NLP terms by frequency percentile improved overall performance relative to Exomiser results using NLP terms without any filtering (dashed blue line in Figure S5) with AUCs increasing with increased stringency (higher threshold). The positive correlation between frequency percentile threshold and performance was also true for the median score. Sensitivity when considering the top 5 and top 20 ranking genes also improved with frequency filtering, while the sensitivity within the top 10 ranking genes and median rank stayed about the same or worsened slightly than before filtering. Filtering by frequency percentile also led to fewer genes needed to correctly identify the causal gene in all 45 possible patients. Filtering by depth also had a positive correlation with stringency on performance for AUC and sensitivity within the top 5 ranking genes. However, unlike frequency filtering there was an improvement in median rank and sensitivity within the top 10 genes, but a lack of improvement in median score. The number of genes needed was also lower than without filtering in all cases except the most stringent depth threshold of 8 levels deep. Diversity had the largest change in overall performance with the AUC and median rank worsening the most with increased stringency (smaller diversity threshold). The diversity threshold when applied independently of depth or frequency percentile likely led to the removal of important (high frequency) and specific (high depth) HPO terms, which did not belong to the phenotypic abnormality classes that were merely on average more frequently represented in the EHR. Given the complementary behavior in performance metrics between the three filter criteria, we next explored all possible combinations of frequency, depth, and diversity thresholds.

Optimal combination of parameters

In order to develop an approach to maximize the utility of NLP and determine best practices for filtering NLP outputs to bring gene prioritization performance as close to that following expert curation as possible, we explored the performance landscape for all possible combinations of NLP filters. We applied all combinations of 7 different frequency filters (0%, 40%, 50%, 60%, 70%, 80%, 90%), 6 different minimum depth filters (0, 4, 5, 6, 7, 8), and 7 different diversity filters (0, 2, 4, 6, 8, 10, 12) for a total of 294 (7 x 6 x 7) sets of filter parameter combinations on the NLP-extracted HPO terms for the 52 patients with known diagnosis. We ran Exomiser on the 52 patients in the training set using each of the 294 sets of filtered NLP-extracted HPO terms for a total of 15,288 Exomiser runs, and measured performance using 7 different performance criteria described above (Figure S6, Table S5). As the filters become more stringent there are

more patients where the filter combination left too few HPO terms (Table S8). However, 70% of filter combinations (208 out of 293; note 1 is the unfiltered NLP list) reduced the number of HPO terms to more than 5 terms in over 80% of patients (42 out of 52 patients).

Next, we explored the specific best NLP filter option per patient. The promise of knowing the best NLP filter combination for a given patient is that 43/45 (95.6%) of the patients would not need time consuming manual term extraction if that optimal filter combination were known *a priori* (Table S9). In order to decipher the relationship between optimal filter combinations and patients, we clustered the specific best NLP filter option per patient (Figure S12) to identify patterns of patients and filter parameters. We did not observe clear clusters of patients or identify major patterns in filter parameter combinations except for an anticorrelation between frequency percentile and depth filter thresholds where two distinct clusters benefited from either one of the two filters but not both (Table S10).

With sufficiently large sample sizes we may eventually be able to decipher which NLP filters would work best for which patients. However, until then we may still be able to provide a benefit with overall best performing NLP filters (Table S6).

To assess if these 3 NLP filter options would provide a benefit, we compared the rank of the correct gene after NLP filtering to their ranks when using manually extracted and the unfiltered NLP phenotype lists (Table S11). We split the patients into two groups based on the preferred phenotype extraction method if filtering were not an option (Table S4). Ties in rank were broken based on the following preference (Combination Filters [80/6/6 or 90/6/6] > Ensemble > Unfiltered NLP > Manual). 64.4% of patients (29 out of 45) would have at least as good a rank with an NLP filter applied as manual phenotyping. The majority (54.29%) of the patients (19 out of 35) that had a better rank with manual phenotyping than unfiltered NLP-based phenotyping would now have at least as good a rank with an NLP filter. Most of the patients that benefit from an NLP filter see the benefit with one of the specific NLP combination filters over the ensemble. Note that there were zero patients that benefitted from the unfiltered NLP over the filtered options. Combinations 80/6/6 and 90/6/6 would be the preferred choice of NLP filters for about the same number of patients (11 vs 13 respectively) with the correct gene for 4 patients being ranked the same with either filter (Table S11).

Overview of modular software architecture

Our tiered pipeline, run on one patient or many patients at a time, requires batch processing of multiple VCF-HPO file combinations, especially when running the ensemble algorithm in step 3. In one of our most extreme recent analyses, we needed to run 294 filter combinations on 110 patients. A single variant prioritization run, including data management and cleanup, requires approximately 7 minutes. The 32,340 (294 x 110) runs would have taken 157 days to complete if run serially. We intend this approach to be applicable to many different hospitals and computational environments, therefore, it was imperative that we implemented a replicable and scalable framework that could batch process many VCF-HPO combinations in parallel. We implemented a batch processing system in the cloud using Amazon Web Services (AWS) with all input data and results stored on AWS simple storage service (S3) and compute using their

elastic compute cloud (EC2) (Figure S11). The gene/variant prioritizer (Exomiser in our case) was containerized using docker[12] and stored in Docker Hub[13]. Containerization, i.e. encapsulating the repetitively accessed application, enables portability to almost any scalable computing infrastructure. In the simplest deployment, we ran batches of 65 VCF-HPO combinations that called the containerized gene prioritizer on a single memory-optimized EC2 instance that had 512 Gb of memory. The 32,340 runs completed in approximately 2 days and cost approximately $2 per patient and under $250 overall. We also implemented the parallel processing pipeline using Cromwell[9], developed by the Broad Institute, and AWS Batch, but replaced it due to error handling issues (see below). The runtime and costs could have been further reduced by using AWS Batch with spot instances.

We were mindful that other institutions may choose to use different computing environments and aim to modularize the software architecture with substitutable components (Figure S11). The key modules in our architecture are 1) the NLP engine for HPO term extraction, 2) the gene prioritizer, and 3) the batch processing engine, for which we used Clinithink's CLiX Focus, Exomiser, and parallel processing using Ray[8] on a single AWS EC2 instance respectively. NLP engine options outside of Clinthink include CLAMP[14], ClinPhen[15], and cTakes[16]. Exomiser may be substituted with MOON from Diploid[17,18], Extasy[19], Phen-Gen[20], and MutationDistiller[21] and others. As described above, we initially used Cromwell as our batch processing engine, but later replaced it with a custom script running on a single AWS EC2 instance. We intend to further increase efficiency and decrease costs by deploying our parallel processing pipeline on AWS Batch in the future. Other popular options for orchestrating parallel tasks and managing workflows are Luigi[22] and Apache Airflow[23]. Most of the options described above can be implemented on cloud platforms other than AWS as well as on premises.

Cromwell implementation challenges

We initially implemented the parallel processing pipeline using Cromwell[16], developed by the Broad Institute, and AWS Batch. One challenge with parallel processing pipelines that utilize data stored on AWS S3 is handling errors introduced by concurrent file access. These errors can be handled within custom scripts, as we did when implementing the aforementioned single instance-based pipeline. In Cromwell, we set the maxRetries runtime attribute to 3, which reduced the number of pipeline crashes due to S3 read errors. We found the principal advantages of Cromwell to be the reduction in programming required for orchestrating parallel jobs and the availability of an application programming interface (API) for submitting and monitoring jobs. While we did not optimize for cost savings, we found the overall compute cost and runtime to be higher when using Cromwell and that the handling of errors for our largest job was too onerous, and thus used a single on-demand instance for our batch processing pipeline.

SUPPLEMENTAL REFERENCES

1. Schmitz-Abe, K., Li, Q., Rosen, S.M., Nori, N., Madden, J.A.J.A., Genetti, C.A.C.A., Wojcik, M.H.M.H., Ponnaluri, S., Gubbels, C.S.C.S., Picker, J.D.J.D., et al. (2019). Unique bioinformatic approach and comprehensive reanalysis improve diagnostic yield of clinical exomes. Eur. J. Hum. Genet. *27*, 1398–1405.
2. Girdea, M., Dumitriu, S., Fiume, M., Bowdin, S., Boycott, K.M., Chénier, S., Chitayat, D., Faghfoury, H., Meyn, M.S., Ray, P.N., et al. (2013). PhenoTips: Patient phenotyping software for clinical and research use. Hum. Mutat. *34*, 1057–1065.
3. Rockowitz, S., LeCompte, N., Carmack, M., Quitadamo, A., Wang, L., Park, M., Knight, D., Sexton, E., Smith, L., Sheidley, B., et al. (2020). Children's rare disease cohorts: an integrative research and clinical genomics initiative. Npj Genomic Med. *5*, 1–12.
4. Smedley, D., Jacobsen, J.O.B., Jäger, M., Köhler, S., Holtgrewe, M., Schubach, M., Siragusa, E., Zemojtel, T., Buske, O.J., Washington, N.L., et al. (2015). Next-generation diagnostics and disease-gene discovery with the Exomiser. Nat. Protoc. *10*, 2004–2015.
5. Rath, A., Olry, A., Dhombres, F., Brandt, M.M., Urbero, B., and Ayme, S. (2012). Representation of rare diseases in health information systems: The orphanet approach to serve a wide range of end users. Hum. Mutat. *33*, 803–808.
6. Zemojtel, T., Köhler, S., Mackenroth, L., Jäger, M., Hecht, J., Krawitz, P., Graul-Neumann, L., Doelken, S., Ehmke, N., Spielmann, M., et al. (2014). Effective diagnosis of genetic disease by computational phenotype analysis of the disease-associated genome. Sci. Transl. Med. *6*, 252ra123-252ra123.
7. Amberger, J., Bocchini, C., and Hamosh, A. (2011). A new face and new challenges for Online Mendelian Inheritance in Man (OMIM®). Hum. Mutat. *32*, 564–567.
8. Ray – Fast and Simple Distributed Computing. https://ray.io/
9. Home - Cromwell. https://cromwell.readthedocs.io/en/stable/
10. Tennessen, J.A., Bigham, A.W., O'Connor, T.D., Fu, W., Kenny, E.E., Gravel, S., McGee, S., Do, R., Liu, X., Jun, G., et al. (2012). Evolution and functional impact of rare coding variation from deep sequencing of human exomes. Science (80-.). *336*, 64–69.
11. Harrison, S.M., Riggs, E.R., Maglott, D.R., Lee, J.M., Azzariti, D.R., Niehaus, A., Ramos, E.M., Martin, C.L., Landrum, M.J., and Rehm, H.L. (2016). Using ClinVar as a resource to support variant interpretation. Curr. Protoc. Hum. Genet. *2016*, 8.16.1-8.16.23.
12. Empowering App Development for Developers | Docker. https://www.docker.com/
13. Docker Hub. https://hub.docker.com/
14. Soysal, E., Wang, J., Jiang, M., Wu, Y., Pakhomov, S., Liu, H., and Xu, H. (2018). CLAMP - a toolkit for efficiently building customized clinical natural language processing pipelines. J. Am. Med. Informatics Assoc. *25*, 331–336.
15. Deisseroth, C.A., Birgmeier, J., Bodle, E.E., Kohler, J.N., Matalon, D.R., Nazarenko, Y., Genetti, C.A., Brownstein, C.A., Schmitz-Abe, K., Schoch, K., et al. (2019). ClinPhen extracts and prioritizes patient phenotypes directly from medical records to expedite genetic disease diagnosis. Genet. Med. *21*, 1585–1593.
16. Savova, G.K., Masanz, J.J., Ogren, P. V., Zheng, J., Sohn, S., Kipper-Schuler, K.C., and Chute, C.G. (2010). Mayo clinical Text Analysis and Knowledge Extraction System (cTAKES):

Architecture, component evaluation and applications. J. Am. Med. Informatics Assoc. *17*, 507–513.
17. Diploid - Diagnosing Rare Diseases. http://www.diploid.com/moon
18. Clark, M.M., Hildreth, A., Batalov, S., Ding, Y., Chowdhury, S., Watkins, K., Ellsworth, K., Camp, B., Kint, C.I., Yacoubian, C., et al. (2019). Diagnosis of genetic diseases in seriously ill children by rapid whole-genome sequencing and automated phenotyping and interpretation. Sci. Transl. Med. *11*,
19. Sifrim, A., Popovic, D., Tranchevent, L.C., Ardeshirdavani, A., Sakai, R., Konings, P., Vermeesch, J.R., Aerts, J., De Moor, B., and Moreau, Y. (2013). EXtasy: Variant prioritization by genomic data fusion. Nat. Methods *10*, 1083–1086.
20. Javed, A., Agrawal, S., and Ng, P.C. (2014). Phen-gen: Combining phenotype and genotype to analyze rare disorders. Nat. Methods *11*, 935–937.
21. Hombach, D., Schuelke, M., Knierim, E., Ehmke, N., Schwarz, J.M., Fischer-Zirnsak, B., and Seelow, D. (2019). MutationDistiller: User-Driven Identification of Pathogenic DNA Variants. Nucleic Acids Res. *47*, W114–W120.
22. Luigi. https://luigi.readthedocs.io/en/stable/
23. Apache Airflow. https://airflow.apache.org/
24. Best, D.J., and Roberts, D.E. (1975). Algorithm AS 89: The Upper Tail Probabilities of Spearman's Rho. Appl. Stat. *24*, 377.

SUPPLEMENTAL FIGURES

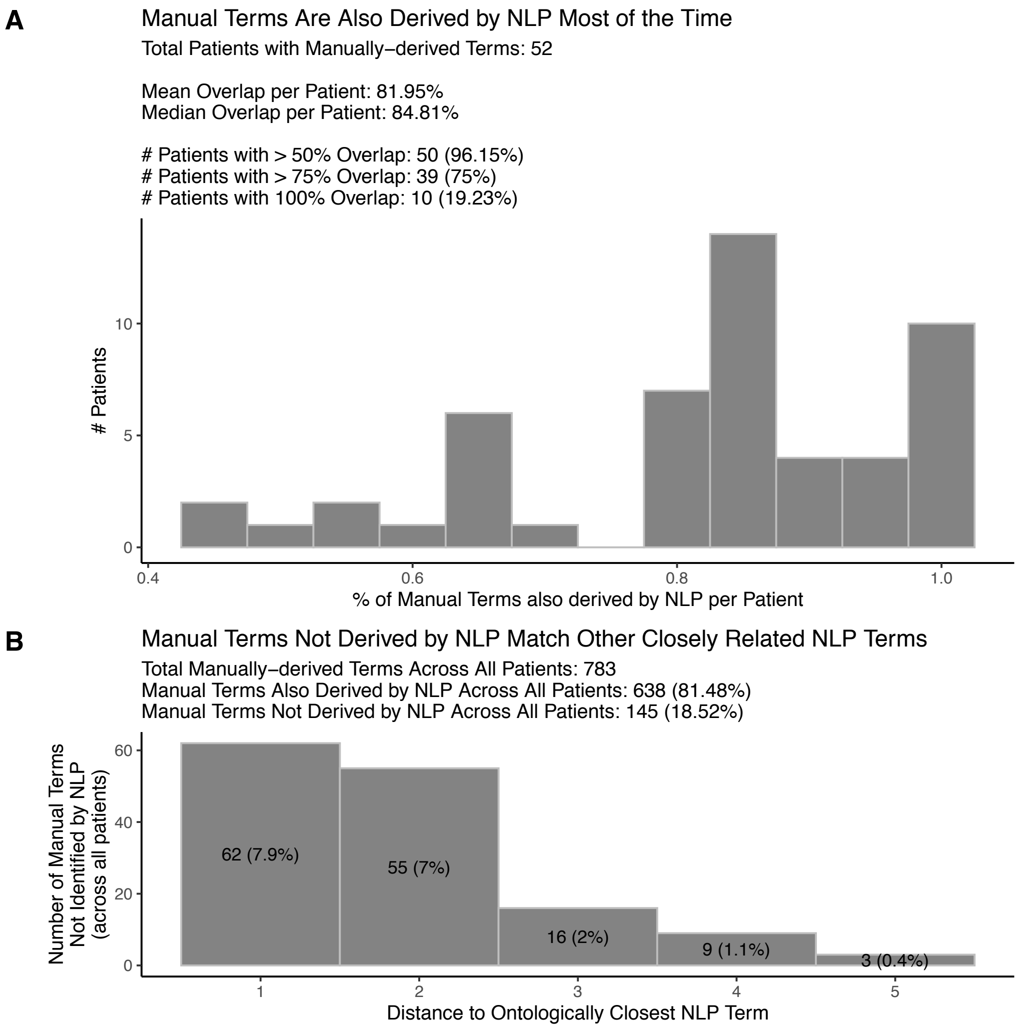

Figure S1. Overlap between manually-extracted terms and NLP-extracted terms per patient
(A) We computed the intersection between the set of terms extracted using manual curation with those terms extracted using NLP per patient as a percentage of the number of manually-extracted terms. On average, 81.95% of manually-extracted terms were also identified by NLP. The median percentage of overlapping terms was higher at 85%. 51/52 (96%) of the patients had at least half the manually-extracted terms identified via NLP.
(B) We searched for related NLP terms to those manual terms not identified by NLP. We calculated the undirected distance between the nearest NLP term in the HPO directed acyclic graph (DAG) to each of the 146 manual terms not also identified by NLP (18.5% of all terms). Only 28 manual terms (3.5% of all terms) did not have a close ontologically related (within 2 steps) term in the NLP-derived list.

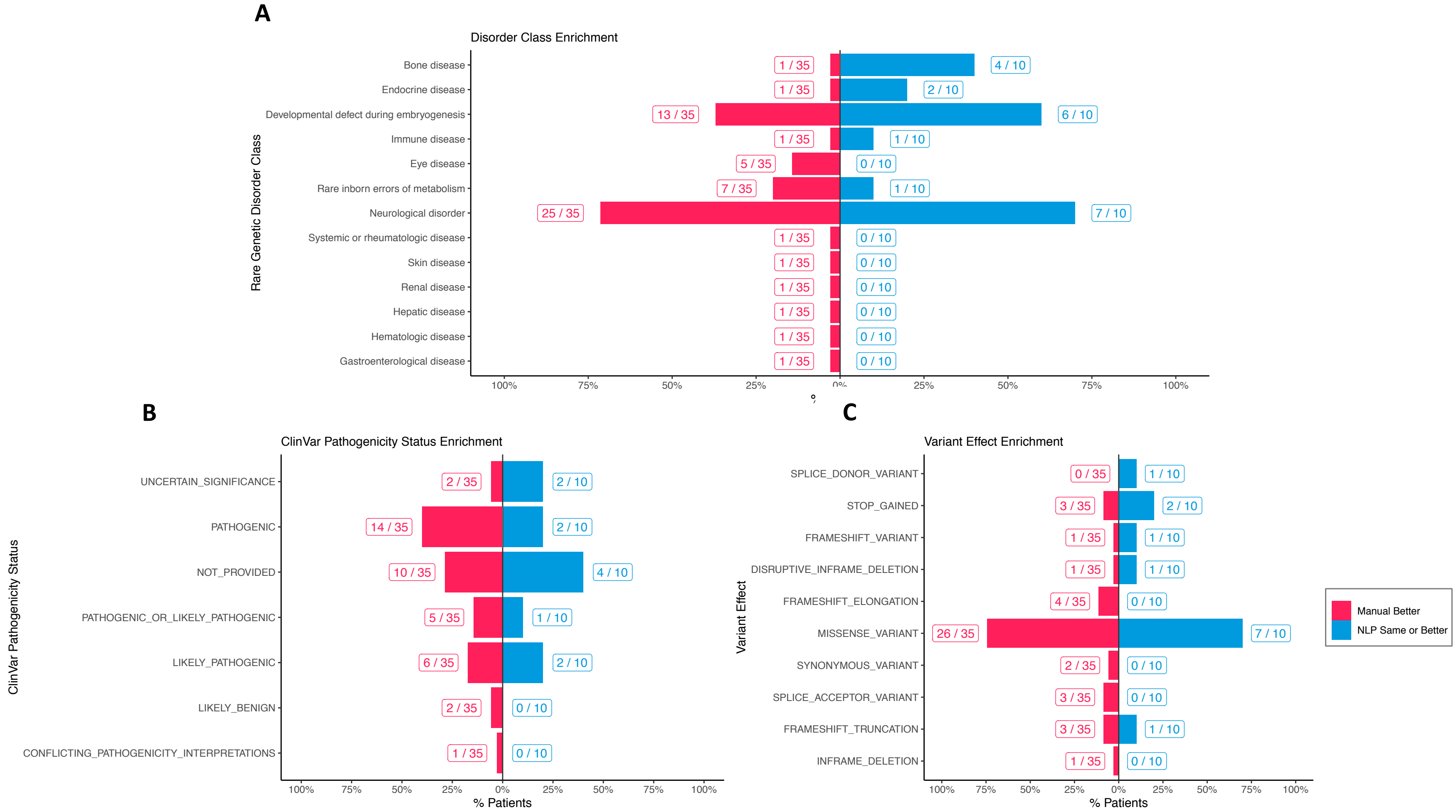

Figure S2. Comparing enrichment of disease and variant characteristics in patients grouped based on whether or not manually-extracted HPO terms led to better gene ranks in Exomiser

We compared (A) the Orphanet disorder class for the diagnosed disease, (B) the ClinVar pathogenicity status, and (C) the variant effect in the set of patients where manual phenotyping ranked better with the same attributes in the set of patients where NLP-based phenotyping ranked the same or better as the manual approach. These differences were not statistically significant. Statistical significance was determined if the p-value computed using Fisher's Exact Test with Benjamini-Hochberg multiple hypothesis correction was less than 0.05.

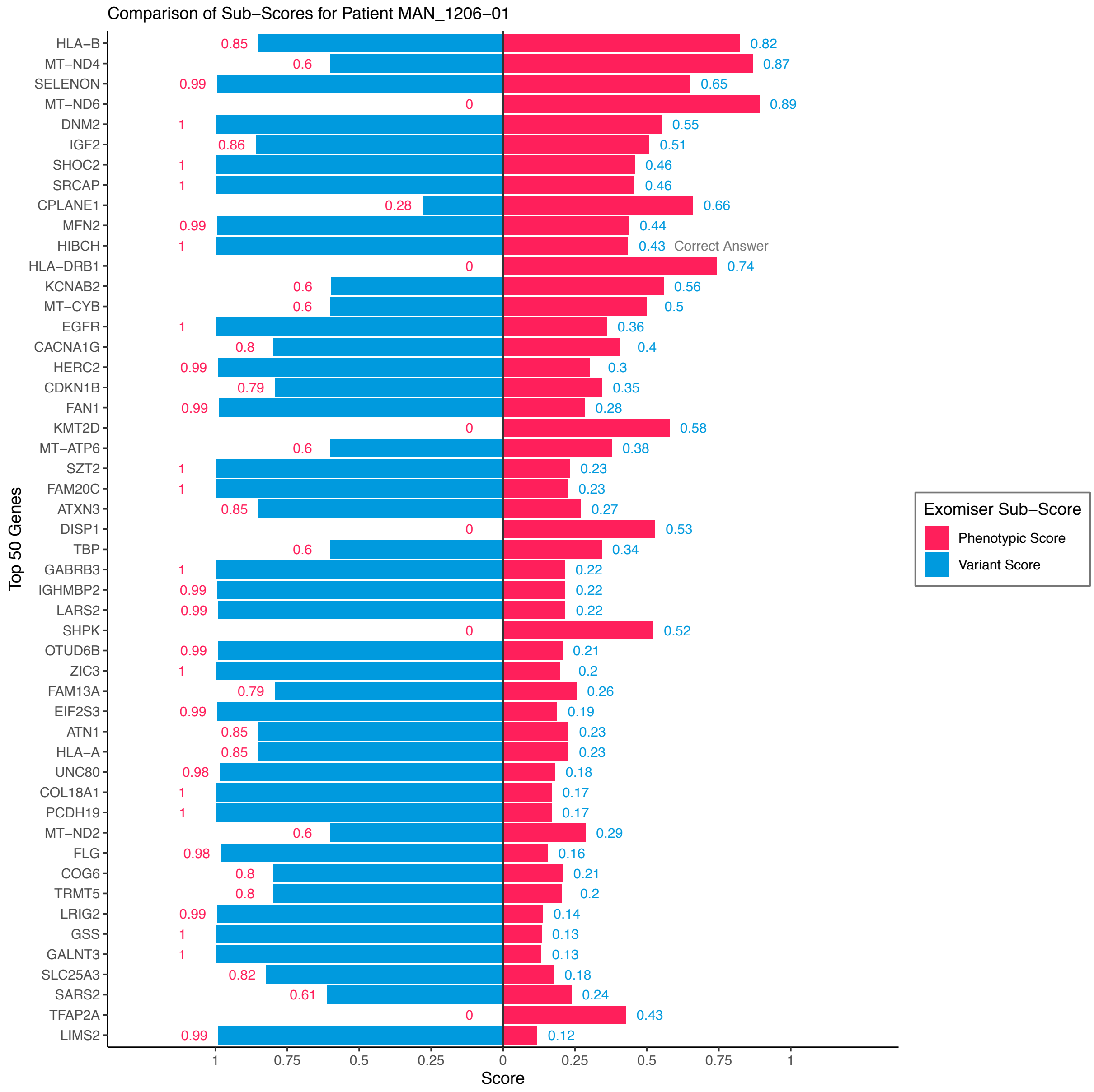

Figure S3. Exomiser phenotypic and variant sub-scores for the top 50 genes for a representative patient with known diagnosis

We consider top 50 to be a practical limit of genes that a clinician or researcher may manually evaluate. In the top 50 genes, the variant score was almost consistently high (> 0.8) and appears closer to a binary measure than a continuous one. On the other hand, the phenotypic score correlated with the gene rank. Therefore, we hypothesized that improved phenotyping may improve the desired gene rank. The correct causal gene for this patient was ranked 11. Simply prioritizing by the phenotypic score, however, would have dropped the ranking for the causal gene down to 17. In 4 of the 6 genes that had a higher phenotypic score, the variant score was 0 further supporting the idea that the variant score may serve as a binary admission criterion.

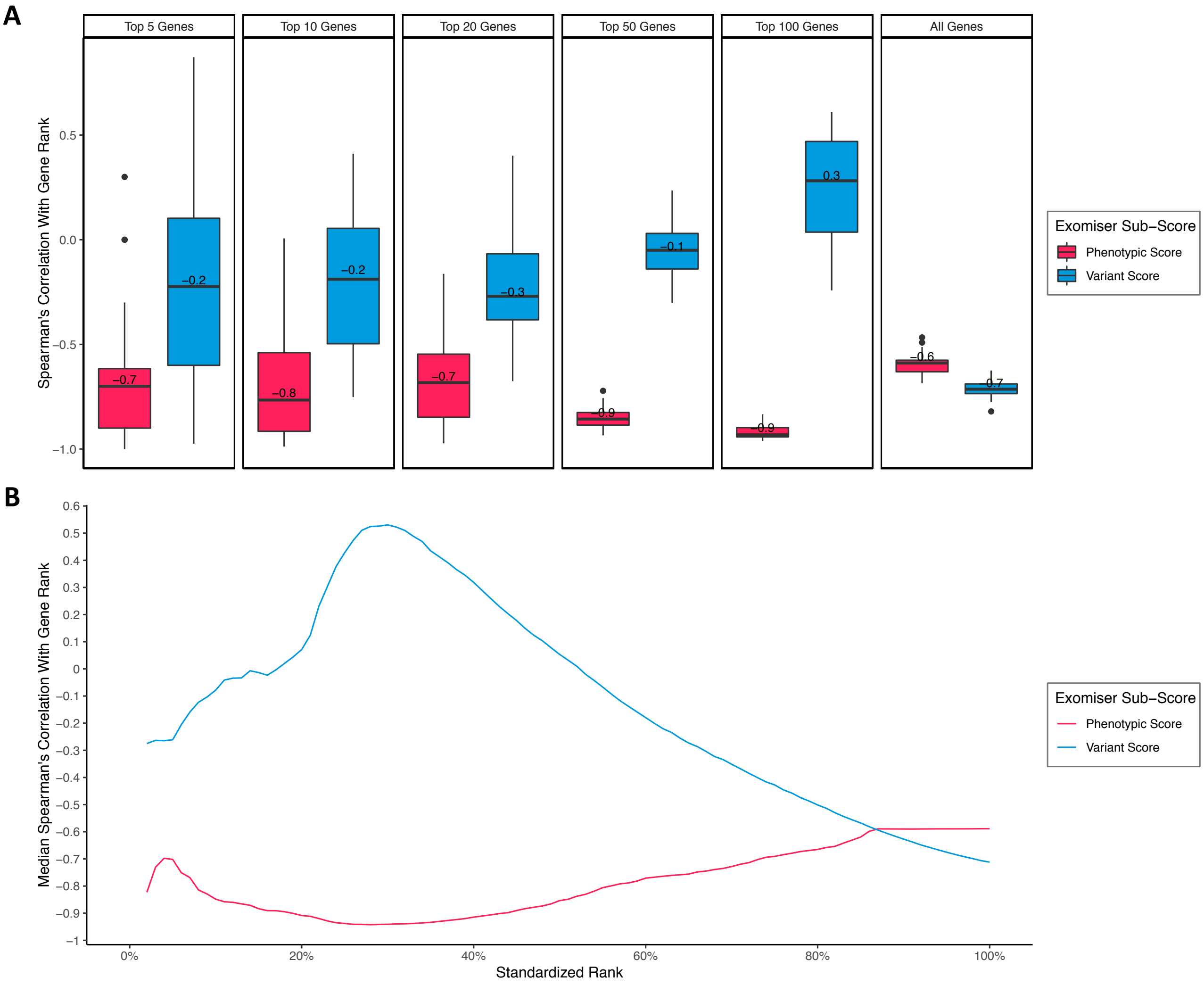

Figure S4. Distributions of correlations between gene ranks and sub-scores across patients in the training set

(A) To assess whether the observed correlation between phenotypic sub-score and gene rank was unique to the aforementioned example patient and in the top 50 genes, we computed the Spearman's correlation between each of the phenotypic and variant sub-scores with gene rank within the top 5, 10, 20, 50, 100, and entire gene lists for all 52 patients. Note that a correlation of -1 is the perfect desired correlation between a score (higher value is better) and gene rank (lower value is better). The distribution of variant sub-score correlations with gene rank in all set of top N genes (except for the entire gene list) crossed 0 (no correlation) and was consistently worse than the correlation between phenotypic sub-score and gene rank.

(B) The median correlation across 52 patients was computed between each of the phenotypic and variant sub-scores with gene rank for all possible gene rank cutoffs, standardized between 0 and 100%. The median correlation between variant score and gene rank is not better than the correlation between phenotypic score and gene rank until the tail end (bottom 15%) of the ranked genes.

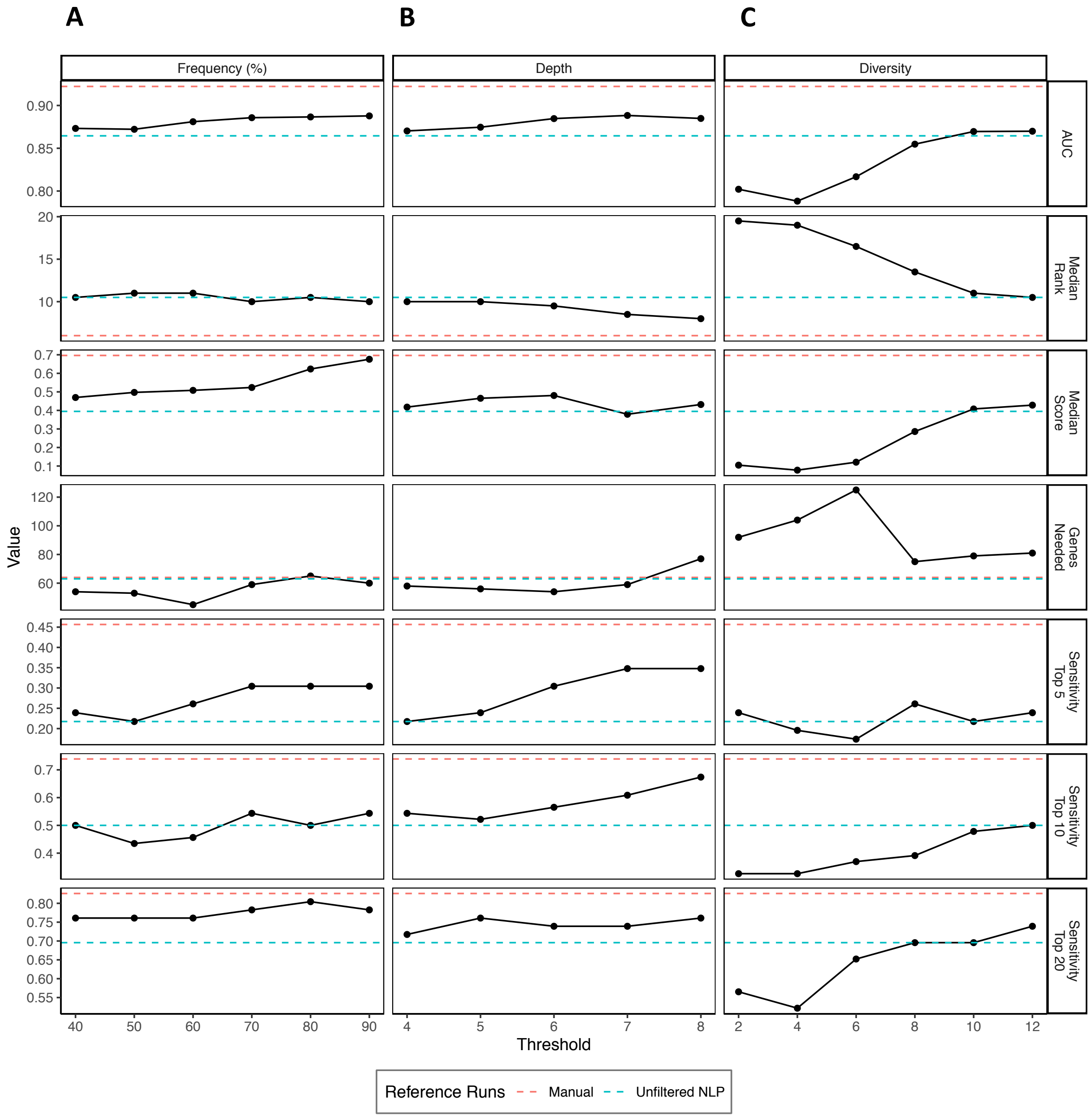

Figure S5. Exomiser performance on the training set after filtering NLP-extracted HPO terms per patient based on each term feature independently

(A) Performance after filtering for most frequently occurring HPO terms per patient as determined by frequency percentile thresholds between 40% and 90%.

(B) Performance after filtering for HPO terms per patient that are at least as deep as the specified depth threshold between 4 and 8.

(C) Performance after filtering for HPO terms per patient that belonging to the top n represented phenotypic abnormality classes where n is the diversity threshold ranging between 2 and 12, and phenotypic abnormality classes are ranked by mean frequency. Each row of plots corresponds to a different measure of Exomiser performance, *e.g.* the top row describes AUC as a function of varying the frequency, depth, and diversity thresholds respectively. The dashed red and blue lines in each plot indicate the Exomiser performance measure when using the manually-extracted and unfiltered NLP-extracted HPO terms respectively.

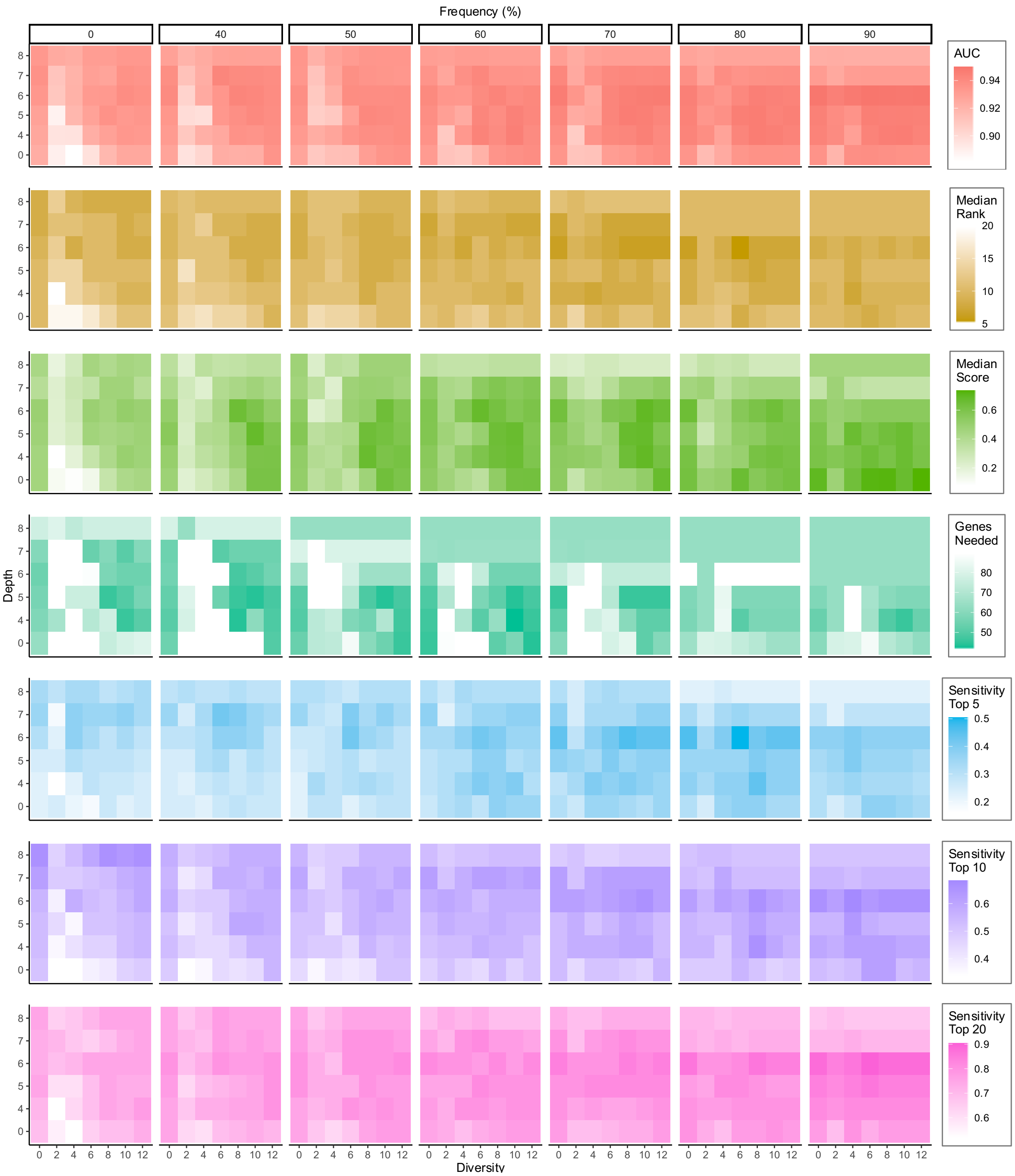

Figure S6. Exomiser performance on the training set after filtering NLP-extracted HPO terms per patient based on all possible combinations of term feature thresholds

There are 49 heatmap plots corresponding to 7 performance metrics (rows, colors) times 7 different frequency percentile thresholds (columns). Each heatmap plots the respective performance metric (color) for a given depth (y-axis) and diversity (x-axis) threshold. Therefore,

each row describes Exomiser performance as defined by a specific metric (*e.g.* AUC in the top row) for all possible 294 filter parameters determined by the combination of frequency percentile, depth, and diversity thresholds. Color scales are from minimum to maximum for each performance metric except for "Genes Needed", which was truncated to the interval 41 (actual minimum) to 90 (not the maximum) due to a few very high outliers.

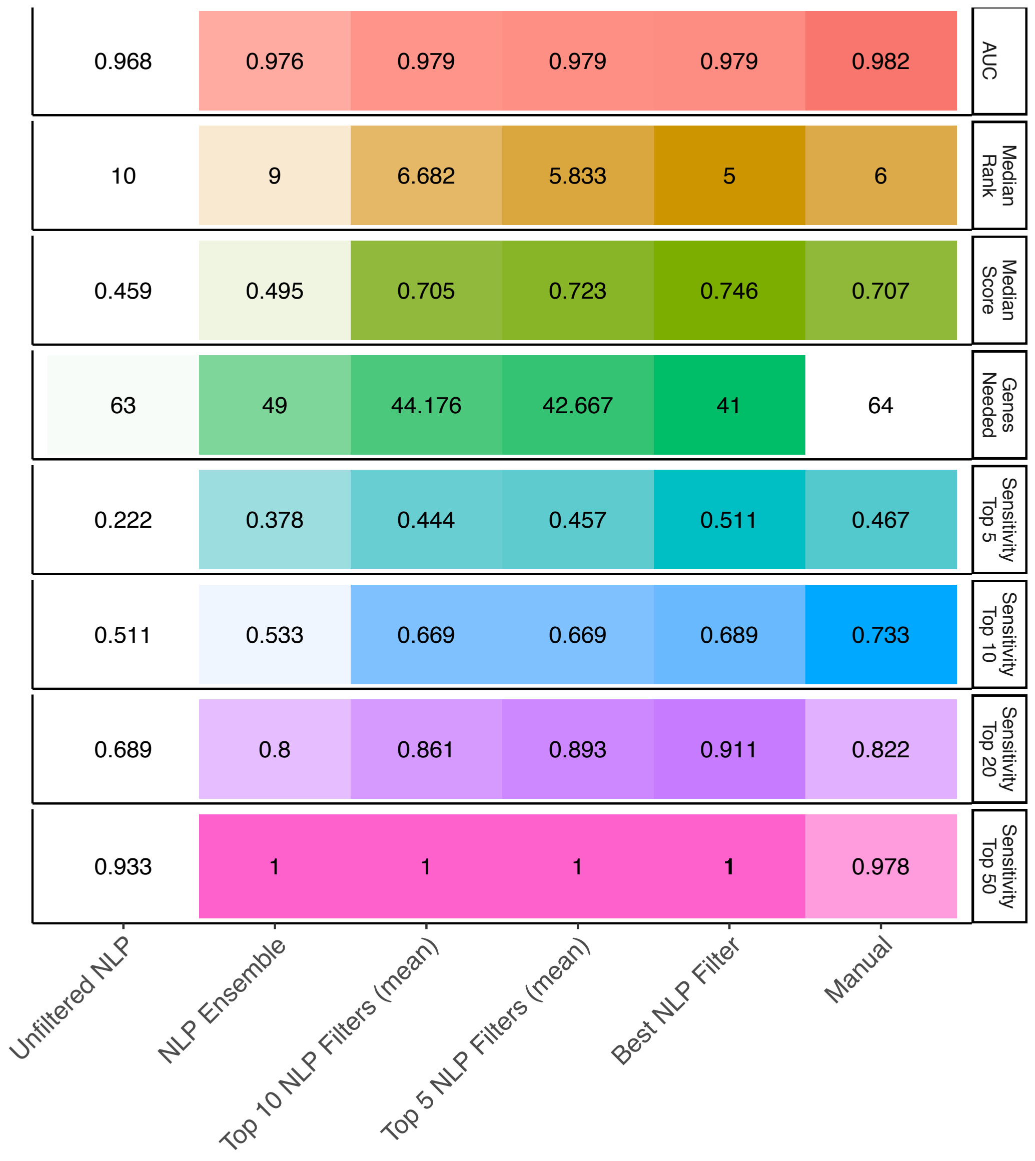

Figure S7. Exomiser performance on the training set for the best performing filter combinations compared to performance using manual and unfiltered NLP-based phenotype extraction

Each row (and color) corresponds to a different performance metric. The color intensity from light to dark represents better relative performance. The columns are bookended by Exomiser performance when using the unfiltered NLP-extracted and manual-extracted terms. The "NLP Ensemble" is based on the average score per gene across all 294 possible NLP filter parameter combinations. The "Best NLP Filter" represents a specific combination of filter parameters, which may vary per performance metric. The values in the "Top 5" and "Top 10" NLP Filters columns are the average of the top 5 and top 10 Exomiser performances per metric. Note that the number of parameter combinations corresponding to the top 5 and top 10 performances may be more that 5 and 10 respectively in the case of tied performance values. Similar to "Best NLP Filter", the sets of filter parameter combinations corresponding to the "Top 5" and "Top 10" performances may vary per metric.

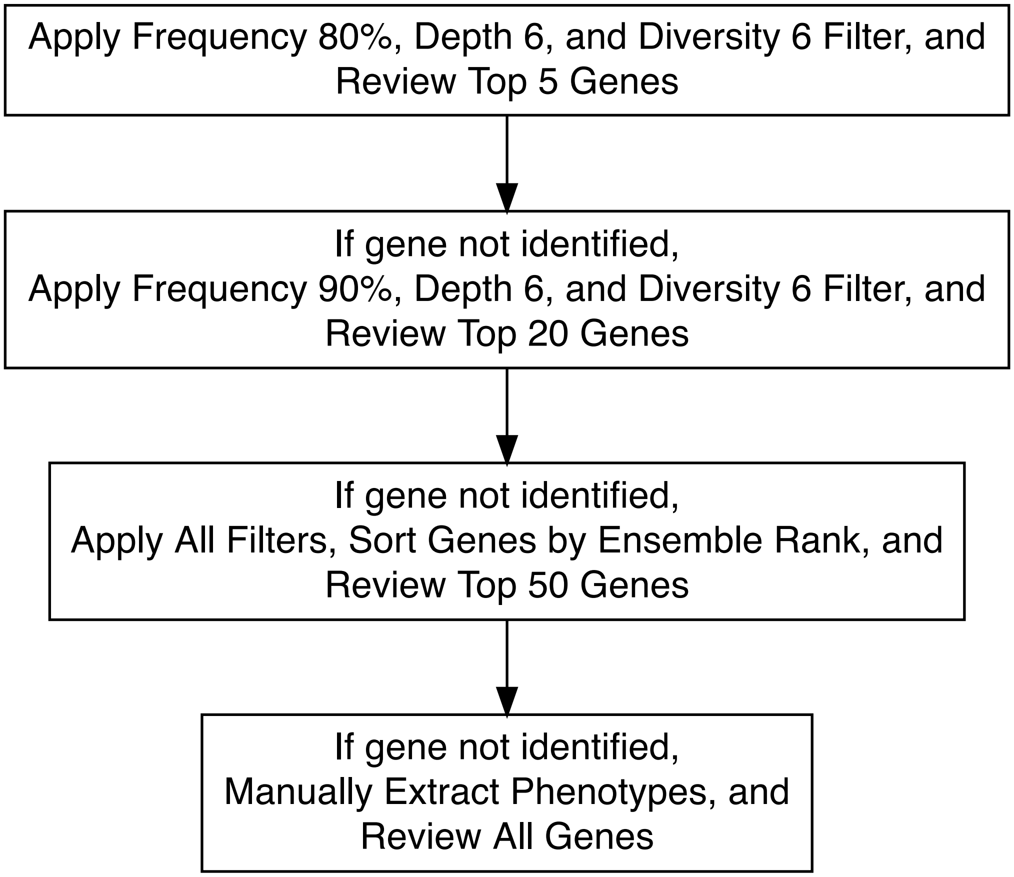

Figure S8. Flow chart describing the tiered approach to filtering NLP-extracted phenotypes and running gene prioritization

Based on our analysis of the effect of filtering NLP-extracted terms on improved gene prioritization, we constructed a 4-step tiered approach that incrementally increases the number of genes to consider.

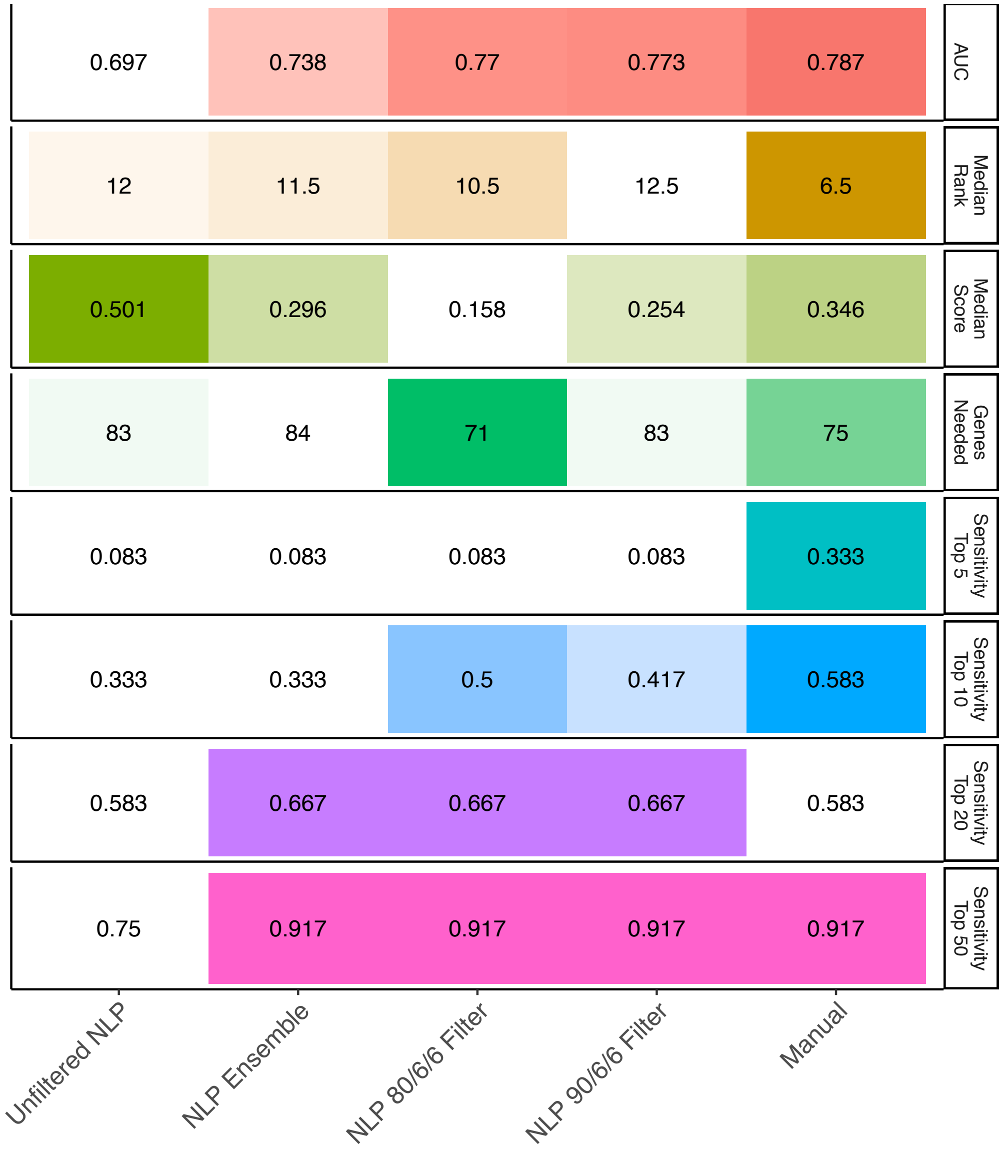

Figure S9. Exomiser performance on the test set for the filter combinations used in the tiered approach to phenotype extraction

Each row (and color) corresponds to a different performance metric. The color intensity from light to dark represents better relative performance. The algorithms (columns) are sorted by their overall performance (AUC) rather than the tier. “Unfiltered NLP” is included as a reference.

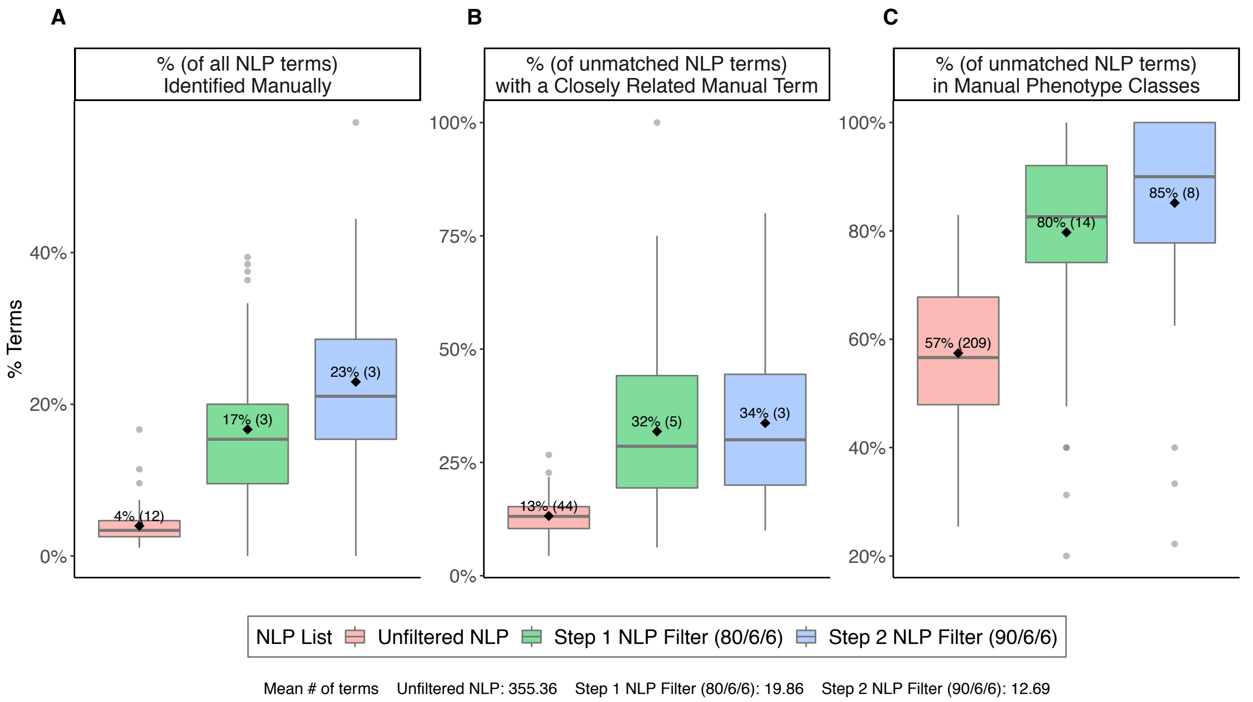

Figure S10. Box and whisker plots of distributions of percentage of manual and related terms before versus after NLP filtering
For each of the 64 patients in this study, we computed the (A) percentage of terms in the lists of unfiltered NLP terms, NLP terms remaining after the 80/6/6 filter, and NLP terms remaining after the 90/6/6 that were also identified manually. For the NLP terms that were not also identified manually, we computed for each patient the (B) percentage of those unmatched terms in the aforementioned lists that were closely related to a manually identified term (undirected ontological distance ≤ 2), and (C) the percentage of those unmatched terms that belonged to one of the phenotypic abnormality classes represented amongst the manually extracted terms. Mean percentages with the mean number of terms in parentheses are noted for each distribution. The mean number of terms per NLP list are noted below the figure legend for reference.

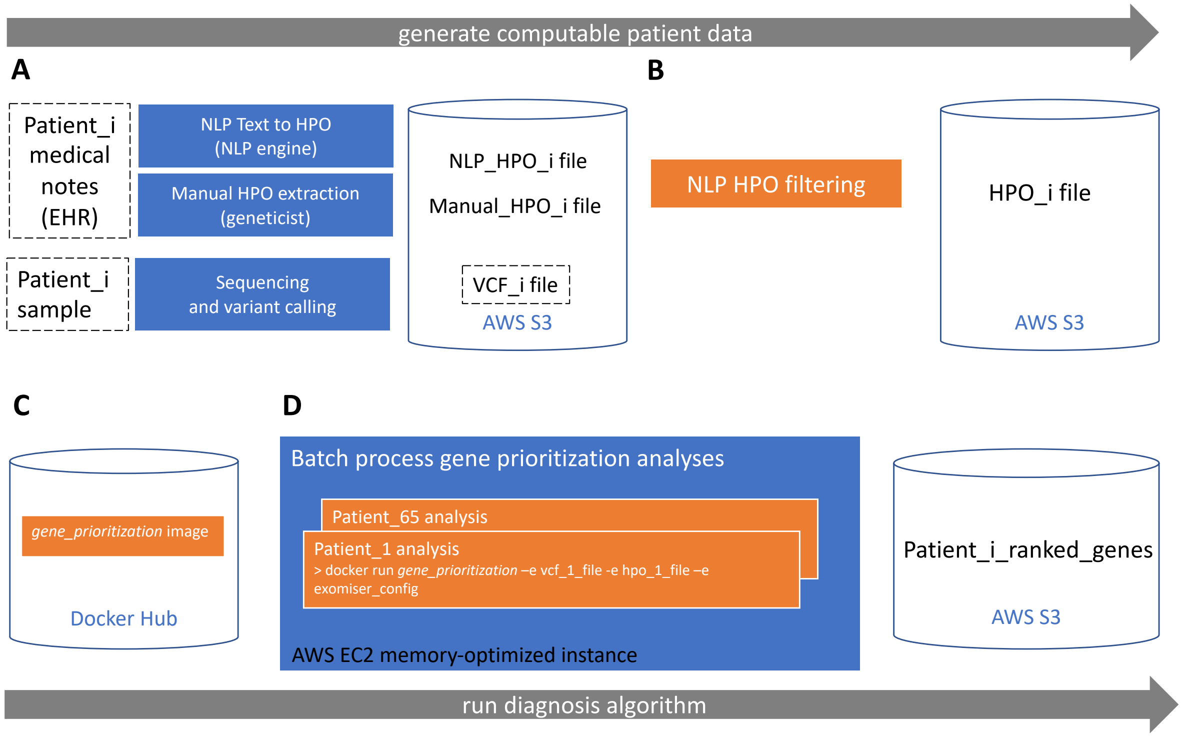

Figure S11. Modular architecture for implementing our phenotypic data-driven rare disease diagnosis pipeline

(A) For each patient i, the variant prioritization pipeline requires a VCF file describing the called variants and a list of HPO terms, extracted using NLP or by a manual expert curator. The files are stored within AWS S3. We used Clinithink's CLiX focus as our choice of NLP engine, which serves as a module with other options such as CLAMP.

(B) Custom post-processing scripts are then applied to the NLP-extracted HPO terms. We described 3 different filters to apply: frequency, depth, and diversity. Previous studies have shown the benefit of applying other filters such as removing common terms based on a reference set of EHRs[15], which could be applied at this step. The resulting post-processed HPO terms are stored in S3.

(C) The core variant prioritization tool is saved as docker[12] image in Docker Hub[13]. The containerized application takes as input a VCF file, a set of HPO terms, and configuration options and outputs a list of prioritized genes. We used Exomiser as our choice of gene prioritization tool, which could be replaced with other options such as MOON[17,18].

(D) We employ a single high memory AWS EC2 memory-optimized instance (r5n.16xlarge) to run batches of 65 patient-HPO set combinations at a time, with each run requiring 4GB memory (minimum 260GB memory required). The results are also stored in AWS S3, allowing the entire process to be run on the cloud.

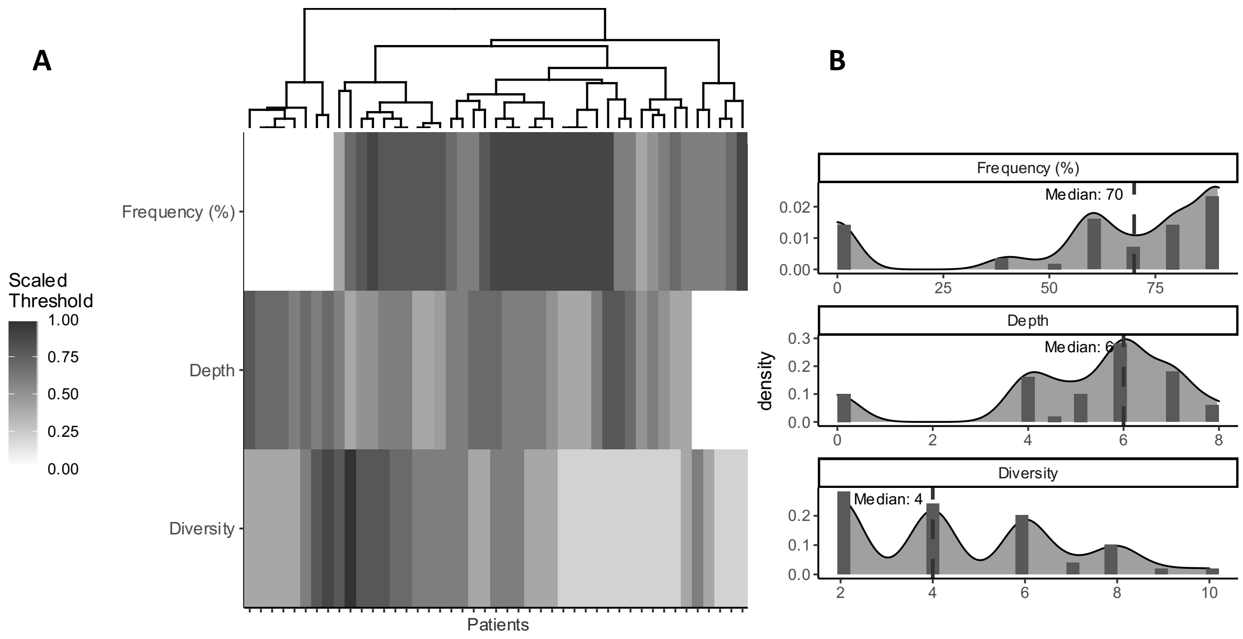

Figure S12. Patterns in parameter thresholds for optimal NLP filter combinations per patient in the training set

(A) Heatmap of the optimal filter combinations (x-axis) per patient (y-axis). Patients are hierarchically clustered based on the scaled values of the optimal filter combination thresholds. Optimal filter combinations are determined by gene rank first and then by the Exomiser score to break ties. If multiple combinations were still tied, we assigned the median value of the threshold per parameter to that patient as the optimal combination. The frequency threshold is scaled by a factor of 100. The depth and diversity thresholds are scaled by a factor of 10. All of the best diversity filter thresholds per patient were between 2-10 clades; none used the top 12 clades. Therefore, the resulting values in the heatmap have values between 0 and 1. There were 2 clear clusters of patients: a cluster of 7 patients with an absent frequency filter and a cluster of 5 patients with an absent depth threshold. Other than the aforementioned 2 clusters, there were no discerning patterns in the optimal thresholds or distinct clusters of patients.

(B) Distributions (probability densities) with median threshold value listed for each of the three parameters comprising the optimal filter combinations per patient. The median values for the frequency percentile, depth, and diversity thresholds in the optimal filter combinations were 70, 6, and 6 respectively.

SUPPLEMENTAL TABLES

Table S1. Genotypes of the probands in the training and test sets.

Supplementary_subject_genotypes.xlsx
Genotypes of the 53 probands in the training set and 12 probands in the test set. Available online at the Journal website.

Table S2. Patient demographics.

	N	% female (n)	Mean age (years)	Median year sequenced (range)	Mean no. of manual HPO terms (range)	Mean no. of NLP HPO terms (range)
Training Set	52	57% (30)	8.13 (0.05-24)	2014 (2012-2018)	15.1 (4-32)	379.8 (70-735)
Test Set	12	25% (3)	6.2 (0.04-16.25)	2017 (2014-2020)	12.6 (7-25)	249.5 (18-561)

Table S3. Depth and phenotypic abnormality classes per HPO term.

Table_S3_HPO_depth_abnormality_classes.xlsx
The depth and phenotypic abnormality classes per HPO term calculated using the shortest directed path (unweighted breadth-first search) from the root node of the HPO directed acyclic graph. Available online at the Journal website.

Table S4. Breakdown of patients in the training set by relative Exomiser rankings of the correct gene using manual- versus NLP-based phenotype extraction.

Exomiser Correct Gene Rank Comparison	Number of Patients (%)
Manual Ranked Better	35 (77.8%)
NLP Ranked Better	5 (11.1%)
Manual and NLP Ranked the Same	5 (11.1%)
Total Patients	45

Table S5. Performance of all 294 combination filters.

Table_S5_all_combinations_performance_landscape.xlsx
All 7 performance metrics (AUC, Median Rank, Median Score, Genes Needed, Sensitivity in the top 5 genes, Sensitivity in the top 10 genes, and Sensitivity in the top 20 genes for each of the 294 NLP combination filters and the manually phenotyping for reference. NLP combination filters (column “run_name”) are described by the frequency percentile threshold, depth, and diversity with prefixes “fp”, “d”, and “c” respectively. The thresholds are also explicitly listed. Available online at the Journal website.

Table S6. Parameter combinations for the top performing NLP filters applied to the training set.*

Filtering Criteria for Top Combinations	Best NLP Filter			Top 5 NLP Filters Median (MAD)			Top 10 NLP Filters Median (MAD)		
	Frequency (%)	Depth	Diversity	Frequency (%)	Depth	Diversity	Frequency (%)	Depth	Diversity
AUC	90	6	6	90 (0)	6 (0)	8 (2.97)	90 (0)	6 (0)	8 (2.97)
Median Rank	80	6	6	70 (0)	6 (0)	7 (5.93)	70 (7.41)	6 (0.74)	8 (4.45)
Median Score	90	0	12	90 (0)	0 (0)	8 (2.97)	70 (22.24)	2 (2.97)	10 (2.97)
Genes Needed	60	4	10	55 (7.41)	4.5 (0.74)	10 (0)	60 (14.83)	5 (0)	10 (2.97)
Sensitivity Top 5	80	6	6	80 (0)	6 (0)	8 (2.97)	70 (14.83)	6 (0)	7 (1.48)
Sensitivity Top 10	90	6	4	80 (14.83)	6 (0)	8 (2.97)	80 (14.83)	6 (0)	8 (2.97)
Sensitivity Top 20	90	6	6	90 (0)	6 (0)	8 (2.97)	90 (0)	6 (0)	8 (5.93)
Overall Median (MAD)	90 (0)	6 (0)	6 (0)	80 (14.83)	6 (0)	8 (2.97)	70 (14.83)	6 (0)	8 (2.97)

*Note that Top 5/10 may have more than 5 or 10 filter combinations if they are tied. Therefore, the grand median (and analogous for MAD) for the top 5/10 may be different than the median of medians.

Table S7. Rank of the correct gene per patient in the prospectively evaluated test set using different phenotype extraction and filters.

Patient	NLP	NLP Ensemble	NLP 80/6/6 Filter	NLP 90/6/6 Filter	Manual	Best NLP Filter Rank	Change in Rank Post Filtering
MAN_1787	11	11	9	11	7	9	-2
MAN_1845	59	38	37	30	25	30	-29
MAN_1795	22	24	22	22	21	22	0
MAN_1802	2	1	2	2	2	1	-1
MAN_0943	12	6	7	14	6	6	-6
MAN_1011	83	84	71	83	75	71	-12
MAN_0469	7	7	7	6	6	6	-1
MAN_0805	57	49	42	26	34	26	-31
MAN_0842	29	20	13	6	33	6	-23
MAN_0678	12	12	9	18	2	9	-3
MAN_0520	10	11	12	10	5	10	0
MAN_1886	7	7	7	7	5	7	0

Table S8. The number of patients where each NLP combination filter was applicable.

Table_S8_num_patients_filtered_histogram.xlsx
The number of patients that each NLP combination filter was applied to. If a combination filter led to fewer than 5 HPO terms remaining for a given patient, then the combination filter was not applied to that patient. Especially stringent combination filters (e.g. limited to 90% frequency percentile, at least 8 levels deep, and top 2 abnormality classes) result in very few patients with filtered terms (column “num_patients_filtered”). The combination filter (column “run”) is described by the frequency percentile threshold, depth, and diversity with prefixes “fp”, “d”, and “c” respectively. Available online at the Journal website.

Table S9. Optimal phenotype extraction option based on gene rank for patients in the training set.

Optimal Phenotype Extraction Option	Number of Patients (%)
Best NLP Filter	33 (73.3%)
Best NLP Filter, or NLP Ensemble	3 (6.7%)
Best NLP Filter, or Manual	3 (6.7%)
Any	3 (6.7%)
Manual	2 (4.4%)
Best NLP Filter, or Unfiltered NLP	1 (2.2%)
Total	45

Table S10. Spearman's correlations between parameter thresholds for optimal NLP filter combinations per patient in the training set.*

Spearman Correlation (P-value)	Frequency (%) Threshold	Depth Threshold
Depth Threshold	-0.29 (0.052)	
Diversity Threshold	-0.08 (0.614)	0.11 (0.475)

*The two clusters of patients with absent frequency percentile and depth thresholds results in the strongest correlation between any two parameters. However, no pairs of parameters had statistically significant correlations (p-value < 0.05). The p-value for Spearman's correlation is computed using algorithm AS 89[24].

Table S11. Cross-tabulation of training set patients based on HPO-term extraction methods resulting in higher gene ranks before and after filtering.*

	Number of Patients (%) Optimal NLP Filter					
Optimal Pre-Filtering Term Extraction Method	[80 or 90]/6/6	80/6/6	90/6/6	Ensemble	Manual	Total
Manual	1 (2.86%)	10 (28.57%)	8 (22.86%)	0 (0%)	16 (45.71%)	35 (77.78%)
Unfiltered NLP	3 (30%)	1 (10%)	5 (50%)	1 (10%)	0 (0%)	10 (22.22%)
Total	4 (8.89%)	11 (24.44%)	13 (28.89%)	1 (2.22%)	16 (35.56%)	45

*Table rows divide patients based on whether manual- or NLP-extracted terms without filtering resulted in higher gene ranks (Table S4). Table columns divide patients based on which NLP filter from the tiered pipeline resulted in the highest gene ranks. Manual extraction is included in the post-filtering categories for reference. Ties in rank were resolved based on feasibility (Combination Filters [80/6/6 or 90/6/6] > Ensemble > Unfiltered NLP > Manual).
